# Supplementary material for: Characterizing gene tree conflict in plastome-inferred phylogenies
Source: PeerJ. 2019 Sep 24;7:e7747. doi: 10.7717/peerj.7747 (PMC6764362; doi:10.7717/peerj.7747)

Figure S1: Each diagram represents a different molecule type and shows the proportion of concordance each gene exhibits at the five time slices shown in Fig. 1: (1) 150–120 mya, (2) 120–90 mya, (3) 90–60 mya, (4) 60–30 mya, (5) 30–0 mya and C is the concordance summed over all the time scales. The individual genes are scaled by length of alignment; however, *ycf1* and *ycf2* are cut to approximately the length of *rpoC2* due to their abnormally long alignments. Genes are ordered based the order of genes in the *Nicotiana sylvestris* plastome.

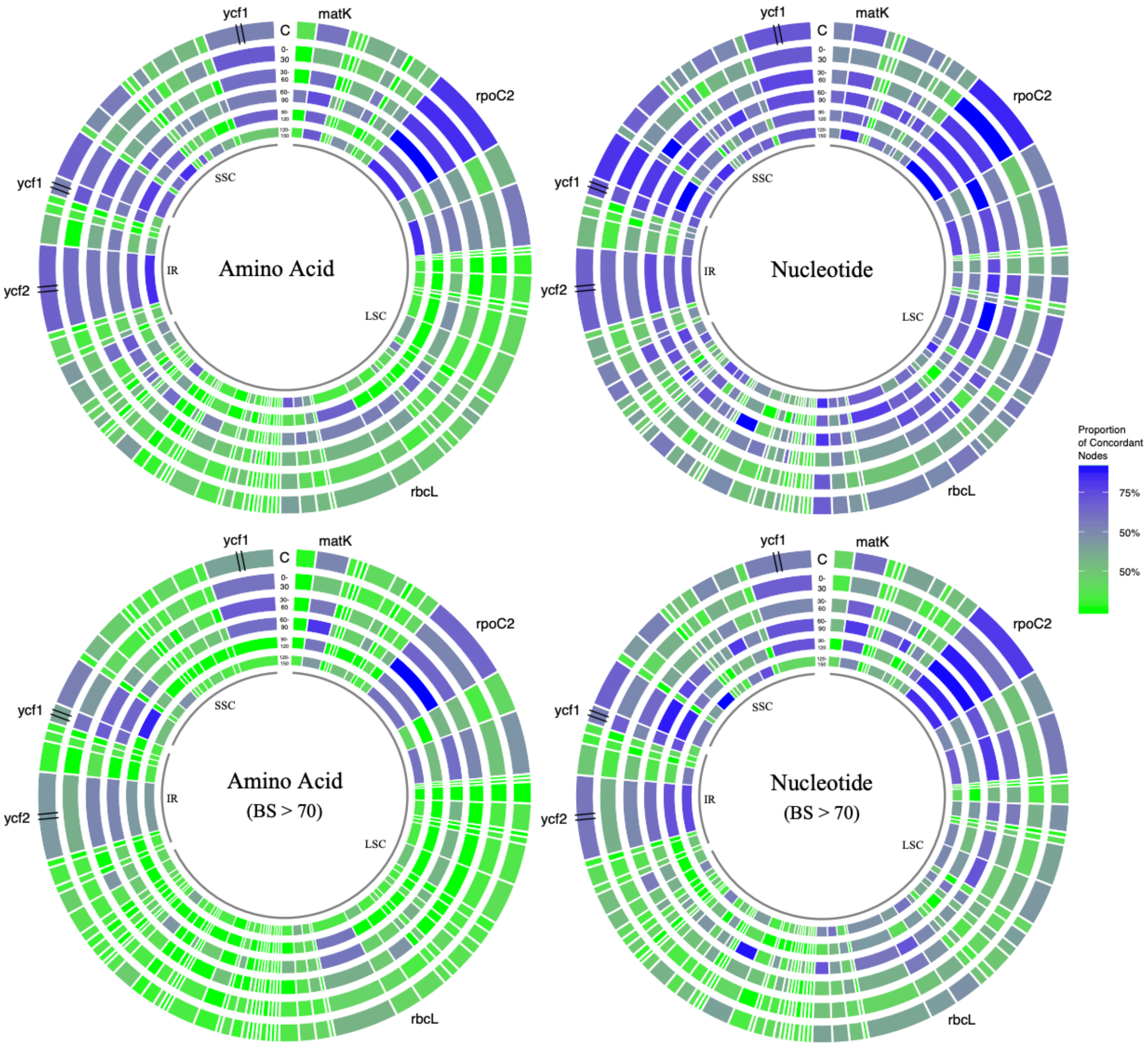

Supplement: Figure S1 — Each diagram represents a different molecule type and shows the proportion of concordance each gene exhibits at the five time slices shown in Fig. 1: (1) 150–120 mya, (2) 120–90 mya, (3) 90–60 mya, (4) 60–30 mya, (5) 30–0 mya and C is the concordance summed over all the time scales. The individual genes are scaled by length of alignment; however, ycf1 and ycf2 are cut to approximately the length of rpoC2 due to their abnormally long alignments. Genes are ordered based the order of genes in the Nicotiana sylvestris plastome. [file peerj-07-7747-s001.pdf]
