# Supplementary material for: Characterizing gene tree conflict in plastome-inferred phylogenies
Source: PeerJ. 2019 Sep 24;7:e7747. doi: 10.7717/peerj.7747 (PMC6764362; doi:10.7717/peerj.7747)

Figure S3: Inferred amino acid gene trees for the 79 concatenated plastome genes. Branches colored in red are discordant with the accepted topology (AT). Pie charts depict the amount of gene tree conflict observed in the amino acid gene tree analysis, with the blue, red, green, and gray slices representing, respectively, the proportion of gene trees concordant, conflicting (supporting a single main alternative topology), conflicting (supporting various alternative topologies), and uninformative (BS < 70 or missing taxon) at each node in the species tree.

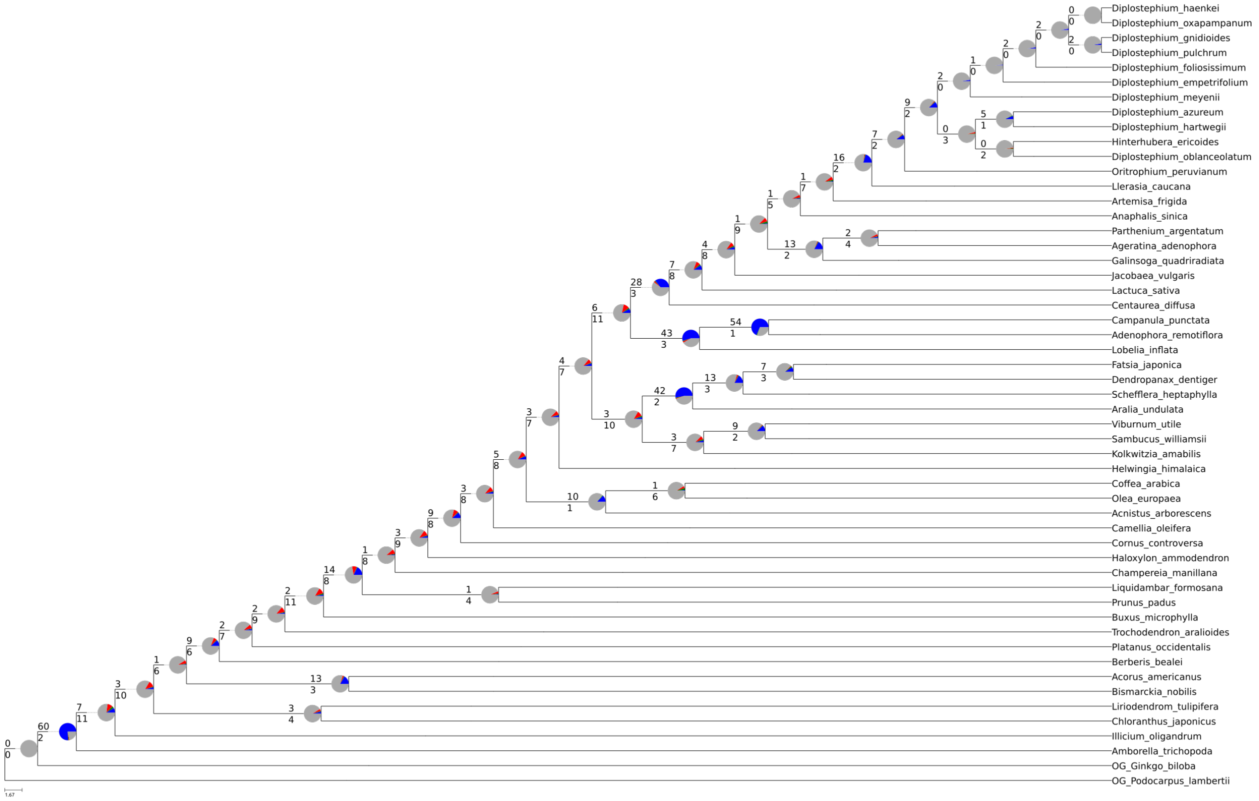

Supplement: Figure S3 — Inferred amino acid gene trees for the 79 concatenated plastome genes. Branches colored in red are discordant with the accepted topology (AT). Pie charts depict the amount of gene tree conflict observed in the amino acid gene tree analysis, with the blue, red, green, and gray slices representing, respectively, the proportion of gene trees concordant, conflicting (supporting a single main alternative topology), conflicting (supporting various alternative topologies), and uninformative (BS < 70 or missing taxon) at each node in the species tree. [file peerj-07-7747-s003.pdf]
