# Supplementary material for: Characterizing gene tree conflict in plastome-inferred phylogenies
Source: PeerJ. 2019 Sep 24;7:e7747. doi: 10.7717/peerj.7747 (PMC6764362; doi:10.7717/peerj.7747)
Supplement: Figure S5 — Saturation plots for each codon position and for the entire gene. [file peerj-07-7747-s005.pdf]

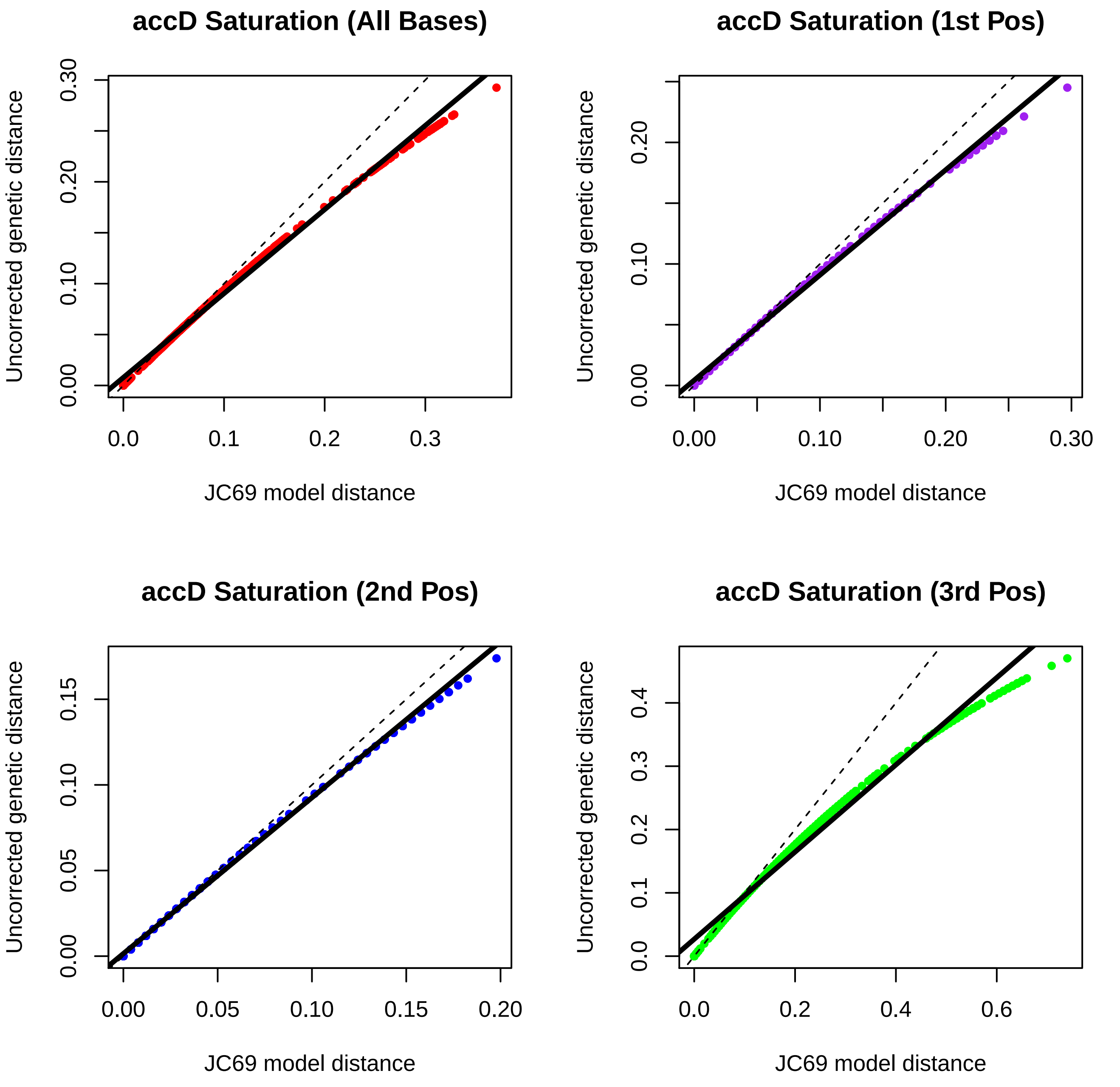
accD Saturation (All Bases)
accD Saturation (1st Pos)
accD Saturation (2nd Pos)
accD Saturation (3rd Pos)
Uncorrected genetic distance
JC69 model distance
0.00
0.10
0.20
0.30
0.0
0.1
0.2
0.3
0.00
0.10
0.20
0.00
0.10
0.20
0.30
0.00
0.05
0.10
0.15
0.00
0.05
0.10
0.15
0.20
0.0
0.1
0.2
0.3
0.4
0.0
0.2
0.4
0.6

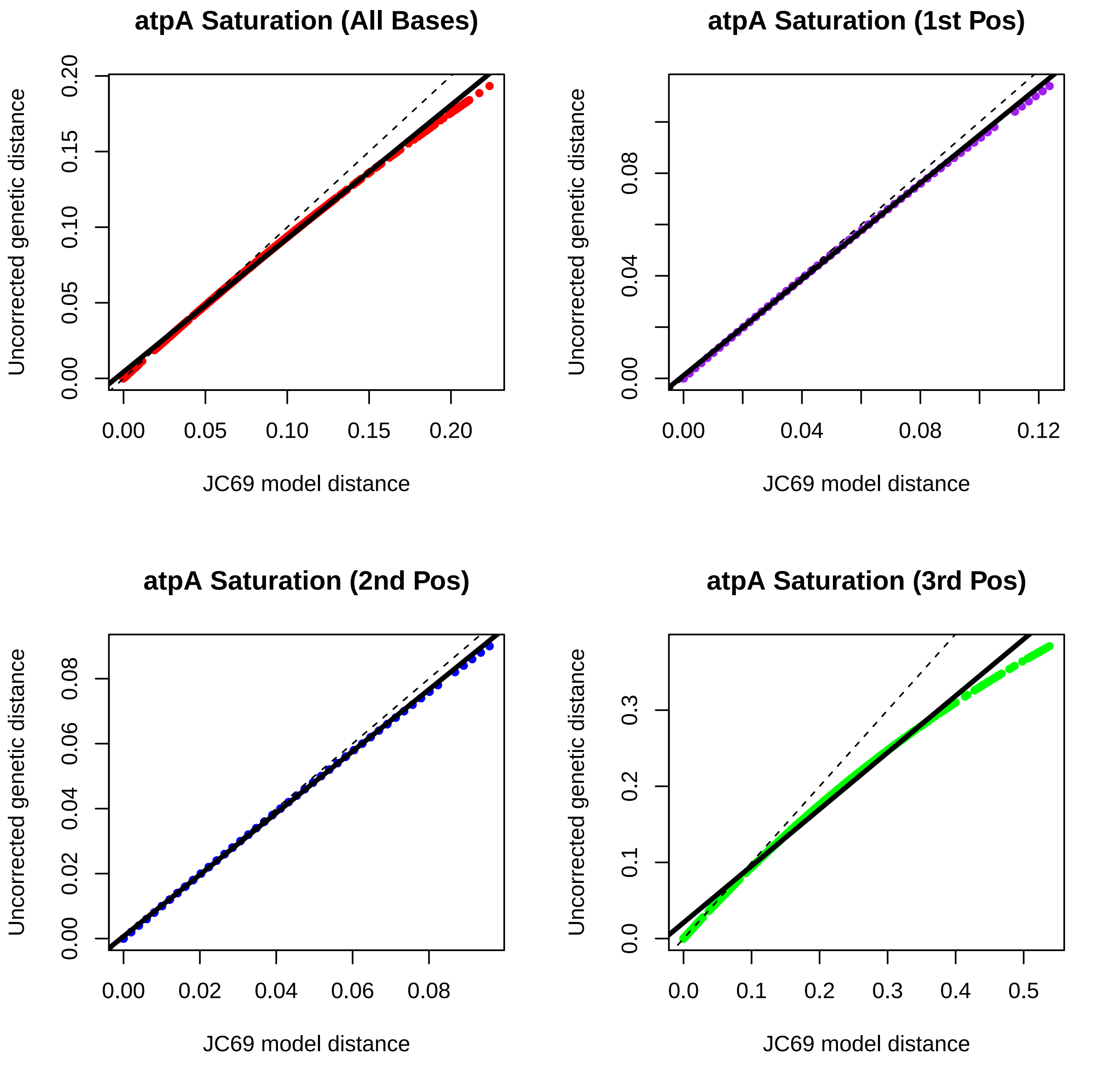

atpA Saturation (All Bases)
Uncorrected genetic distance
JC69 model distance
0.00
0.05
0.10
0.15
0.20
atpA Saturation (1st Pos)
Uncorrected genetic distance
JC69 model distance
0.00
0.04
0.08
0.12
atpA Saturation (2nd Pos)
Uncorrected genetic distance
JC69 model distance
0.00
0.02
0.04
0.06
0.08
atpA Saturation (3rd Pos)
Uncorrected genetic distance
JC69 model distance
0.0
0.1
0.2
0.3
0.4
0.5

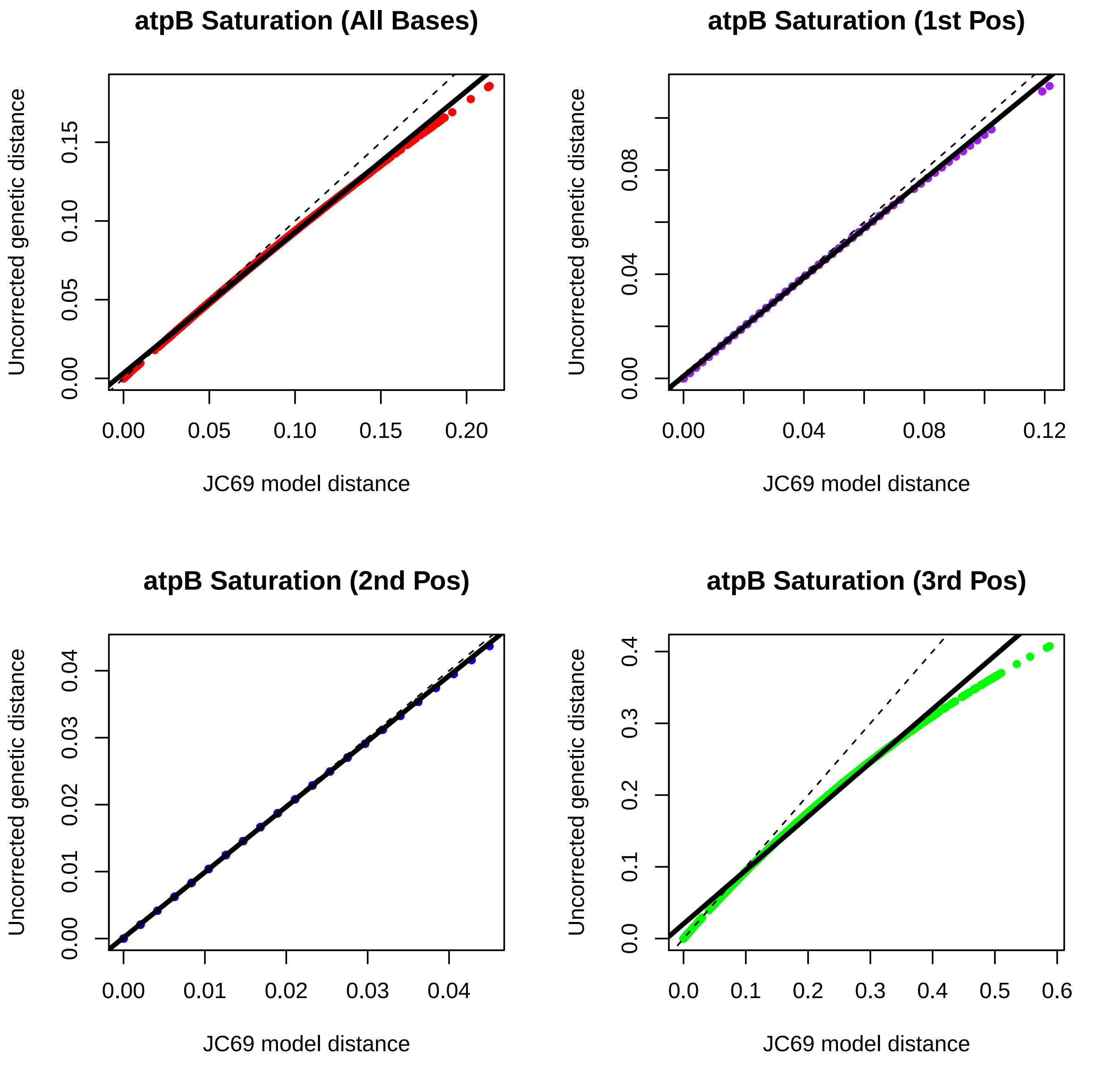
atpB Saturation (All Bases)
atpB Saturation (1st Pos)
atpB Saturation (2nd Pos)
atpB Saturation (3rd Pos)
Uncorrected genetic distance
JC69 model distance
0.00
0.05
0.10
0.15
0.20
0.04
0.08
0.12
0.01
0.02
0.03
0.0
0.1
0.2
0.3
0.4
0.5
0.6

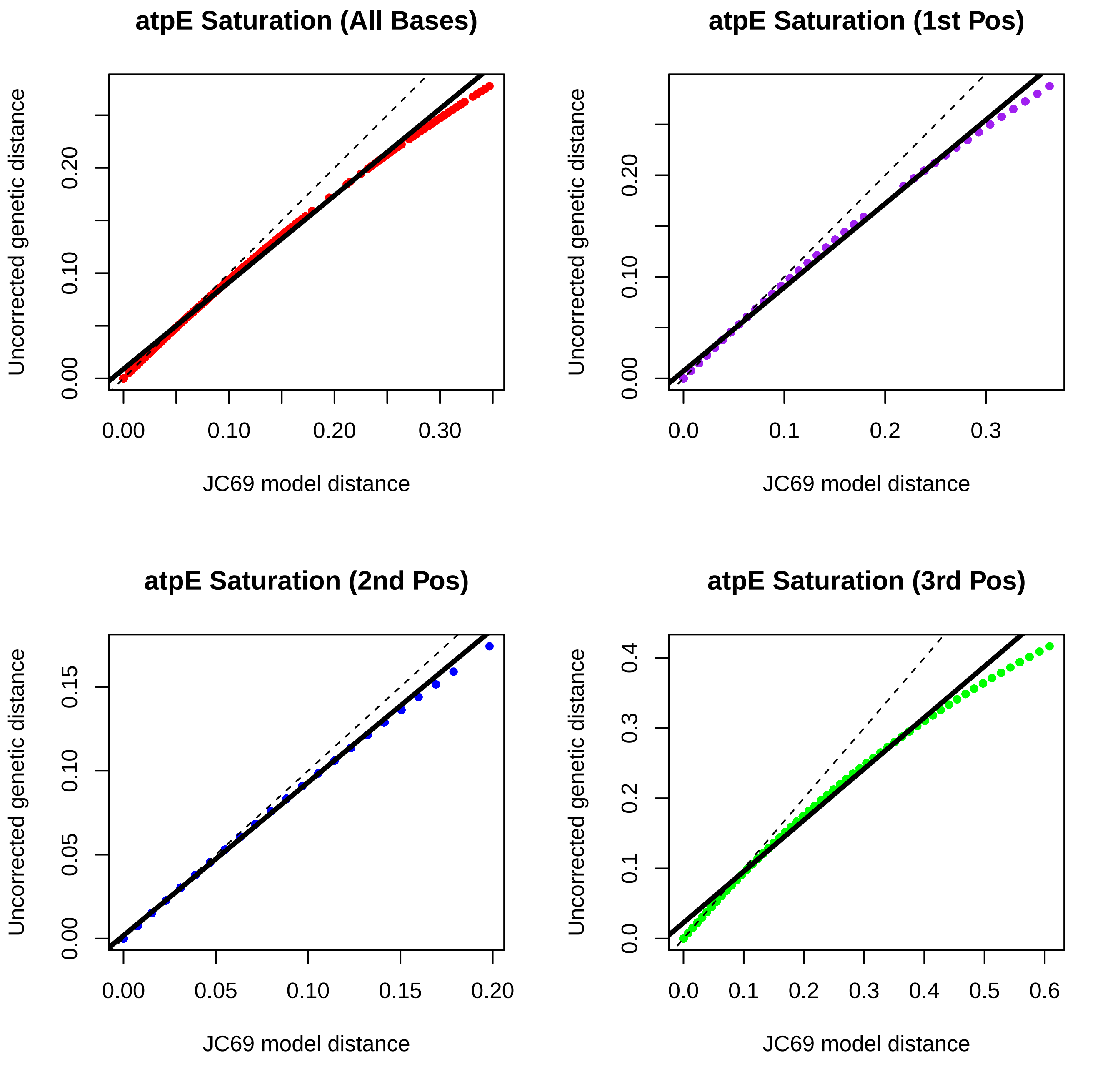
atpE Saturation (All Bases)
Uncorrected genetic distance
0.00
0.10
0.20
0.00
0.10
0.20
0.30
JC69 model distance
atpE Saturation (1st Pos)
Uncorrected genetic distance
0.00
0.10
0.20
0.0
0.1
0.2
0.3
JC69 model distance
atpE Saturation (2nd Pos)
Uncorrected genetic distance
0.00
0.05
0.10
0.15
0.00
0.05
0.10
0.15
0.20
JC69 model distance
atpE Saturation (3rd Pos)
Uncorrected genetic distance
0.0
0.1
0.2
0.3
0.4
0.0
0.1
0.2
0.3
0.4
0.5
0.6
JC69 model distance

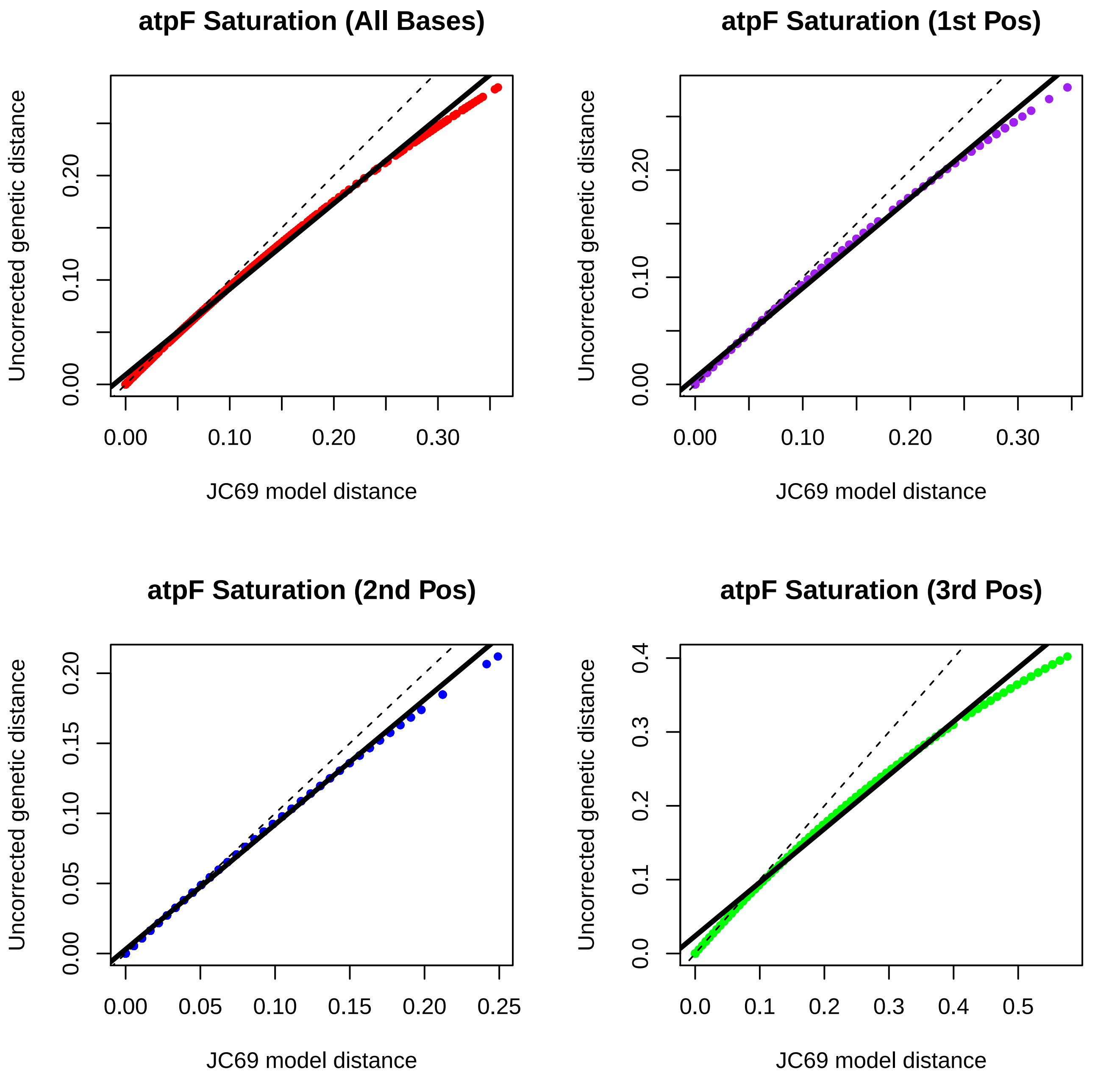
atpF Saturation (All Bases)
atpF Saturation (1st Pos)
atpF Saturation (2nd Pos)
atpF Saturation (3rd Pos)
Uncorrected genetic distance
JC69 model distance
0.00
0.10
0.20
0.30
0.05
0.15
0.25
0.0
0.1
0.2
0.3
0.4
0.5

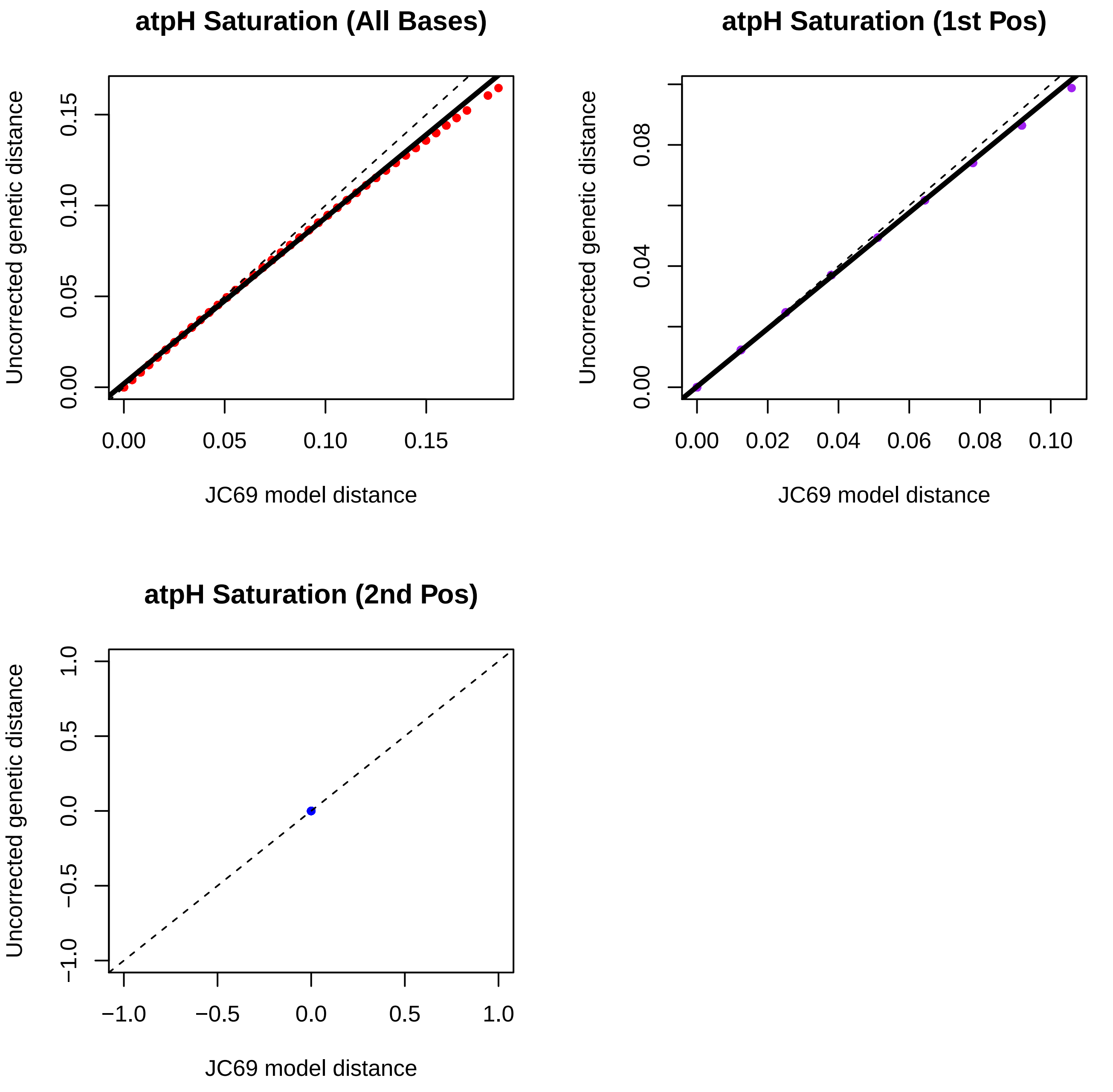
atpH Saturation (All Bases)
Uncorrected genetic distance
JC69 model distance
0.00
0.05
0.10
0.15
atpH Saturation (1st Pos)
Uncorrected genetic distance
JC69 model distance
0.00
0.04
0.08
0.00
0.02
0.04
0.06
0.08
0.10
atpH Saturation (2nd Pos)
Uncorrected genetic distance
JC69 model distance
−1.0
−0.5
0.0
0.5
1.0

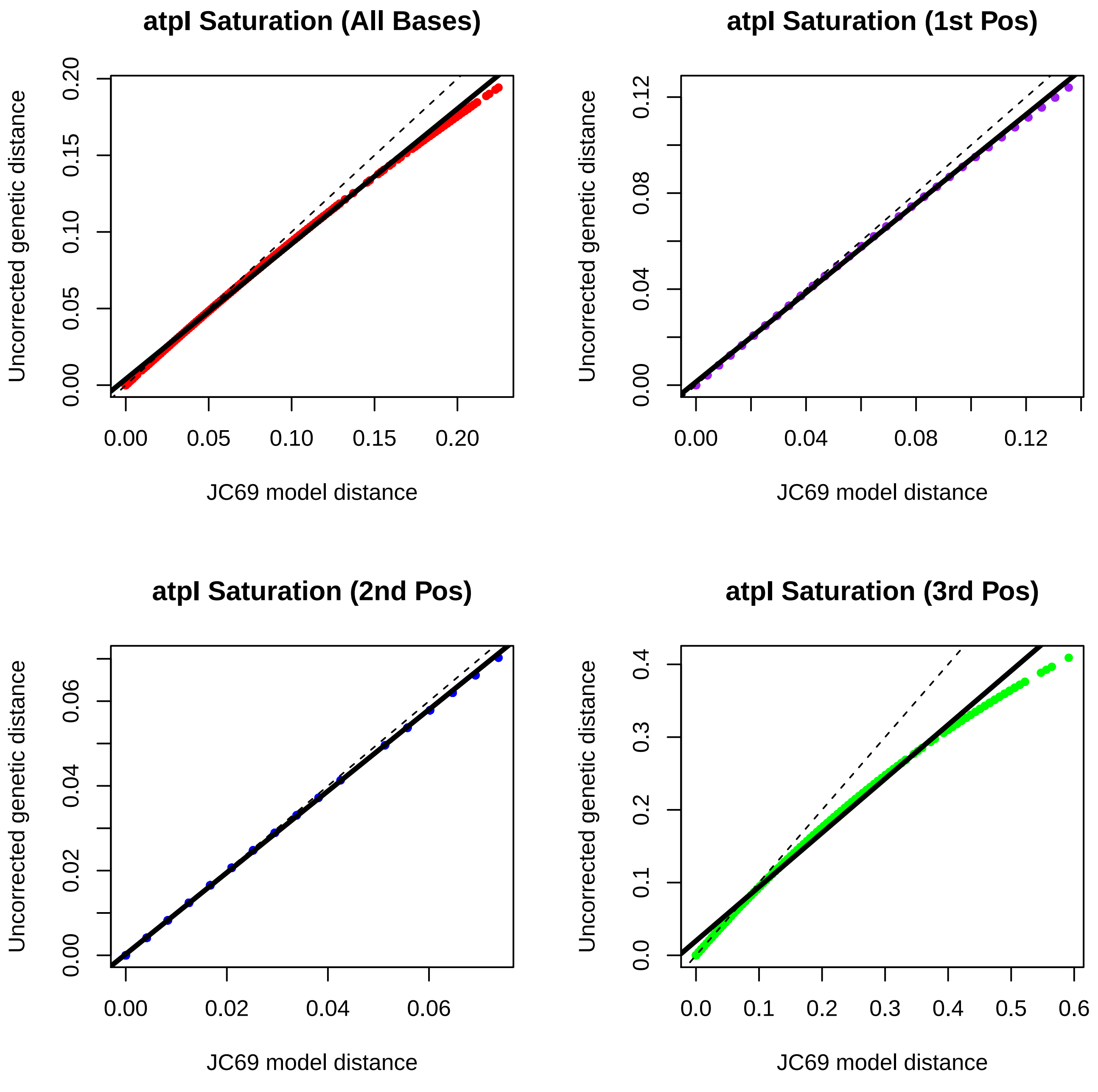

atpI Saturation (All Bases)
Uncorrected genetic distance
JC69 model distance
0.00
0.05
0.10
0.15
0.20
atpI Saturation (1st Pos)
0.00
0.04
0.08
0.12
atpI Saturation (2nd Pos)
0.00
0.02
0.04
0.06
atpI Saturation (3rd Pos)
0.0
0.1
0.2
0.3
0.4
0.5
0.6

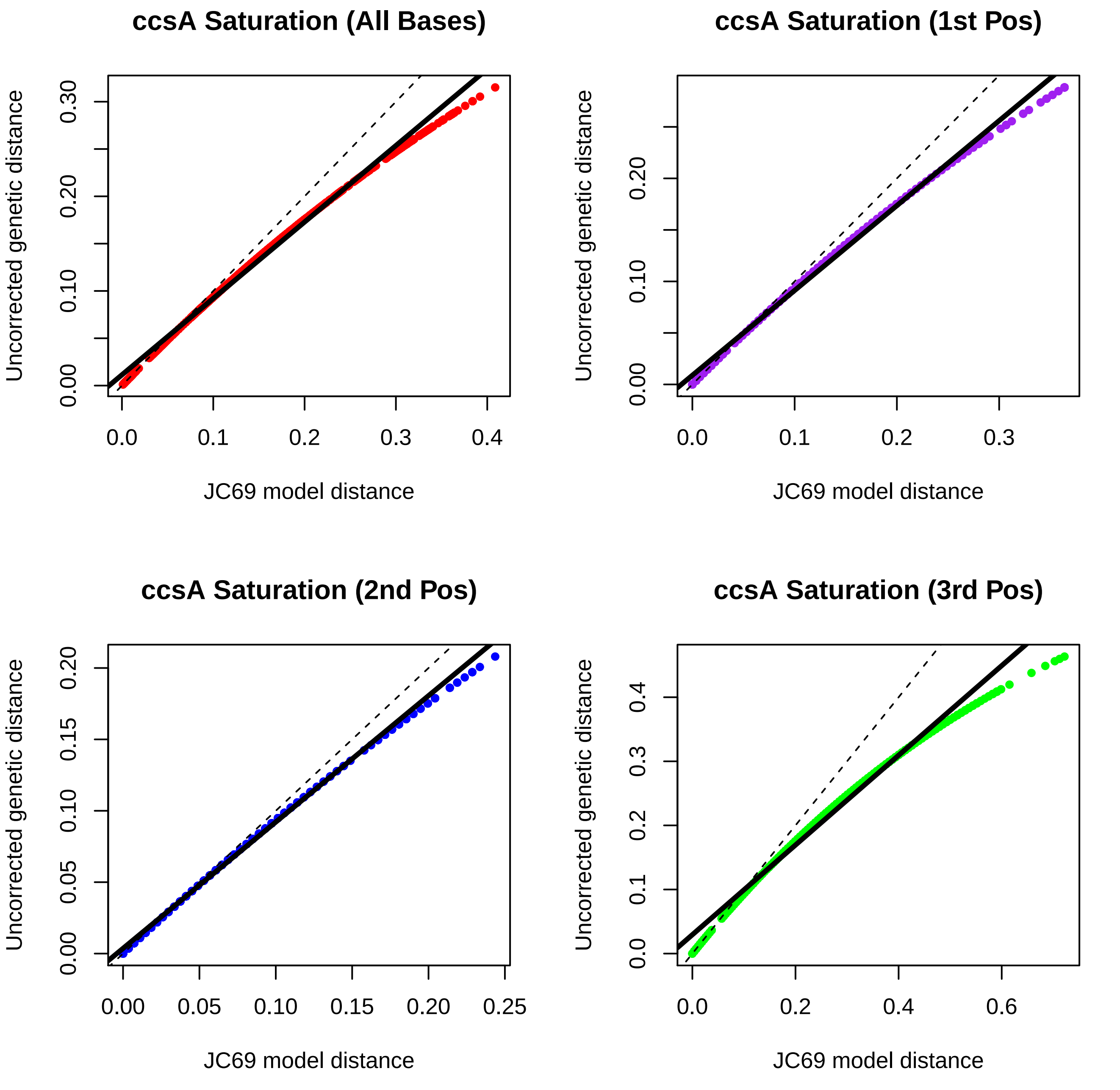
ccsA Saturation (All Bases)
ccsA Saturation (1st Pos)
ccsA Saturation (2nd Pos)
ccsA Saturation (3rd Pos)
Uncorrected genetic distance
JC69 model distance
0.00
0.10
0.20
0.30
0.0
0.1
0.2
0.3
0.4
0.05
0.15
0.25
0.6

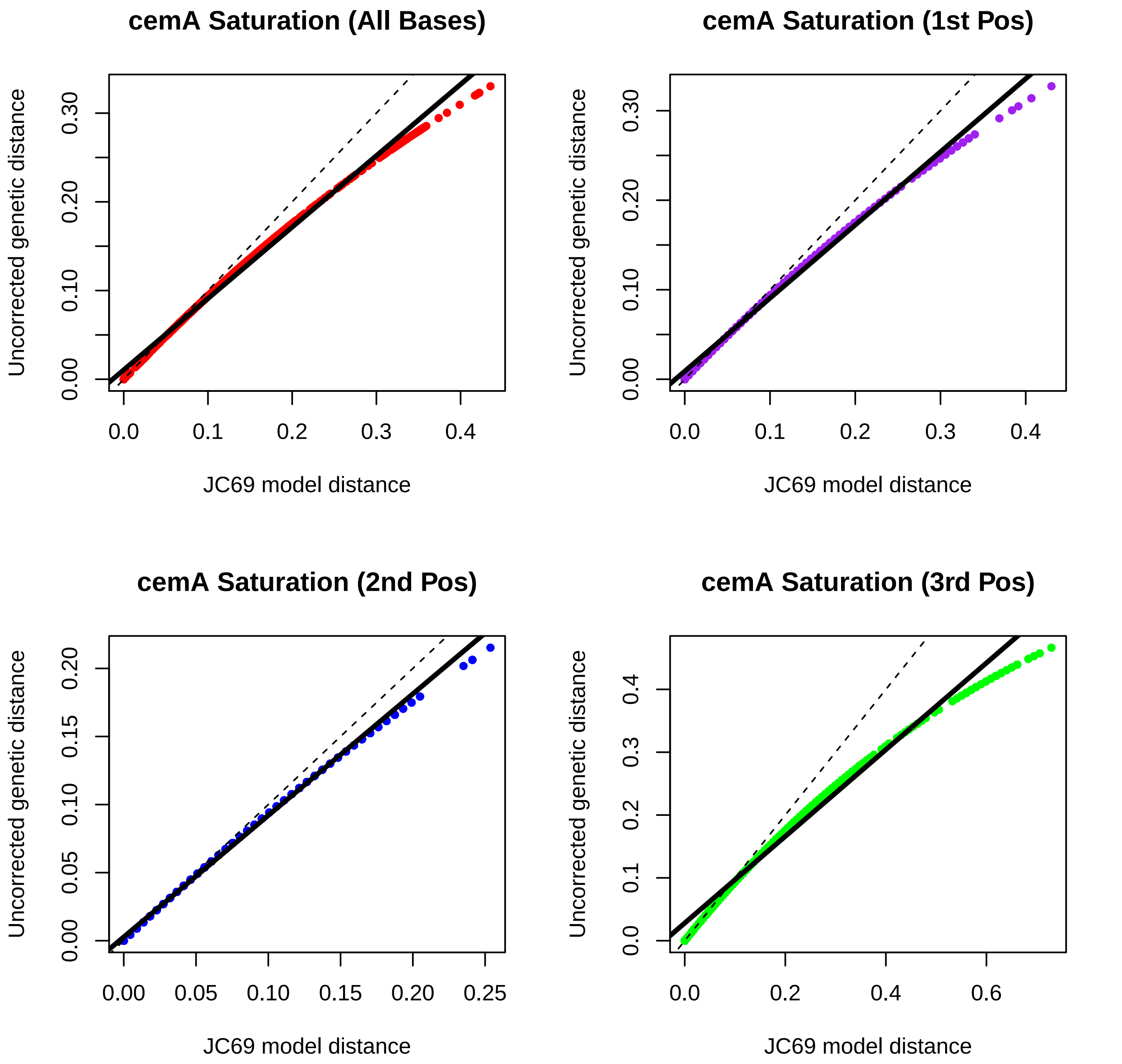

cemA Saturation (All Bases)
cemA Saturation (1st Pos)
cemA Saturation (2nd Pos)
cemA Saturation (3rd Pos)
Uncorrected genetic distance
JC69 model distance
0.00 0.10 0.20 0.30
0.0 0.1 0.2 0.3 0.4
0.00 0.05 0.10 0.15 0.20
0.00 0.05 0.10 0.15 0.20 0.25
0.0 0.1 0.2 0.3 0.4
0.0 0.2 0.4 0.6

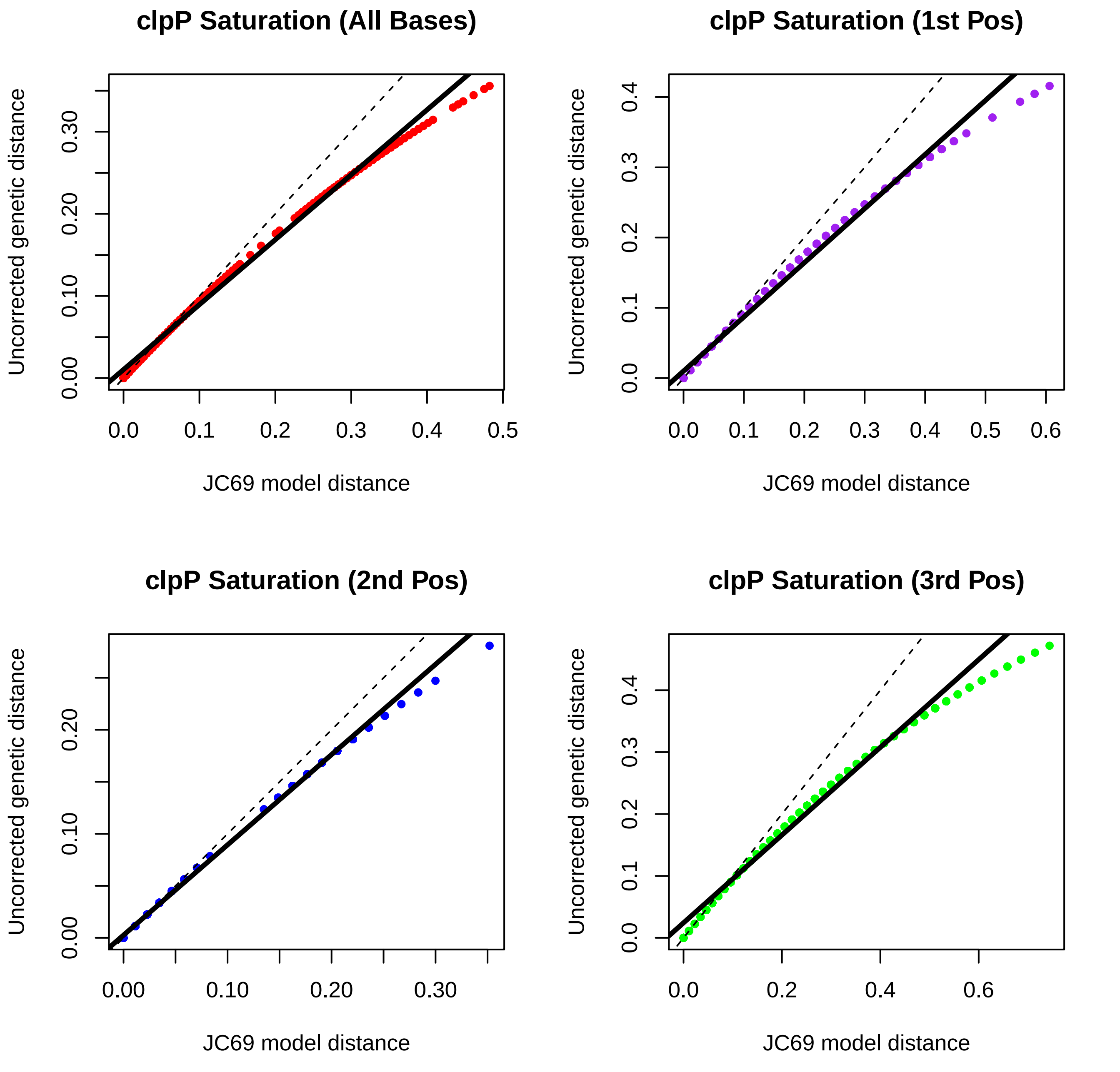

clpP Saturation (All Bases)
clpP Saturation (1st Pos)
clpP Saturation (2nd Pos)
clpP Saturation (3rd Pos)
Uncorrected genetic distance
JC69 model distance

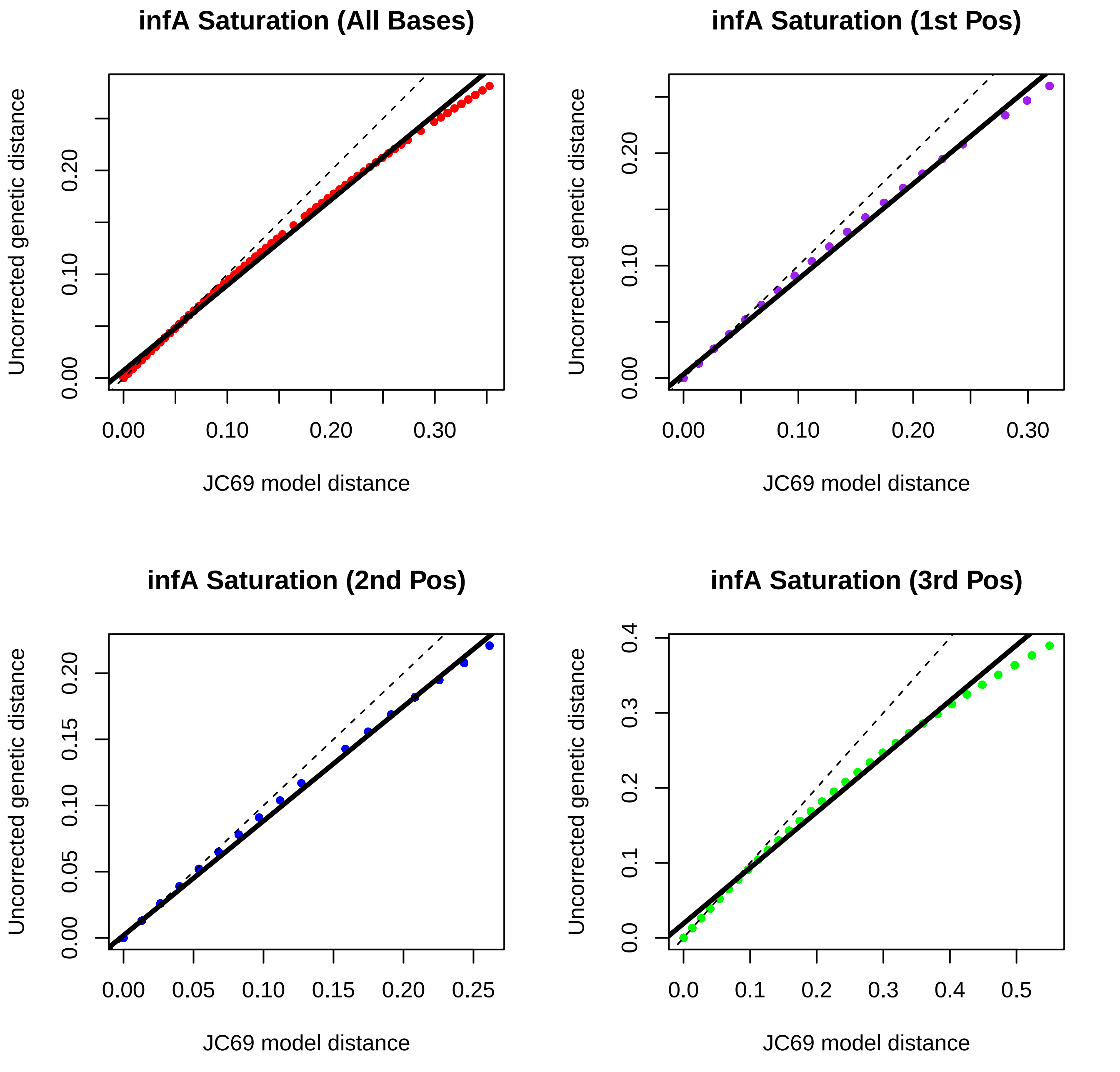

infA Saturation (All Bases)
Uncorrected genetic distance
0.00
0.10
0.20
0.00
0.10
0.20
0.30
JC69 model distance
infA Saturation (1st Pos)
Uncorrected genetic distance
0.00
0.10
0.20
0.00
0.10
0.20
0.30
JC69 model distance
infA Saturation (2nd Pos)
Uncorrected genetic distance
0.00
0.05
0.10
0.15
0.20
0.00
0.05
0.10
0.15
0.20
0.25
JC69 model distance
infA Saturation (3rd Pos)
Uncorrected genetic distance
0.0
0.1
0.2
0.3
0.4
0.0
0.1
0.2
0.3
0.4
0.5
JC69 model distance

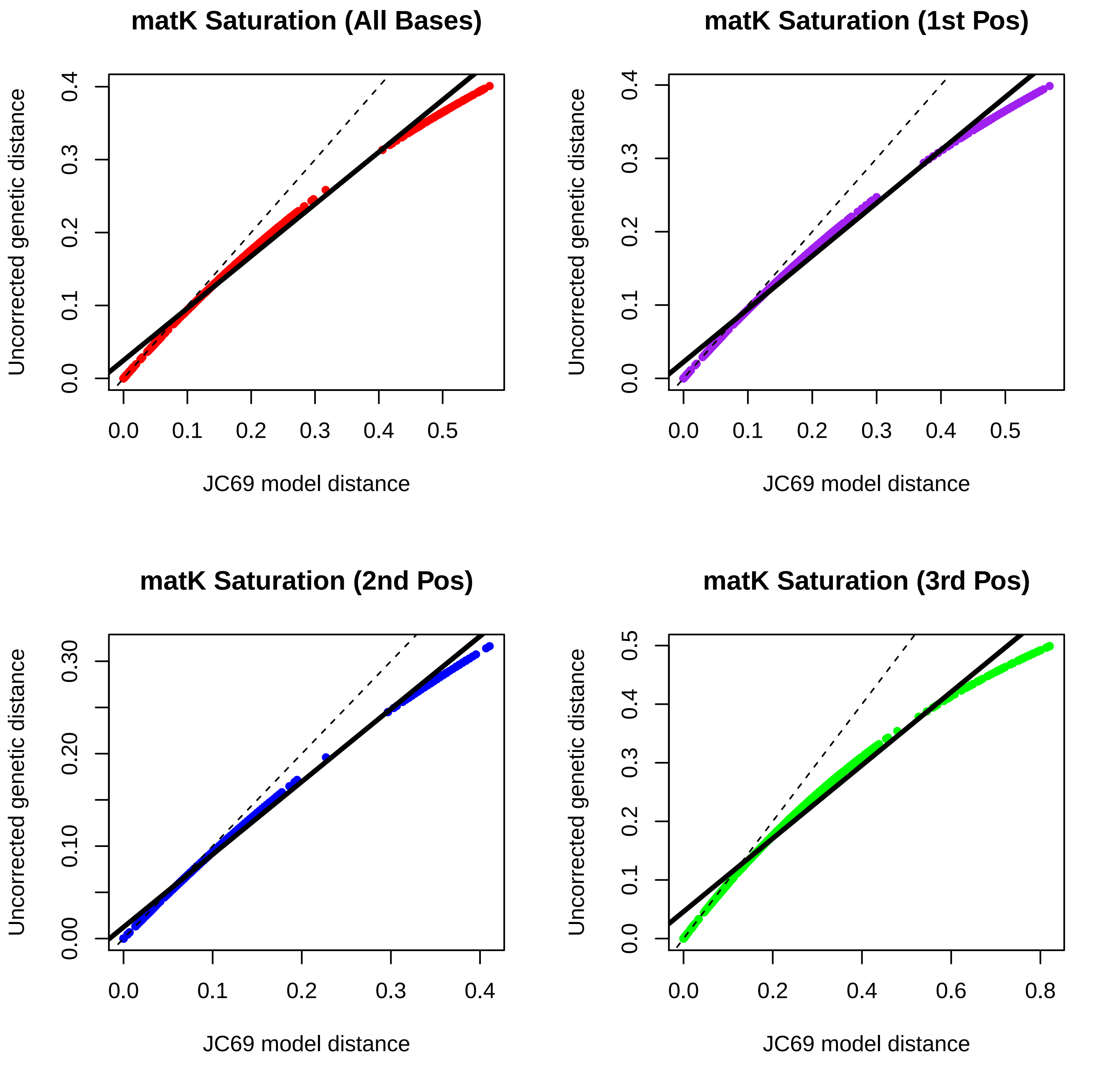

matK Saturation (All Bases)
matK Saturation (1st Pos)
matK Saturation (2nd Pos)
matK Saturation (3rd Pos)
Uncorrected genetic distance
JC69 model distance
0.0
0.1
0.2
0.3
0.4
0.5
0.00
0.10
0.20
0.30
0.6
0.8

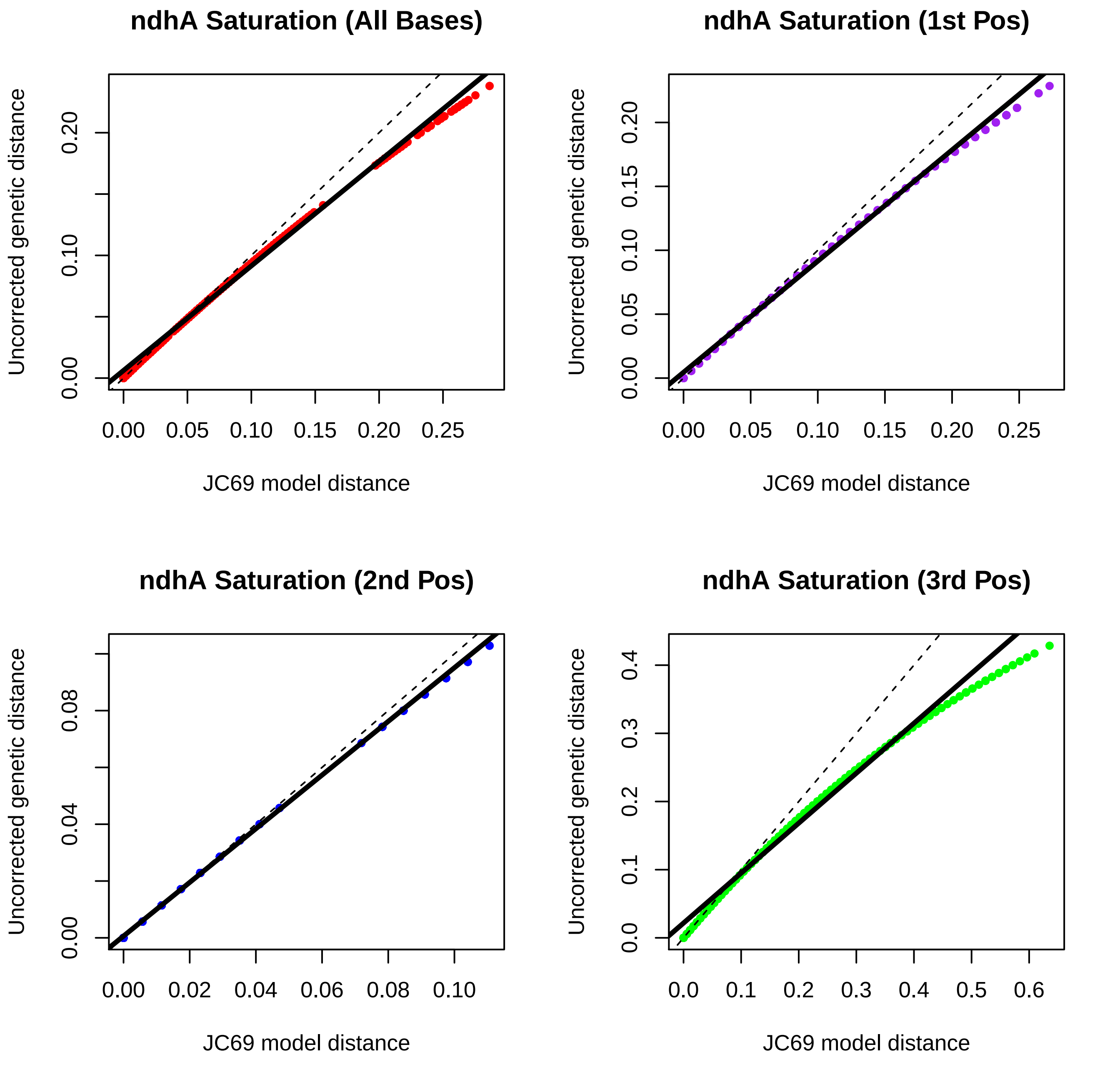

ndhA Saturation (All Bases)
ndhA Saturation (1st Pos)
ndhA Saturation (2nd Pos)
ndhA Saturation (3rd Pos)
Uncorrected genetic distance
JC69 model distance
0.00
0.05
0.10
0.15
0.20
0.25
0.02
0.04
0.06
0.08
0.0
0.1
0.2
0.3
0.4
0.5
0.6

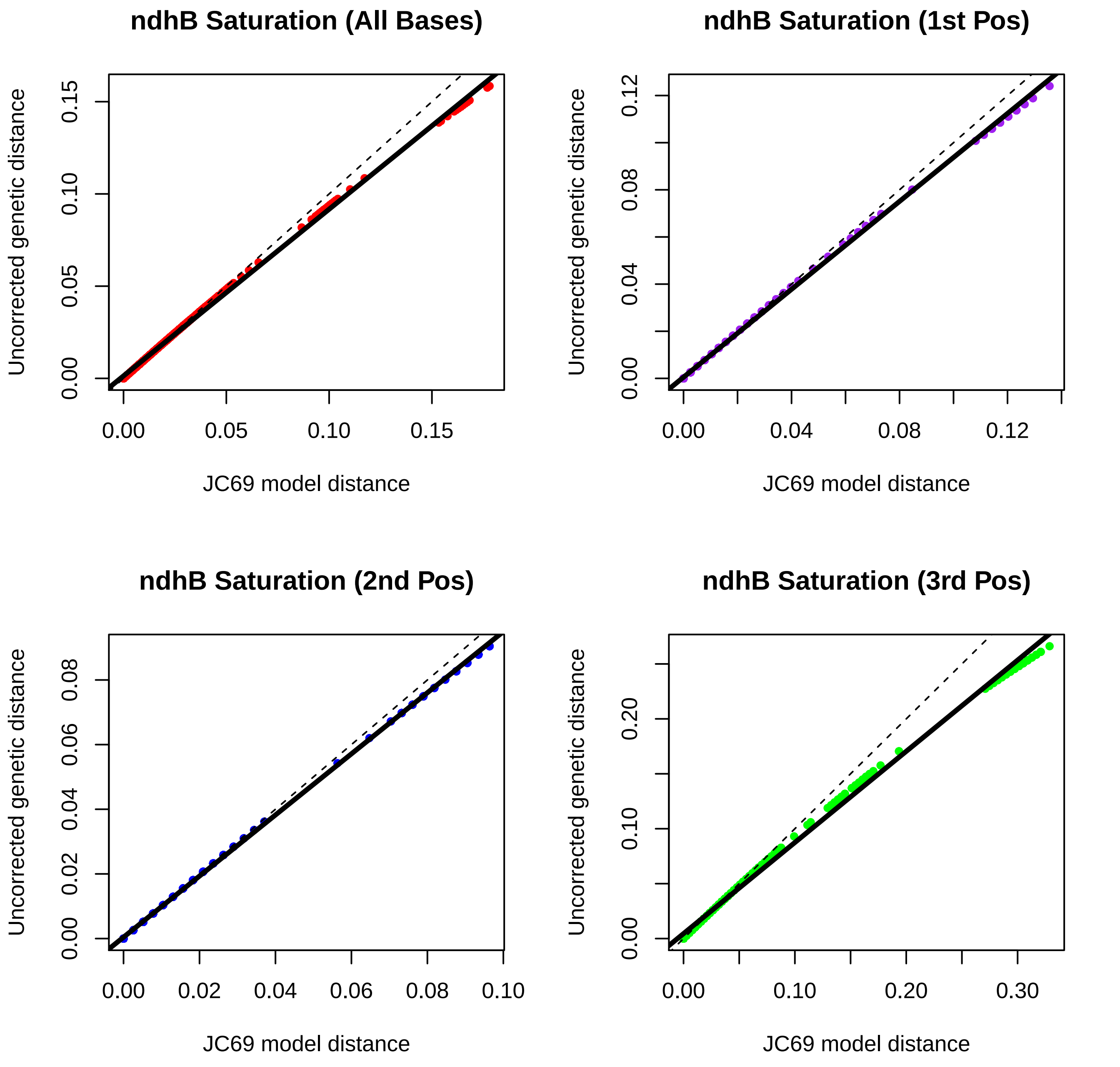

ndhB Saturation (All Bases)
ndhB Saturation (1st Pos)
ndhB Saturation (2nd Pos)
ndhB Saturation (3rd Pos)
Uncorrected genetic distance
JC69 model distance
0.00
0.05
0.10
0.15
0.04
0.08
0.12
0.02
0.06
0.20
0.30

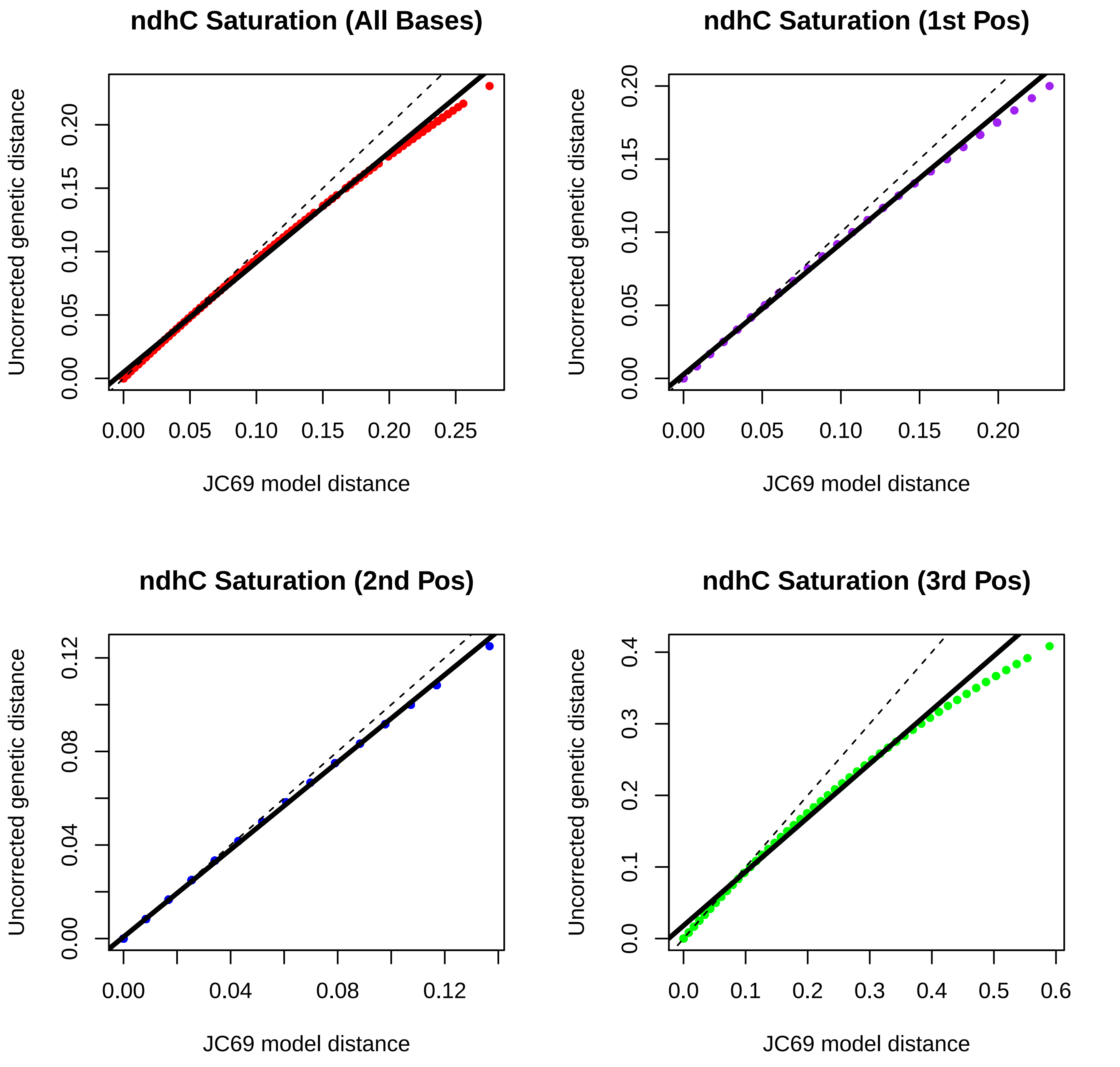

ndhC Saturation (All Bases)
Uncorrected genetic distance
JC69 model distance
0.00
0.05
0.10
0.15
0.20
0.25
ndhC Saturation (1st Pos)
Uncorrected genetic distance
JC69 model distance
ndhC Saturation (2nd Pos)
Uncorrected genetic distance
JC69 model distance
0.00
0.04
0.08
0.12
ndhC Saturation (3rd Pos)
Uncorrected genetic distance
JC69 model distance
0.0
0.1
0.2
0.3
0.4
0.5
0.6

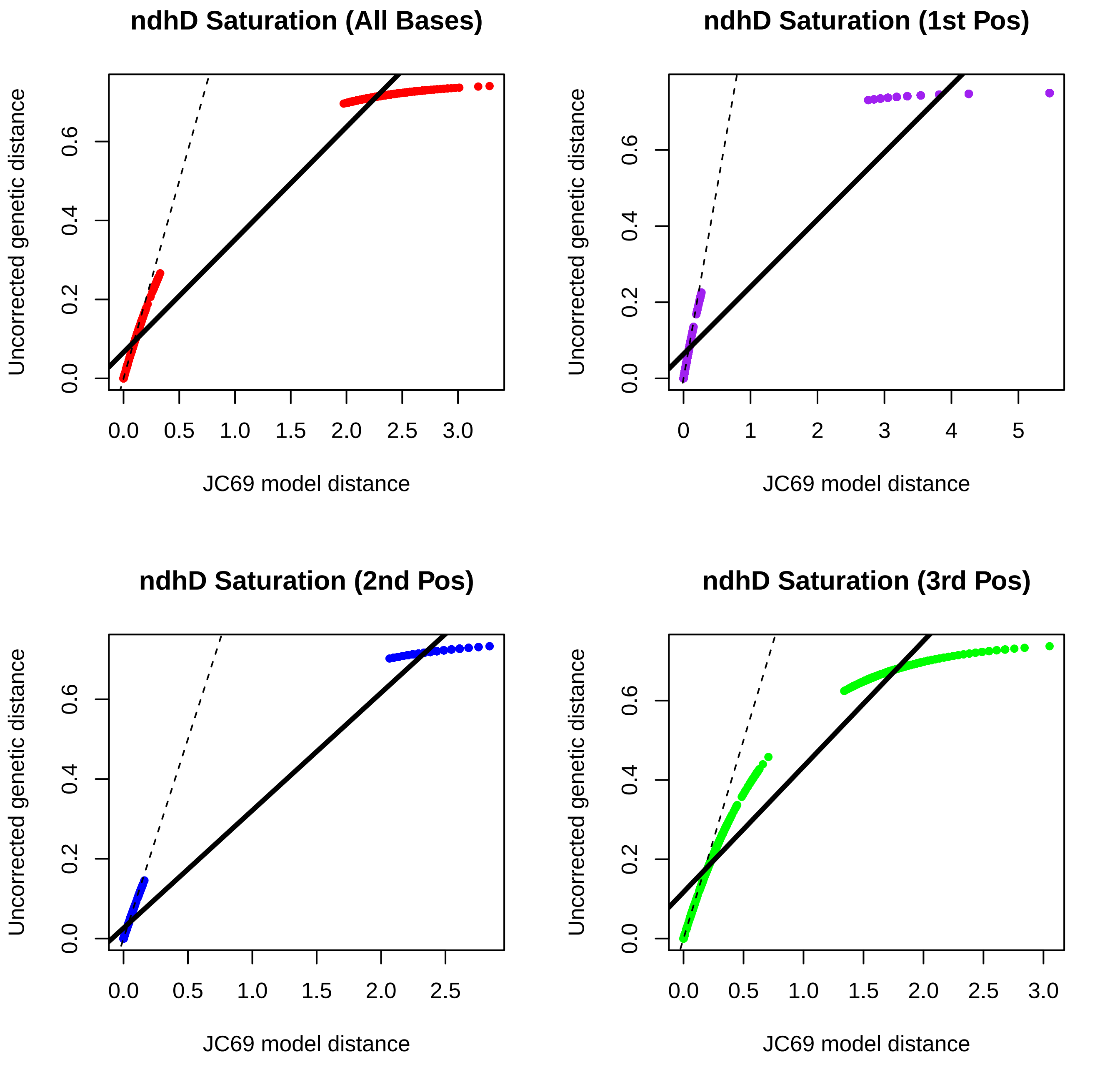

ndhD Saturation (All Bases)
ndhD Saturation (1st Pos)
ndhD Saturation (2nd Pos)
ndhD Saturation (3rd Pos)
Uncorrected genetic distance
JC69 model distance
0.0
0.2
0.4
0.6
0.5
1.0
1.5
2.0
2.5
3.0
0
1
2
3
4
5

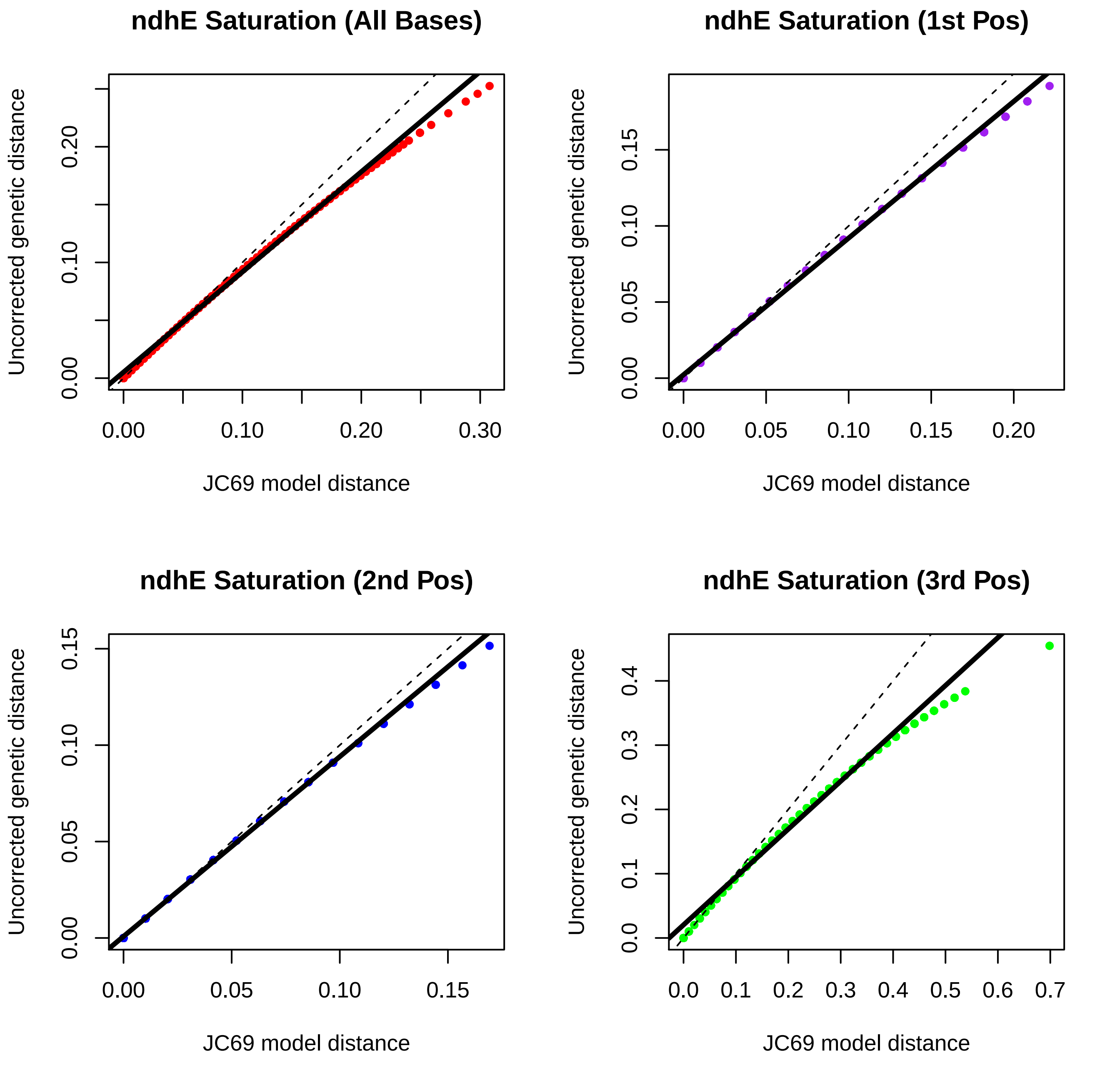
ndhE Saturation (All Bases)
ndhE Saturation (1st Pos)
ndhE Saturation (2nd Pos)
ndhE Saturation (3rd Pos)
Uncorrected genetic distance
JC69 model distance

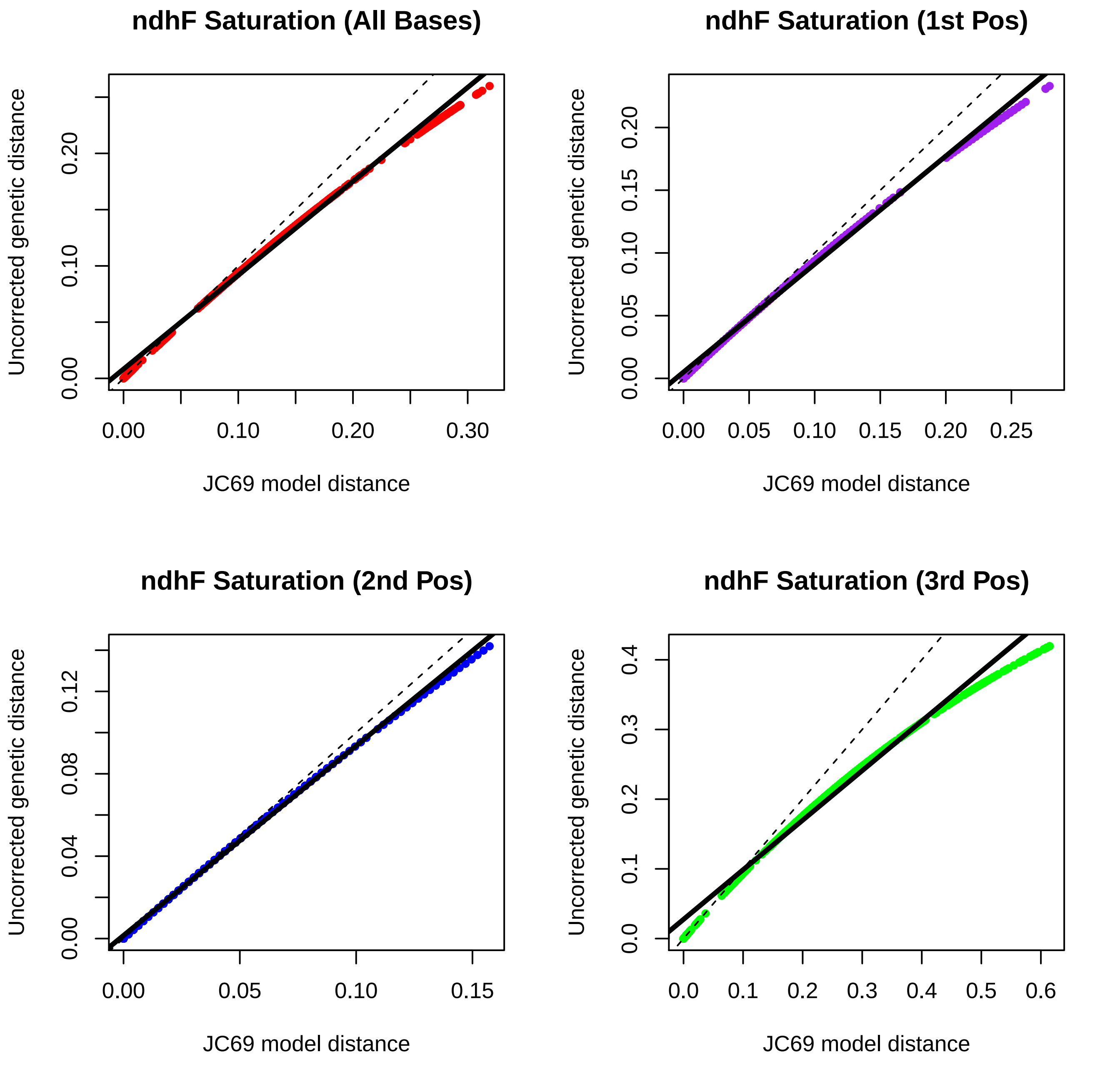

ndhF Saturation (All Bases)
ndhF Saturation (1st Pos)
ndhF Saturation (2nd Pos)
ndhF Saturation (3rd Pos)
Uncorrected genetic distance
JC69 model distance
0.00
0.10
0.20
0.30
0.05
0.15
0.25
0.04
0.08
0.12
0.0
0.1
0.2
0.3
0.4
0.5
0.6

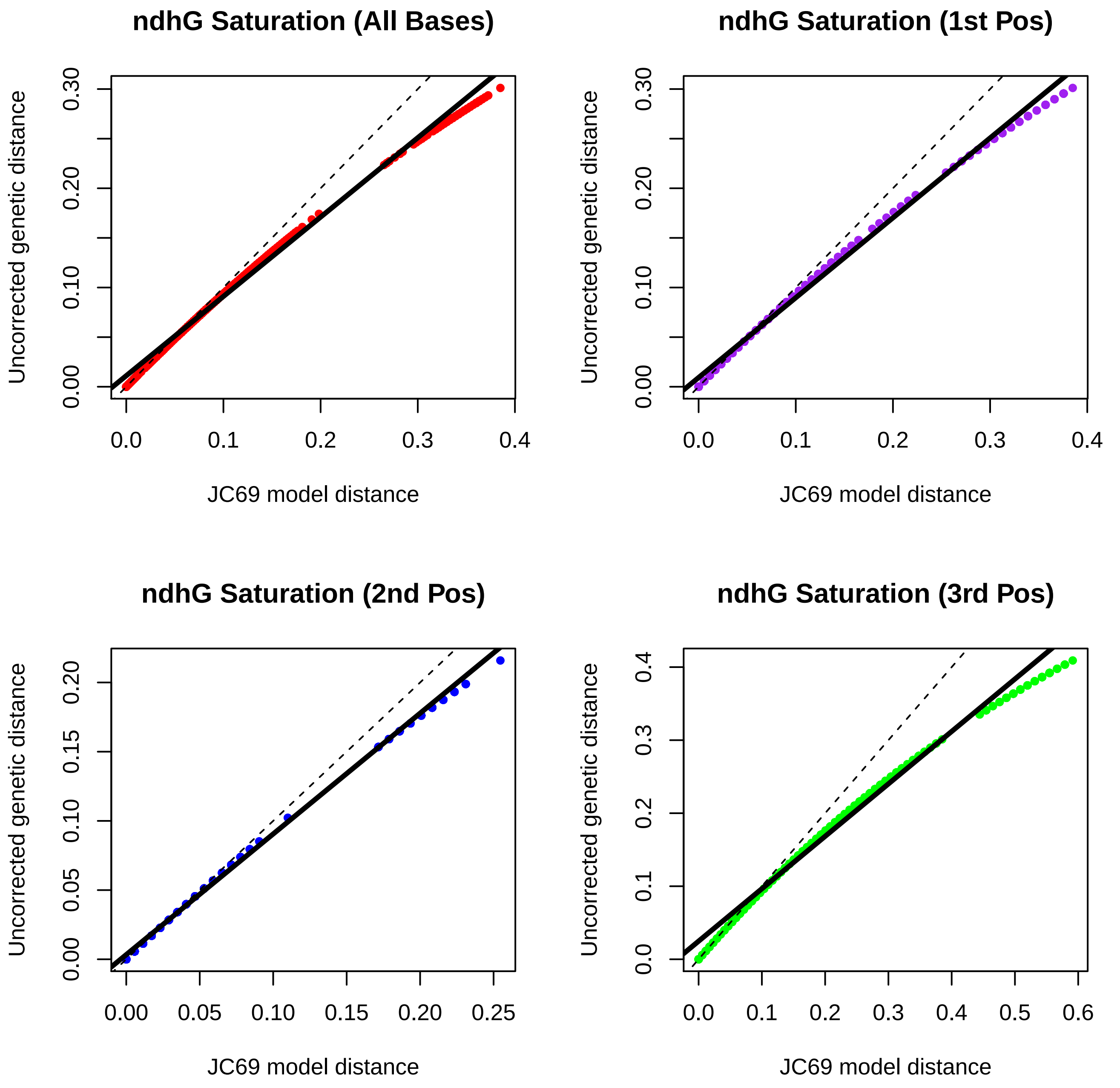
ndhG Saturation (All Bases)
ndhG Saturation (1st Pos)
ndhG Saturation (2nd Pos)
ndhG Saturation (3rd Pos)
Uncorrected genetic distance
JC69 model distance
0.00
0.10
0.20
0.30
0.0
0.1
0.2
0.3
0.4
0.05
0.15
0.25
0.5
0.6

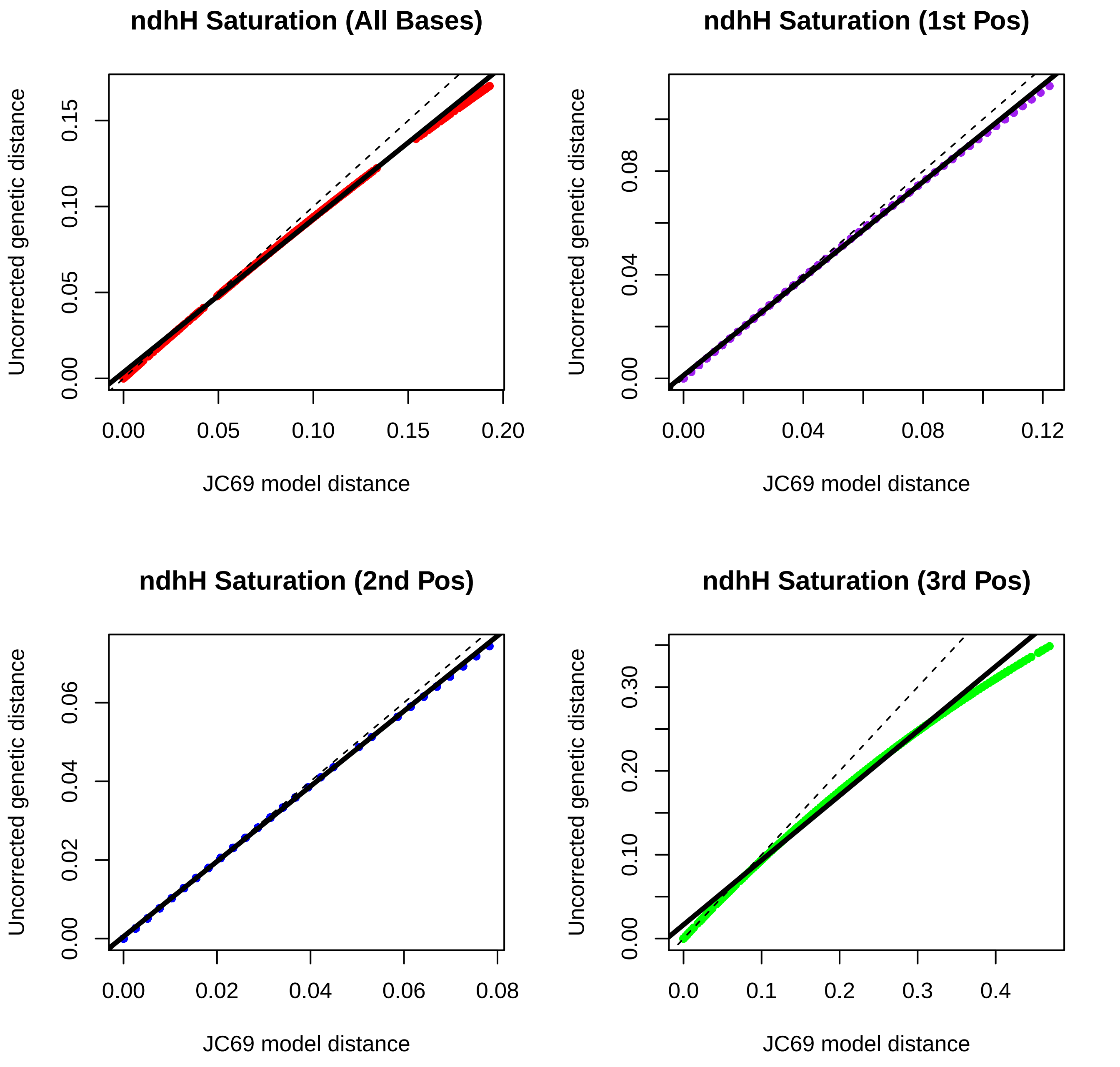
ndhH Saturation (All Bases)
Uncorrected genetic distance
JC69 model distance
0.00
0.05
0.10
0.15
0.20
ndhH Saturation (1st Pos)
0.00
0.04
0.08
0.12
ndhH Saturation (2nd Pos)
0.00
0.02
0.04
0.06
0.08
ndhH Saturation (3rd Pos)
0.0
0.1
0.2
0.3
0.4
0.00
0.10
0.20
0.30

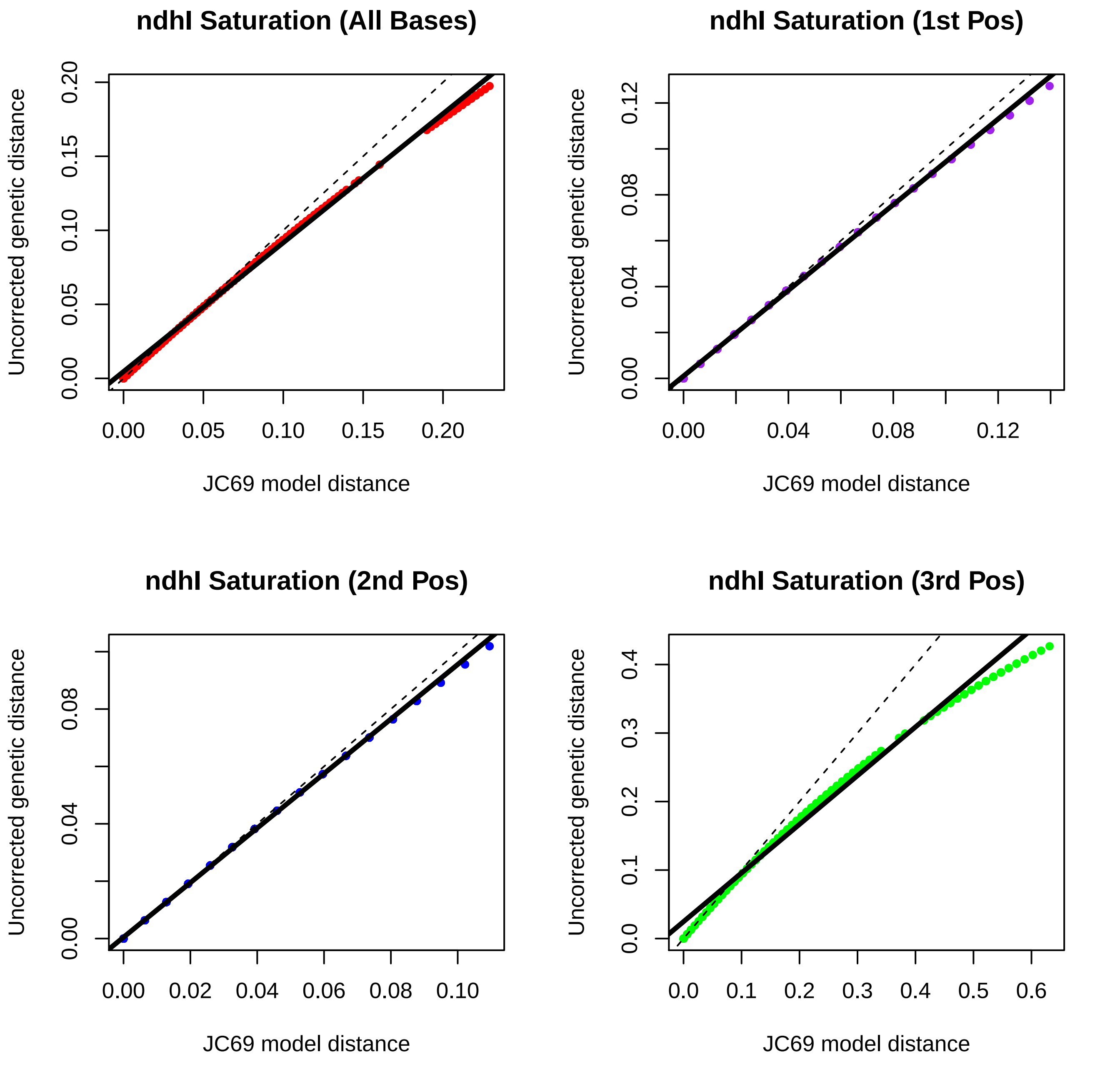

ndhI Saturation (All Bases)
ndhI Saturation (1st Pos)
ndhI Saturation (2nd Pos)
ndhI Saturation (3rd Pos)
Uncorrected genetic distance
JC69 model distance
0.00
0.05
0.10
0.15
0.20
0.04
0.08
0.12
0.02
0.06
0.0
0.1
0.2
0.3
0.4
0.5
0.6

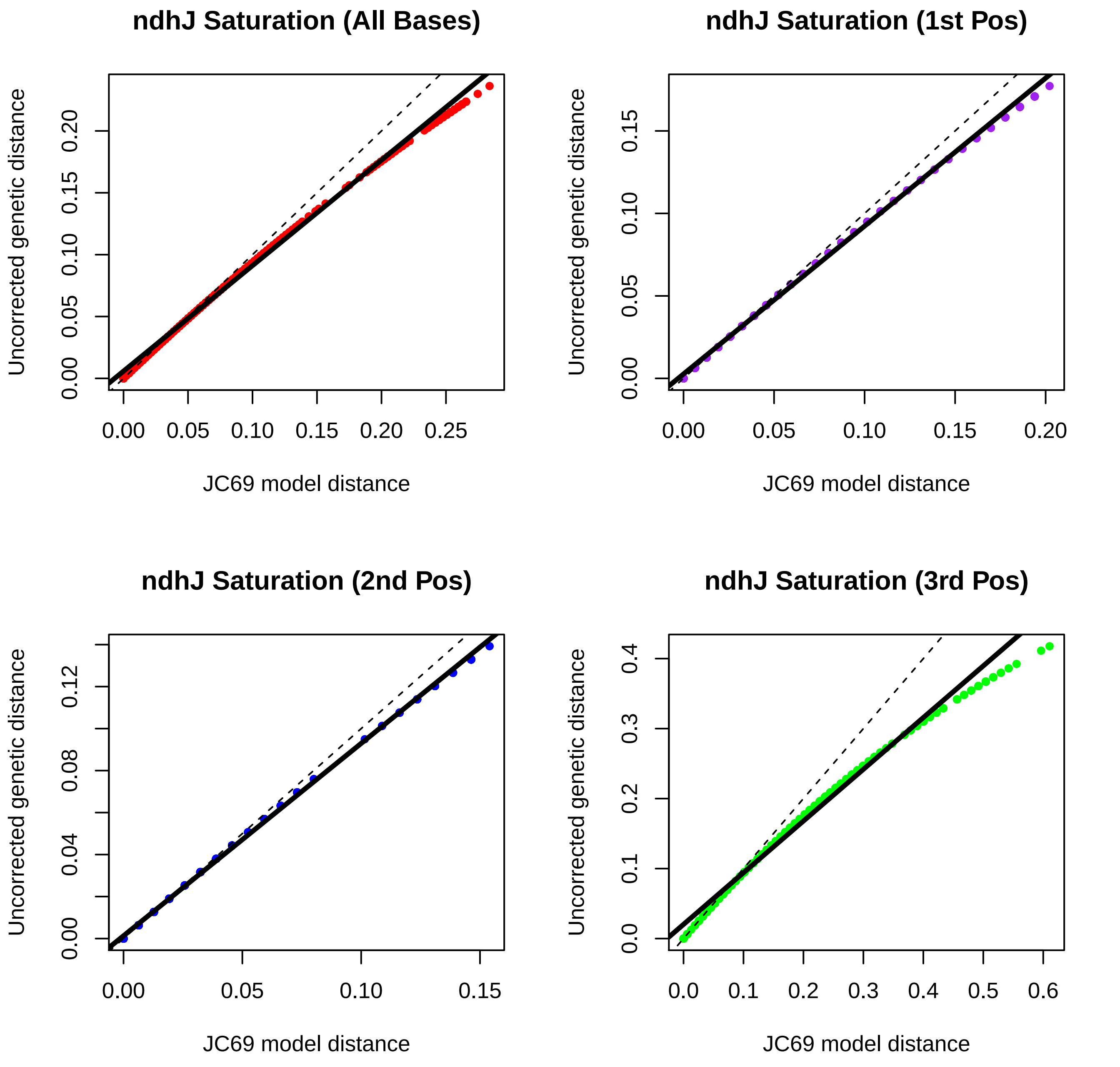
ndhJ Saturation (All Bases)
ndhJ Saturation (1st Pos)
ndhJ Saturation (2nd Pos)
ndhJ Saturation (3rd Pos)
Uncorrected genetic distance
JC69 model distance
0.00
0.05
0.10
0.15
0.20
0.25
0.04
0.08
0.12
0.0
0.1
0.2
0.3
0.4
0.5
0.6

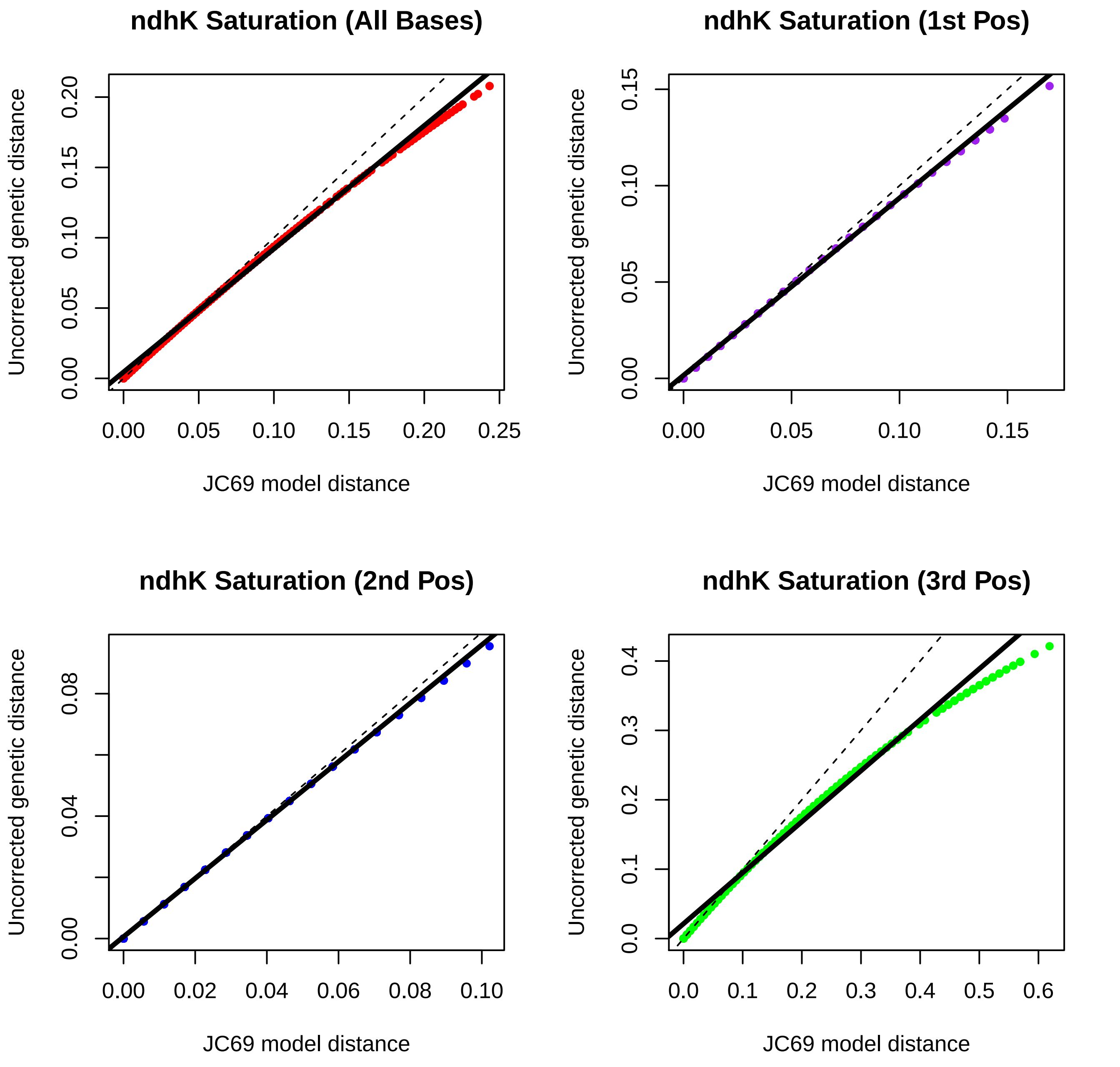

ndhK Saturation (All Bases)
ndhK Saturation (1st Pos)
ndhK Saturation (2nd Pos)
ndhK Saturation (3rd Pos)
Uncorrected genetic distance
JC69 model distance
0.00
0.05
0.10
0.15
0.20
0.25
0.02
0.04
0.06
0.08
0.0
0.1
0.2
0.3
0.4
0.5
0.6

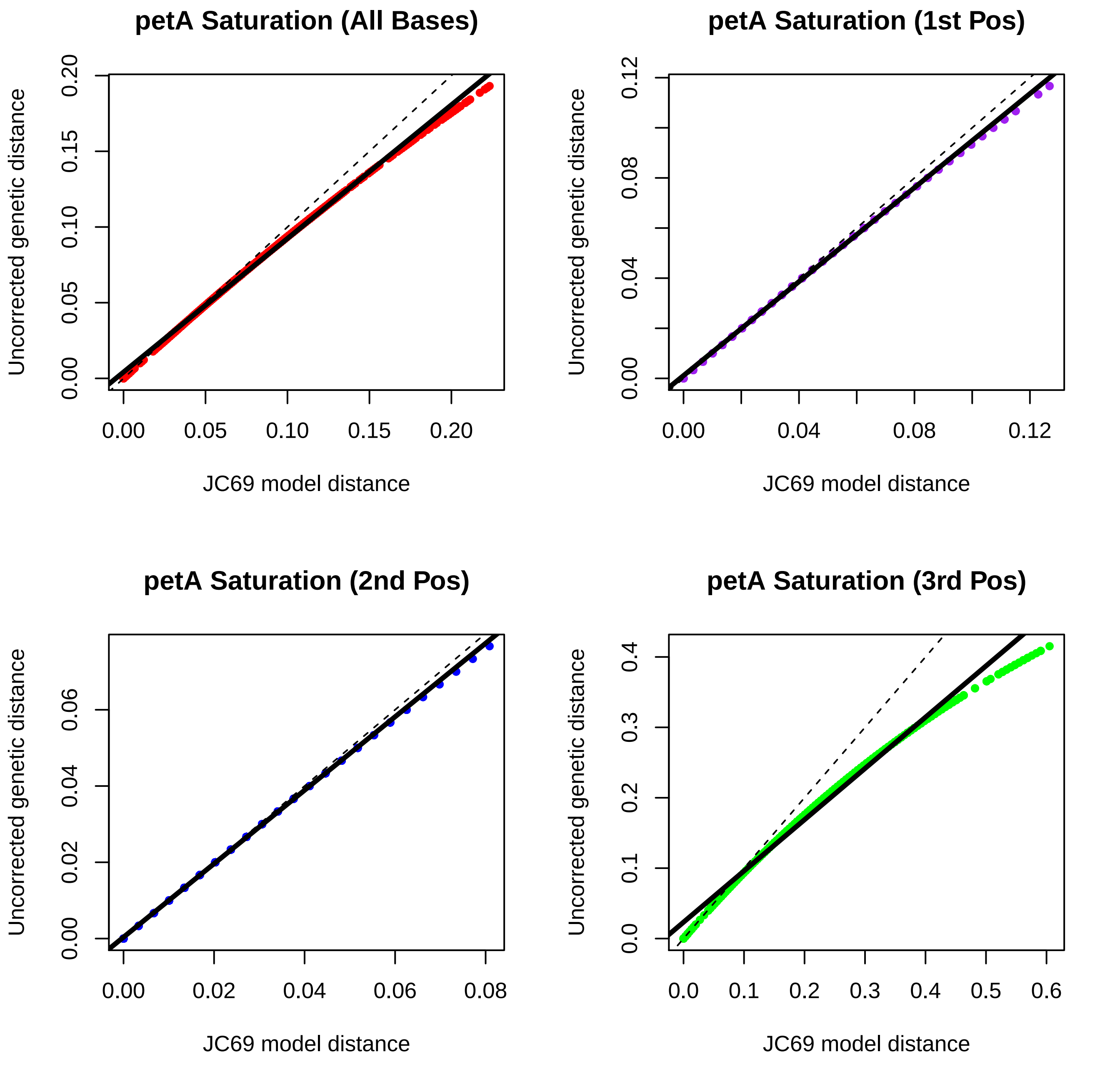

petA Saturation (All Bases)
petA Saturation (1st Pos)
petA Saturation (2nd Pos)
petA Saturation (3rd Pos)
Uncorrected genetic distance
JC69 model distance
0.00
0.05
0.10
0.15
0.20
0.04
0.08
0.12
0.02
0.06
0.0
0.1
0.2
0.3
0.4
0.5
0.6

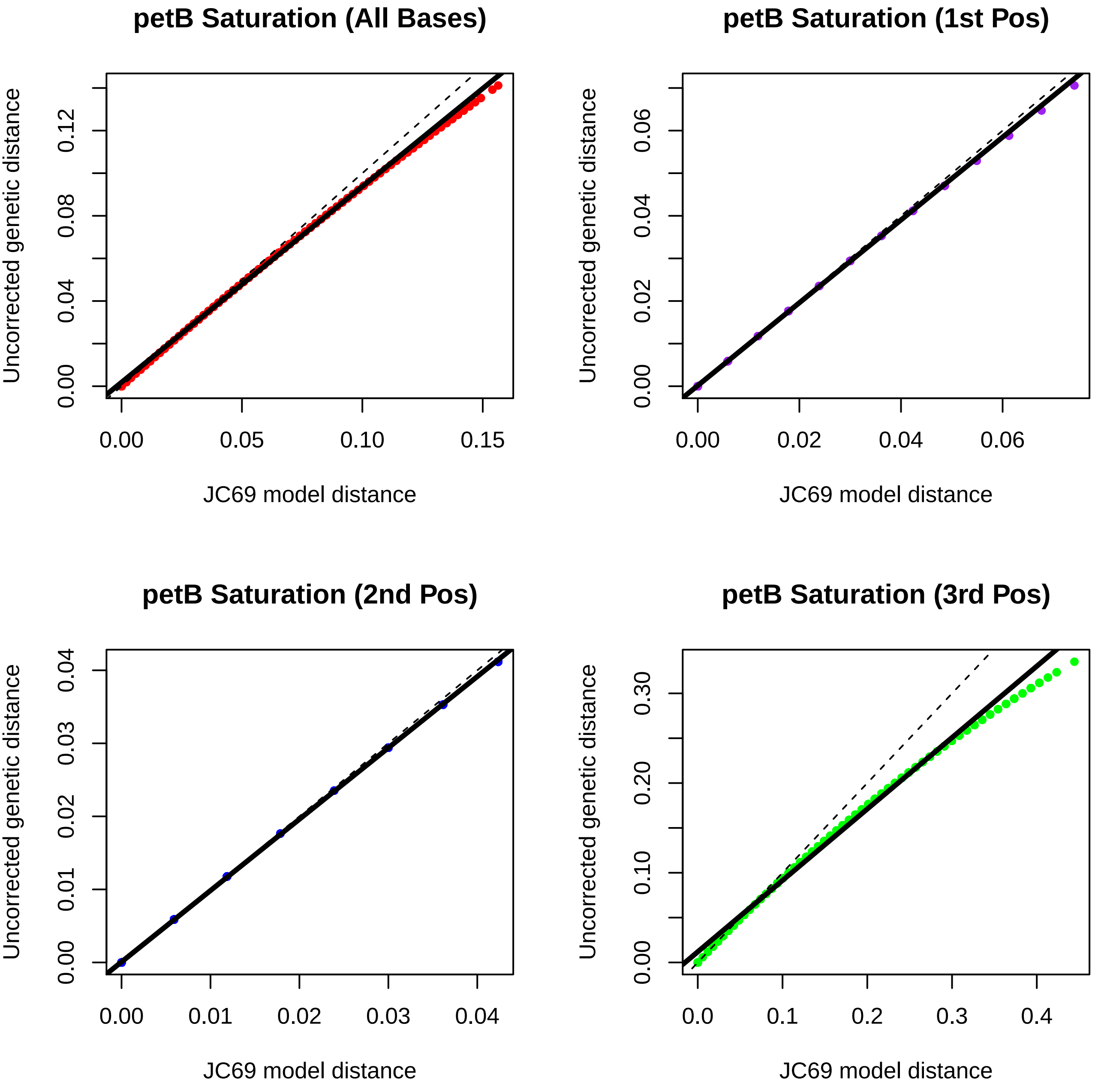
petB Saturation (All Bases)
Uncorrected genetic distance
0.00
0.04
0.08
0.12
0.00
0.05
0.10
0.15
JC69 model distance
petB Saturation (1st Pos)
Uncorrected genetic distance
0.00
0.02
0.04
0.06
0.00
0.02
0.04
0.06
JC69 model distance
petB Saturation (2nd Pos)
Uncorrected genetic distance
0.00
0.01
0.02
0.03
0.04
0.00
0.01
0.02
0.03
0.04
JC69 model distance
petB Saturation (3rd Pos)
Uncorrected genetic distance
0.00
0.10
0.20
0.30
0.0
0.1
0.2
0.3
0.4
JC69 model distance

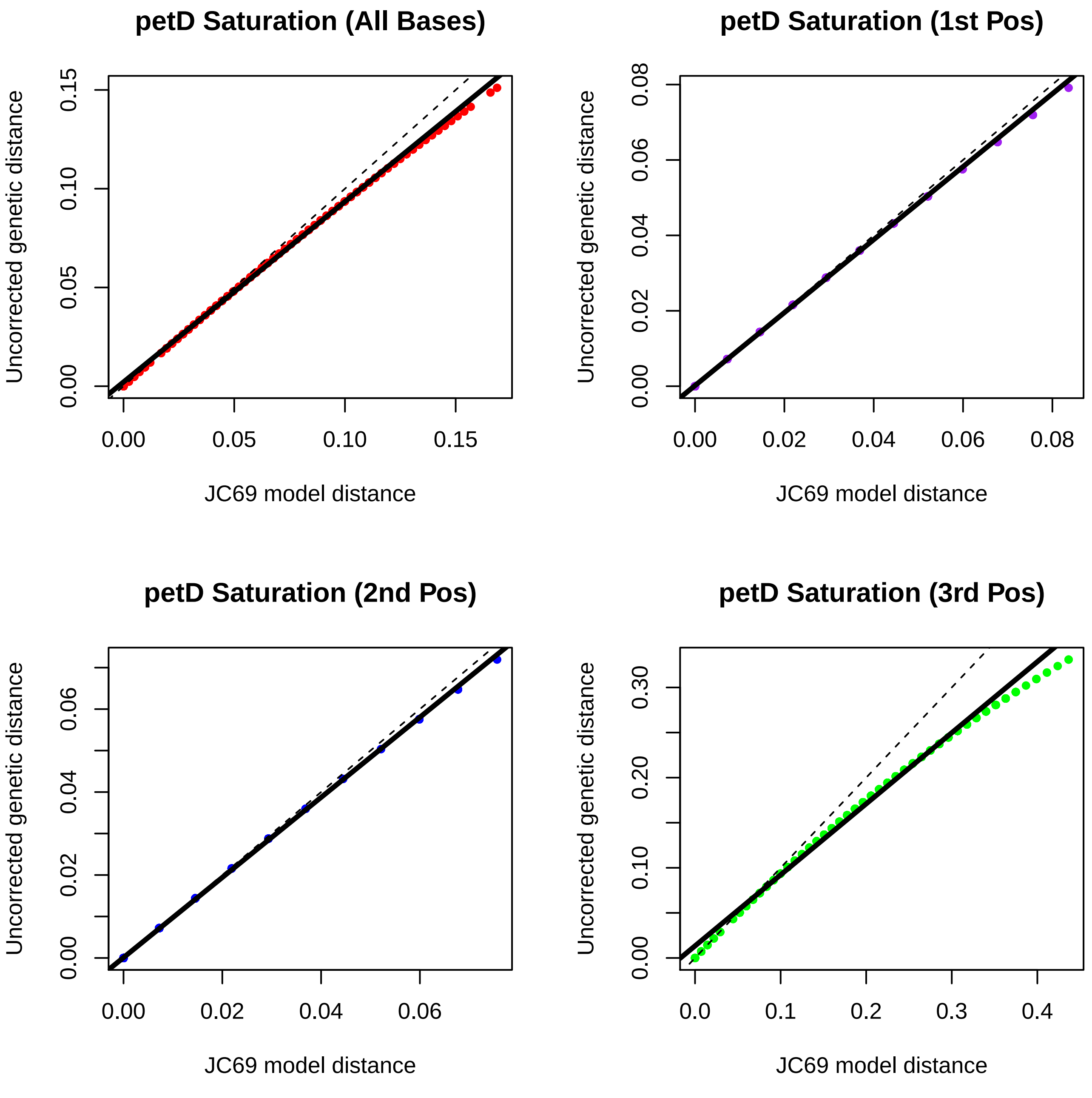
petD Saturation (All Bases)
petD Saturation (1st Pos)
petD Saturation (2nd Pos)
petD Saturation (3rd Pos)
Uncorrected genetic distance
JC69 model distance
0.00
0.05
0.10
0.15
0.00
0.02
0.04
0.06
0.08
0.0
0.1
0.2
0.3
0.4
0.10
0.20
0.30

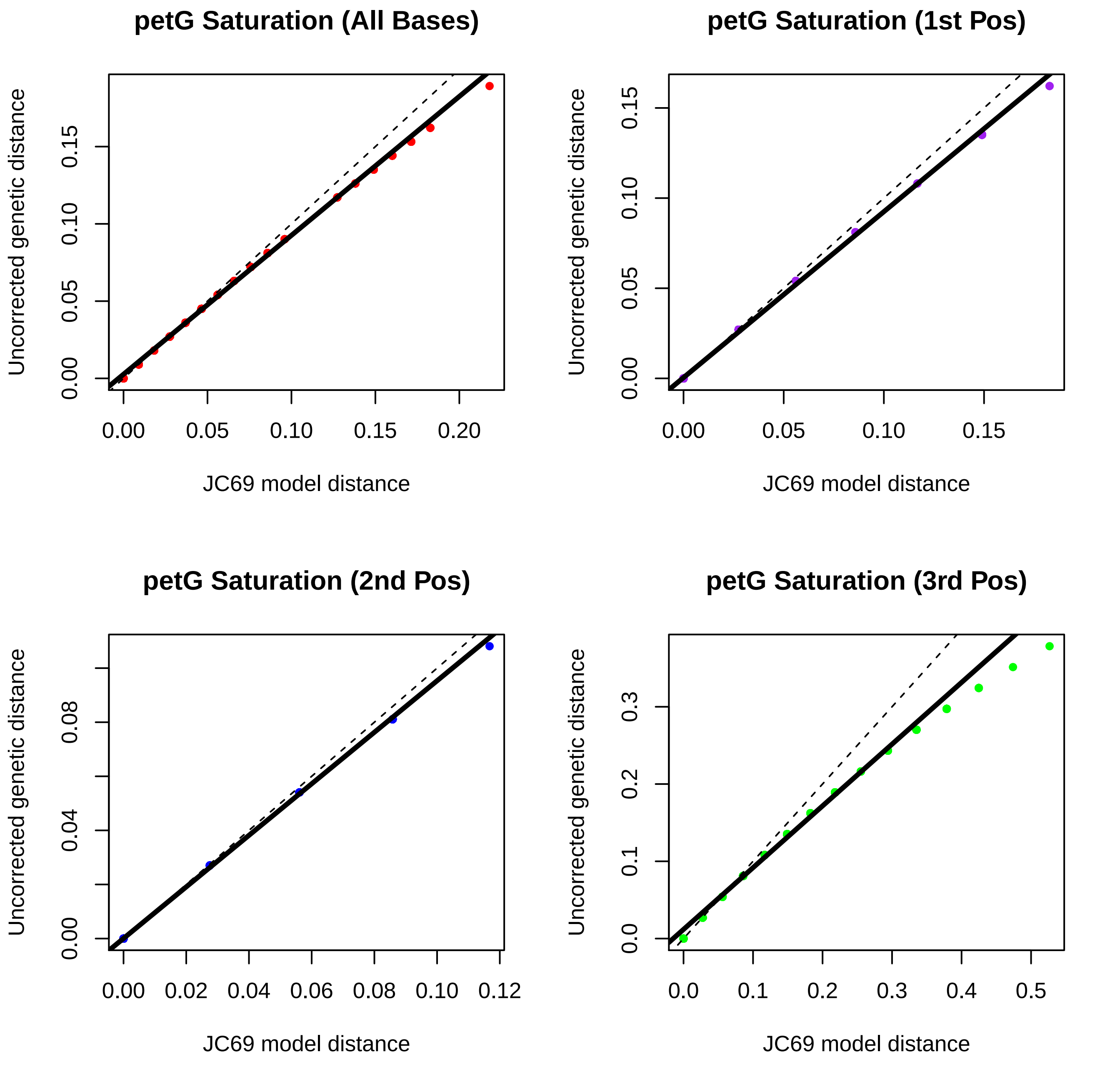
petG Saturation (All Bases)
petG Saturation (1st Pos)
petG Saturation (2nd Pos)
petG Saturation (3rd Pos)
Uncorrected genetic distance
JC69 model distance
0.00
0.05
0.10
0.15
0.20
0.02
0.04
0.06
0.08
0.12
0.0
0.1
0.2
0.3
0.4
0.5

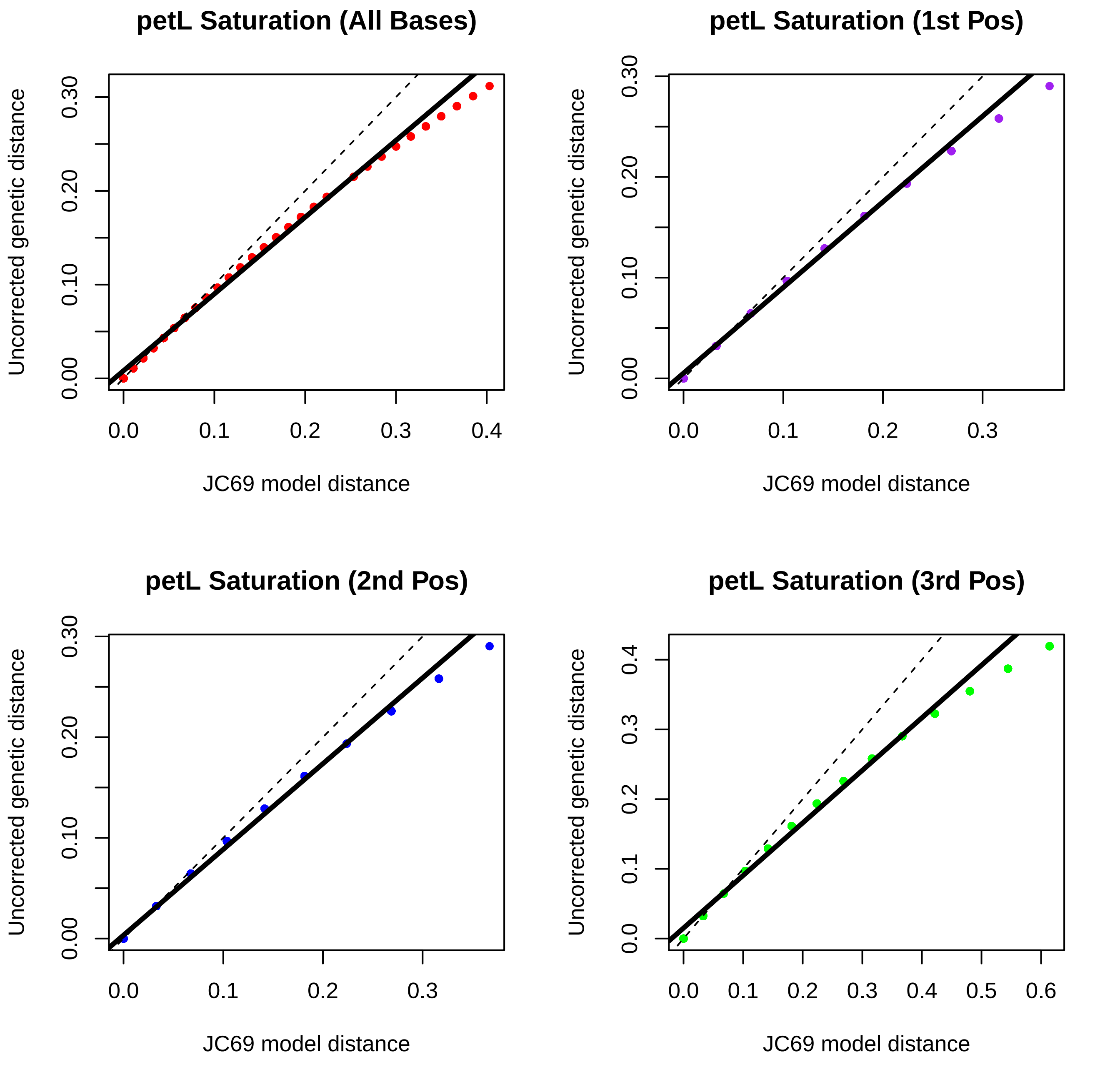

petL Saturation (All Bases)
petL Saturation (1st Pos)
petL Saturation (2nd Pos)
petL Saturation (3rd Pos)
Uncorrected genetic distance
JC69 model distance

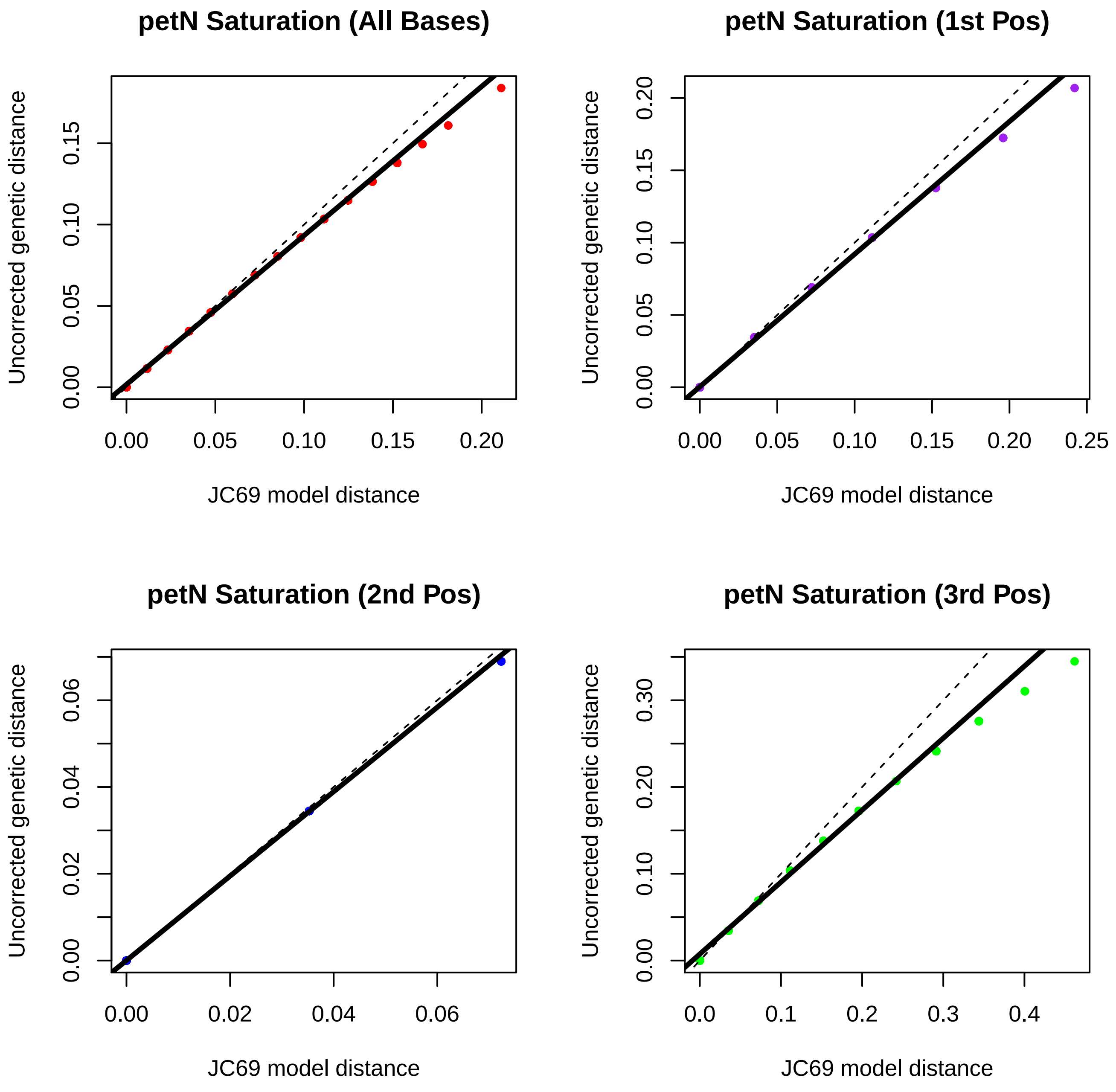

petN Saturation (All Bases)
Uncorrected genetic distance
0.00
0.05
0.10
0.15
0.00
0.05
0.10
0.15
0.20
JC69 model distance
petN Saturation (1st Pos)
Uncorrected genetic distance
0.00
0.05
0.10
0.15
0.20
0.00
0.05
0.10
0.15
0.20
0.25
JC69 model distance
petN Saturation (2nd Pos)
Uncorrected genetic distance
0.00
0.02
0.04
0.06
0.00
0.02
0.04
0.06
JC69 model distance
petN Saturation (3rd Pos)
Uncorrected genetic distance
0.00
0.10
0.20
0.30
0.0
0.1
0.2
0.3
0.4
JC69 model distance

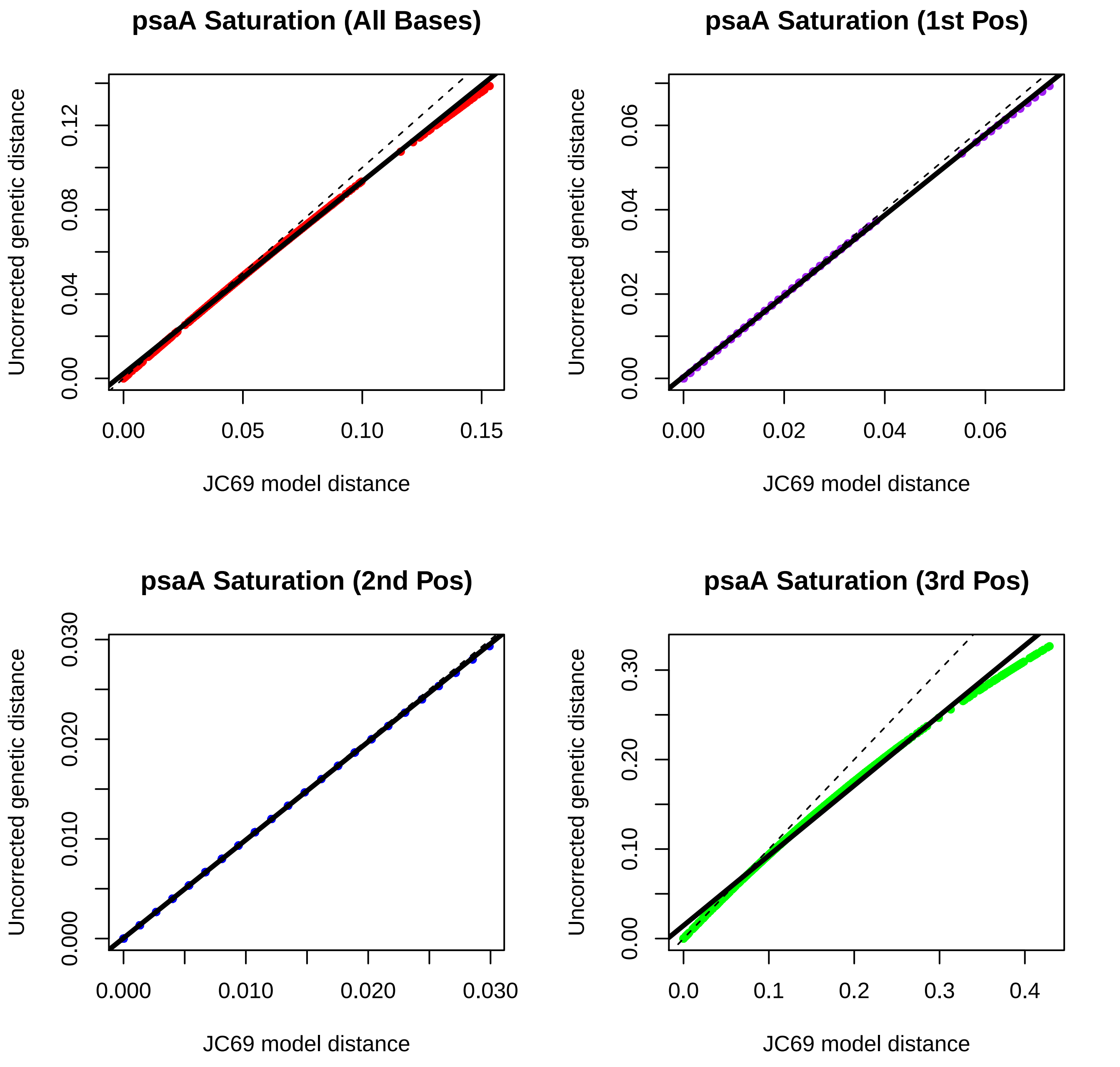
psaA Saturation (All Bases)
psaA Saturation (1st Pos)
psaA Saturation (2nd Pos)
psaA Saturation (3rd Pos)
Uncorrected genetic distance
JC69 model distance
0.00
0.04
0.08
0.12
0.05
0.10
0.15
0.02
0.06
0.000
0.010
0.020
0.030
0.10
0.20
0.30
0.0
0.1
0.2
0.3
0.4

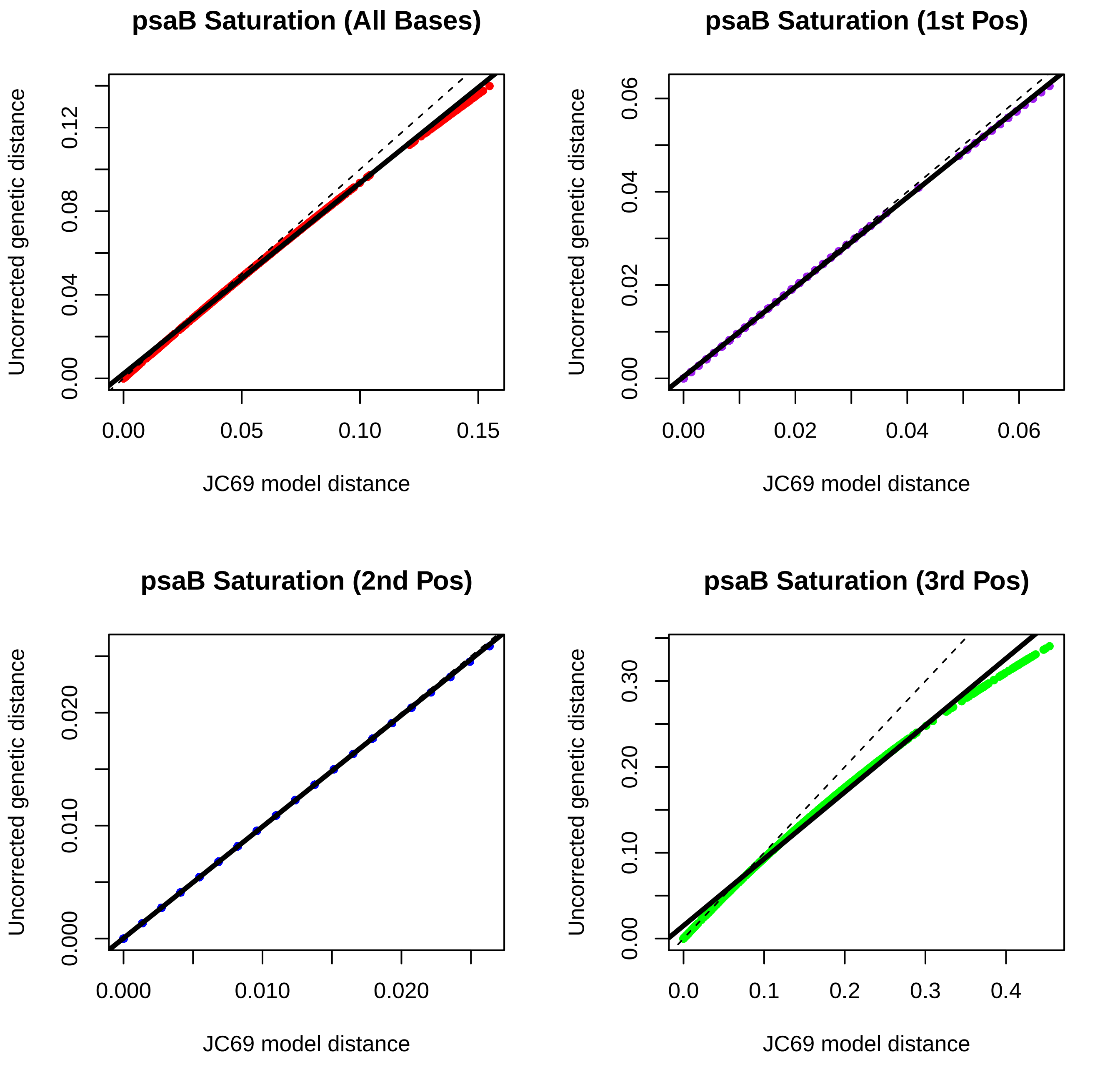
psaB Saturation (All Bases)
psaB Saturation (1st Pos)
psaB Saturation (2nd Pos)
psaB Saturation (3rd Pos)
Uncorrected genetic distance
JC69 model distance
0.00
0.04
0.08
0.12
0.05
0.10
0.15
0.02
0.06
0.000
0.010
0.020
0.10
0.20
0.30
0.0
0.1
0.2
0.3
0.4

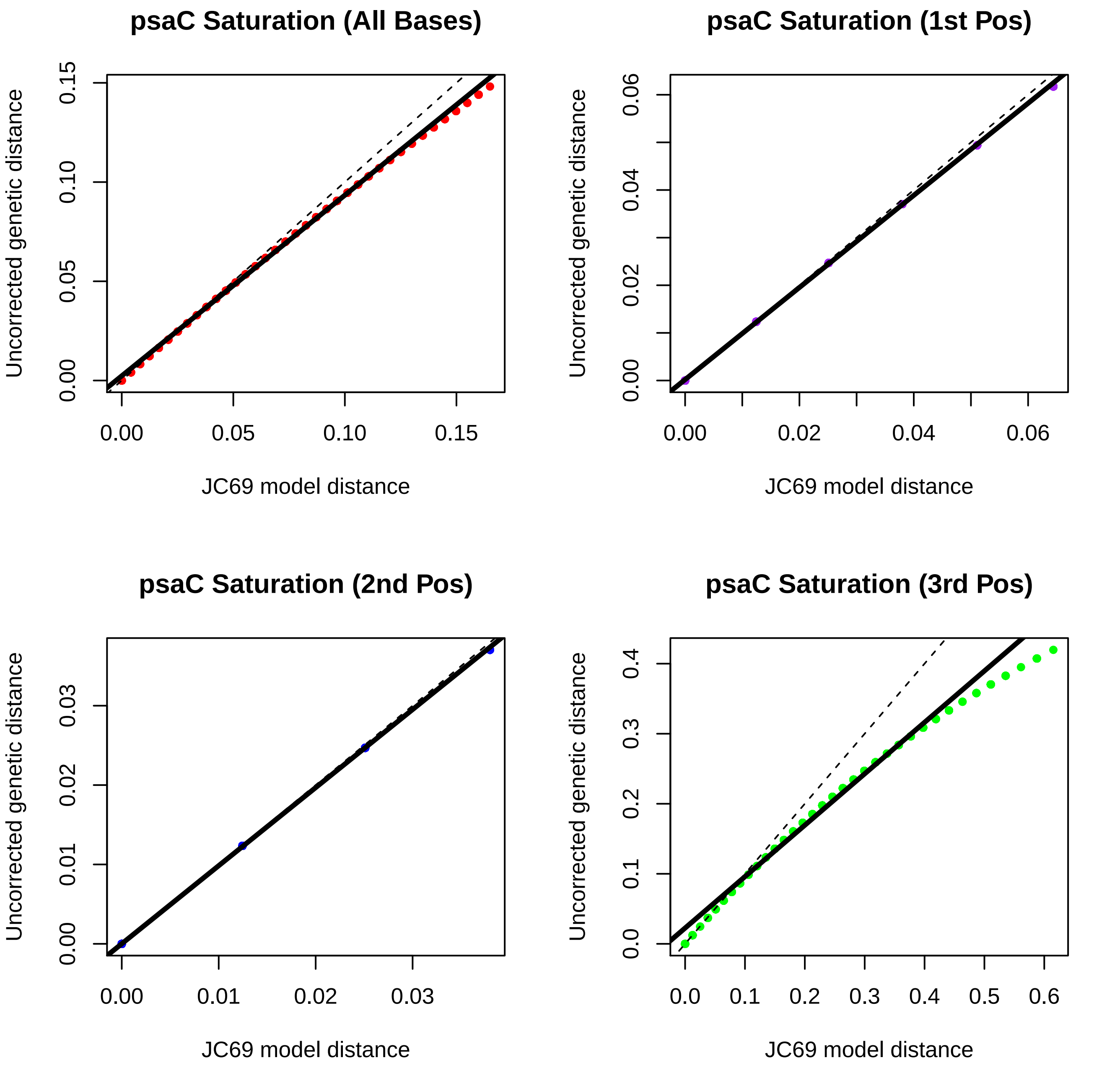
psaC Saturation (All Bases)
Uncorrected genetic distance
JC69 model distance
0.00
0.05
0.10
0.15
psaC Saturation (1st Pos)
Uncorrected genetic distance
JC69 model distance
0.00
0.02
0.04
0.06
psaC Saturation (2nd Pos)
Uncorrected genetic distance
JC69 model distance
0.00
0.01
0.02
0.03
psaC Saturation (3rd Pos)
Uncorrected genetic distance
JC69 model distance
0.0
0.1
0.2
0.3
0.4
0.5
0.6

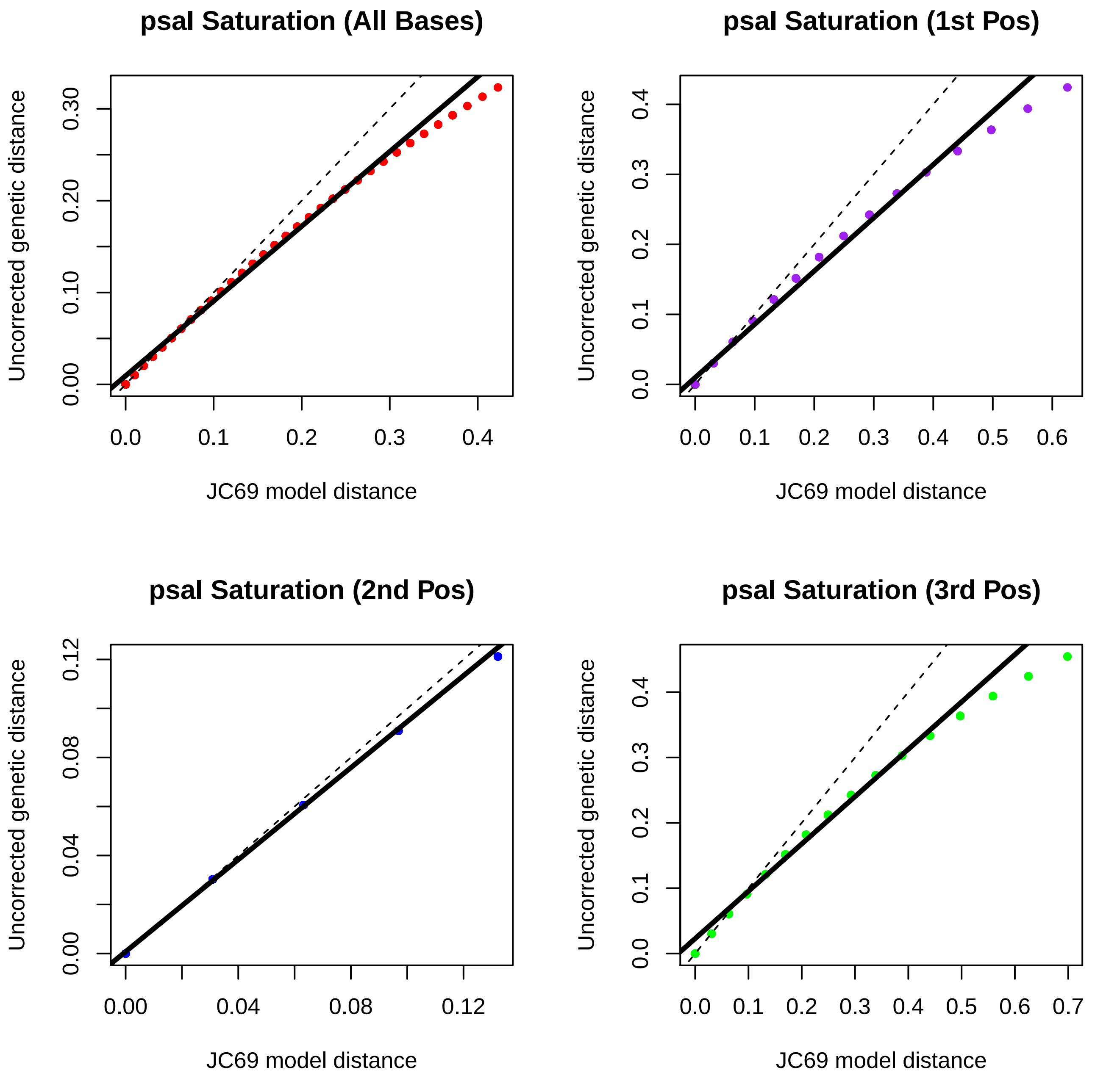
psal Saturation (All Bases)
psal Saturation (1st Pos)
psal Saturation (2nd Pos)
psal Saturation (3rd Pos)
Uncorrected genetic distance
JC69 model distance
0.00
0.10
0.20
0.30
0.0
0.1
0.2
0.3
0.4
0.5
0.6
0.7
0.04
0.08
0.12

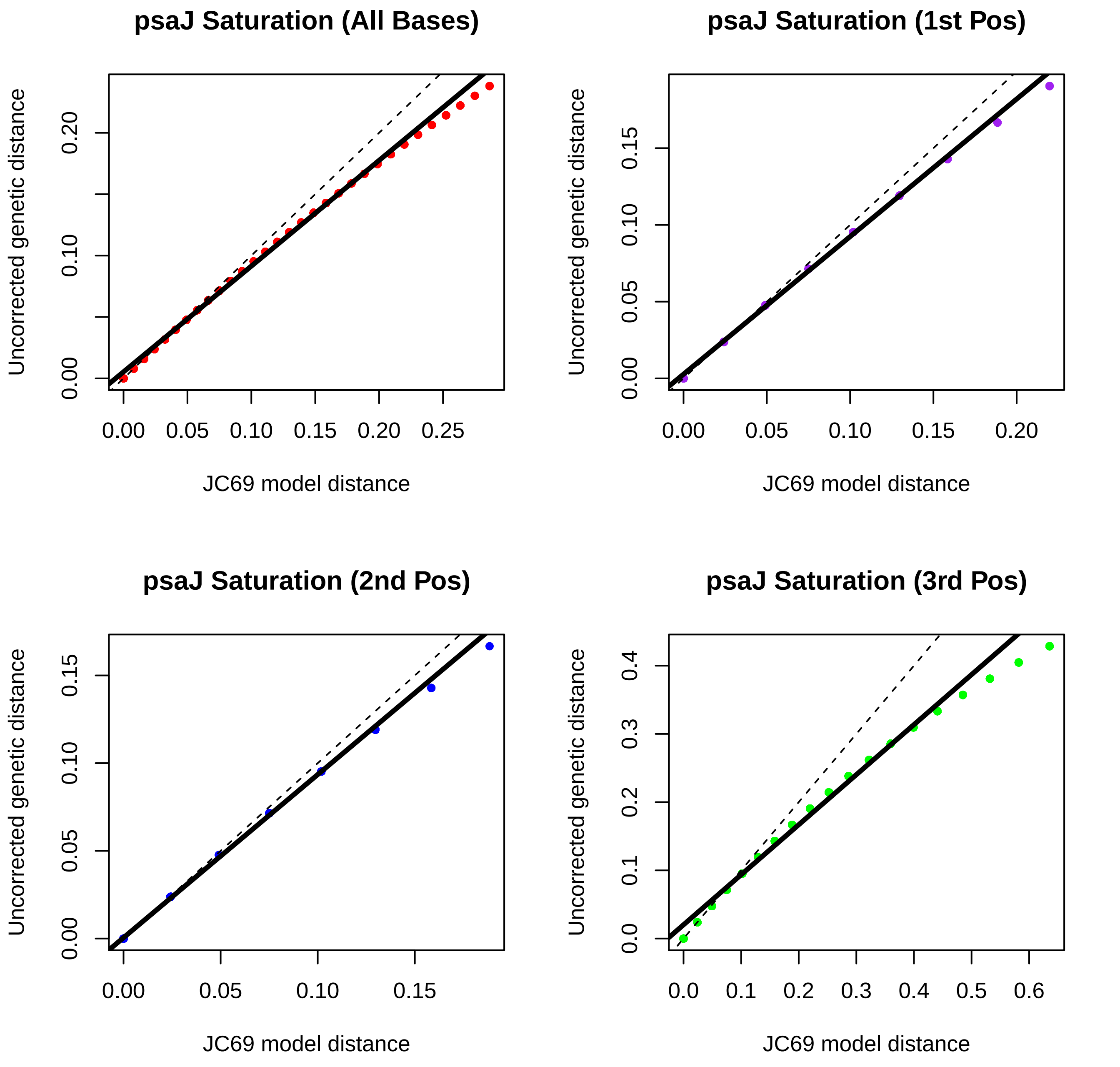
psaJ Saturation (All Bases)
Uncorrected genetic distance
0.00
0.10
0.20
0.00 0.05 0.10 0.15 0.20 0.25
JC69 model distance
psaJ Saturation (1st Pos)
Uncorrected genetic distance
0.00
0.05
0.10
0.15
0.00 0.05 0.10 0.15 0.20
JC69 model distance
psaJ Saturation (2nd Pos)
Uncorrected genetic distance
0.00
0.05
0.10
0.15
0.00 0.05 0.10 0.15
JC69 model distance
psaJ Saturation (3rd Pos)
Uncorrected genetic distance
0.0
0.1
0.2
0.3
0.4
0.0 0.1 0.2 0.3 0.4 0.5 0.6
JC69 model distance

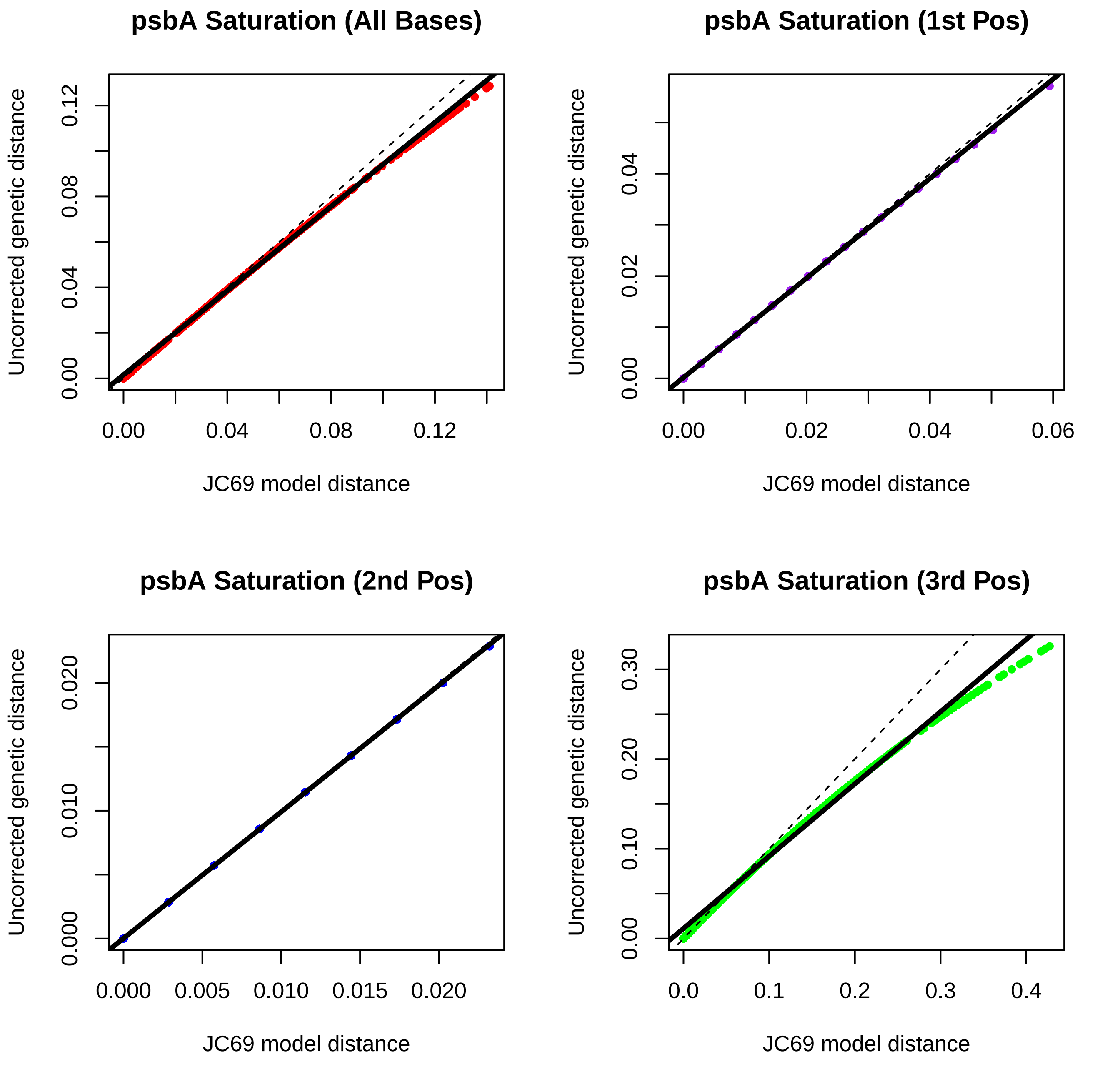
psbA Saturation (All Bases)
psbA Saturation (1st Pos)
psbA Saturation (2nd Pos)
psbA Saturation (3rd Pos)
Uncorrected genetic distance
JC69 model distance
0.00
0.04
0.08
0.12
0.02
0.06
0.000
0.005
0.010
0.015
0.020
0.0
0.1
0.2
0.3
0.4
0.10
0.20
0.30

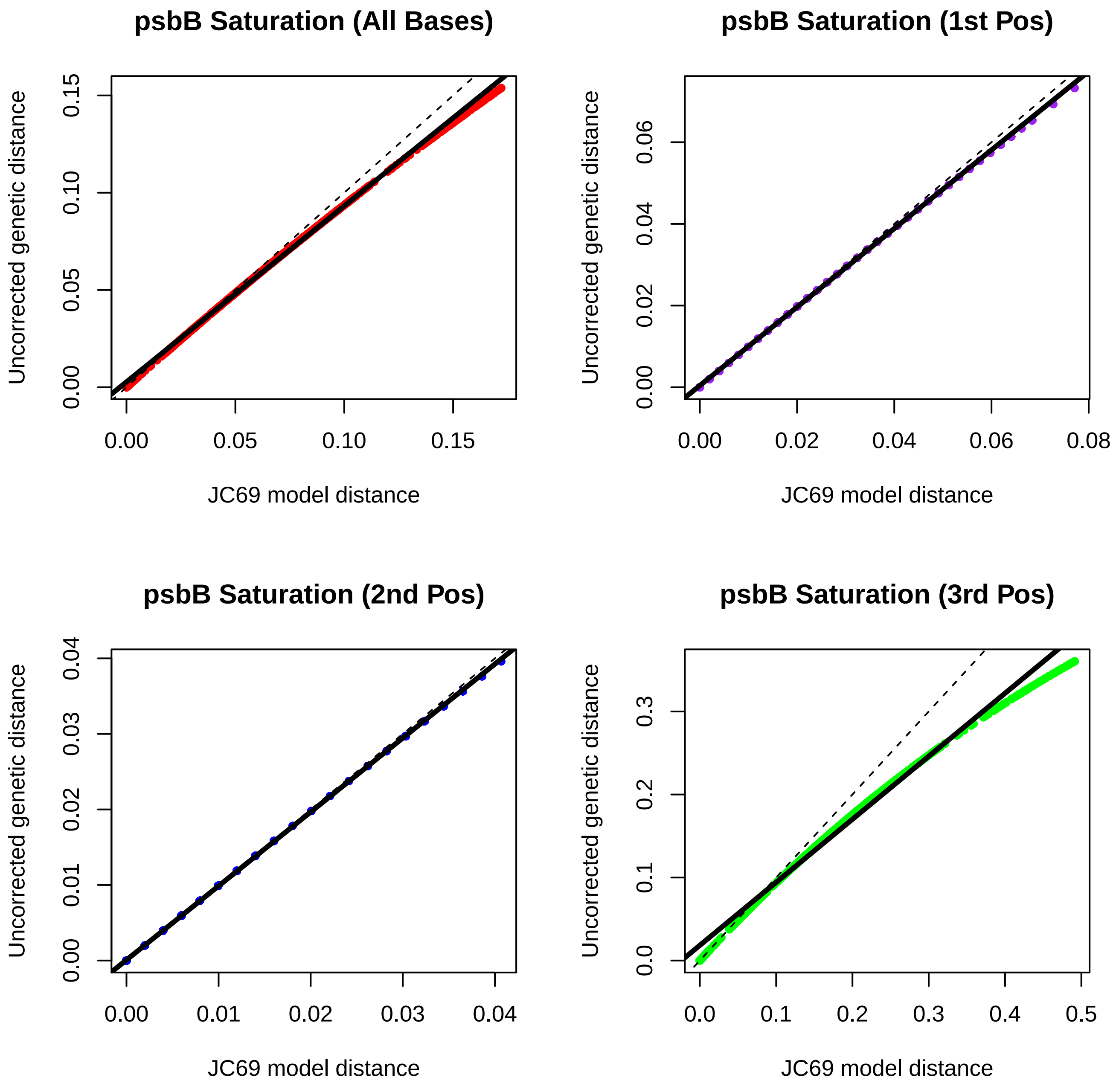

psbB Saturation (All Bases)
psbB Saturation (1st Pos)
psbB Saturation (2nd Pos)
psbB Saturation (3rd Pos)
Uncorrected genetic distance
JC69 model distance
0.00
0.05
0.10
0.15
0.02
0.04
0.06
0.08
0.01
0.03
0.0
0.1
0.2
0.3
0.4
0.5

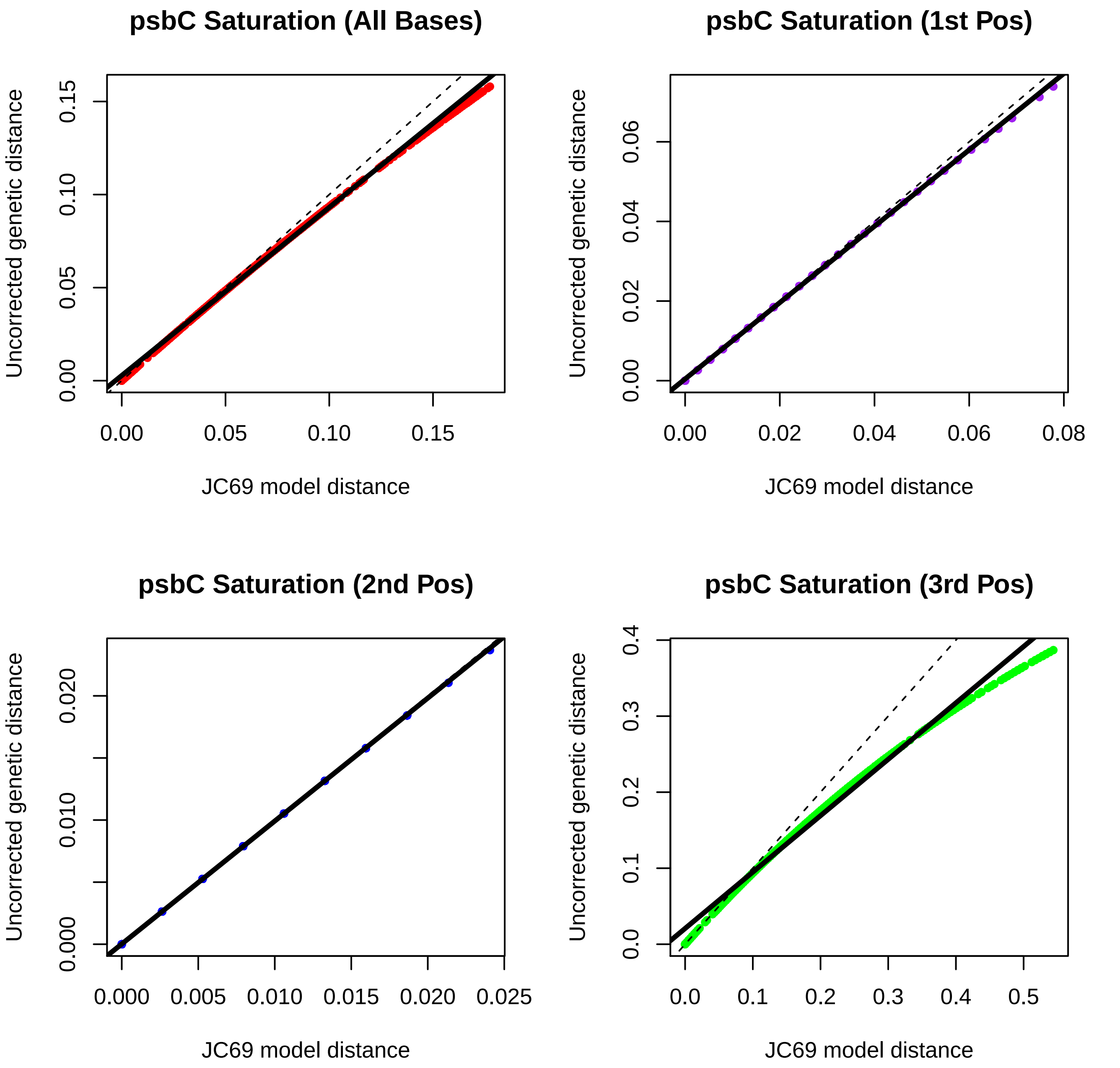
psbC Saturation (All Bases)
Uncorrected genetic distance
JC69 model distance
0.00
0.05
0.10
0.15
psbC Saturation (1st Pos)
Uncorrected genetic distance
JC69 model distance
0.00
0.02
0.04
0.06
0.08
psbC Saturation (2nd Pos)
Uncorrected genetic distance
JC69 model distance
0.000
0.005
0.010
0.015
0.020
0.025
psbC Saturation (3rd Pos)
Uncorrected genetic distance
JC69 model distance
0.0
0.1
0.2
0.3
0.4
0.5

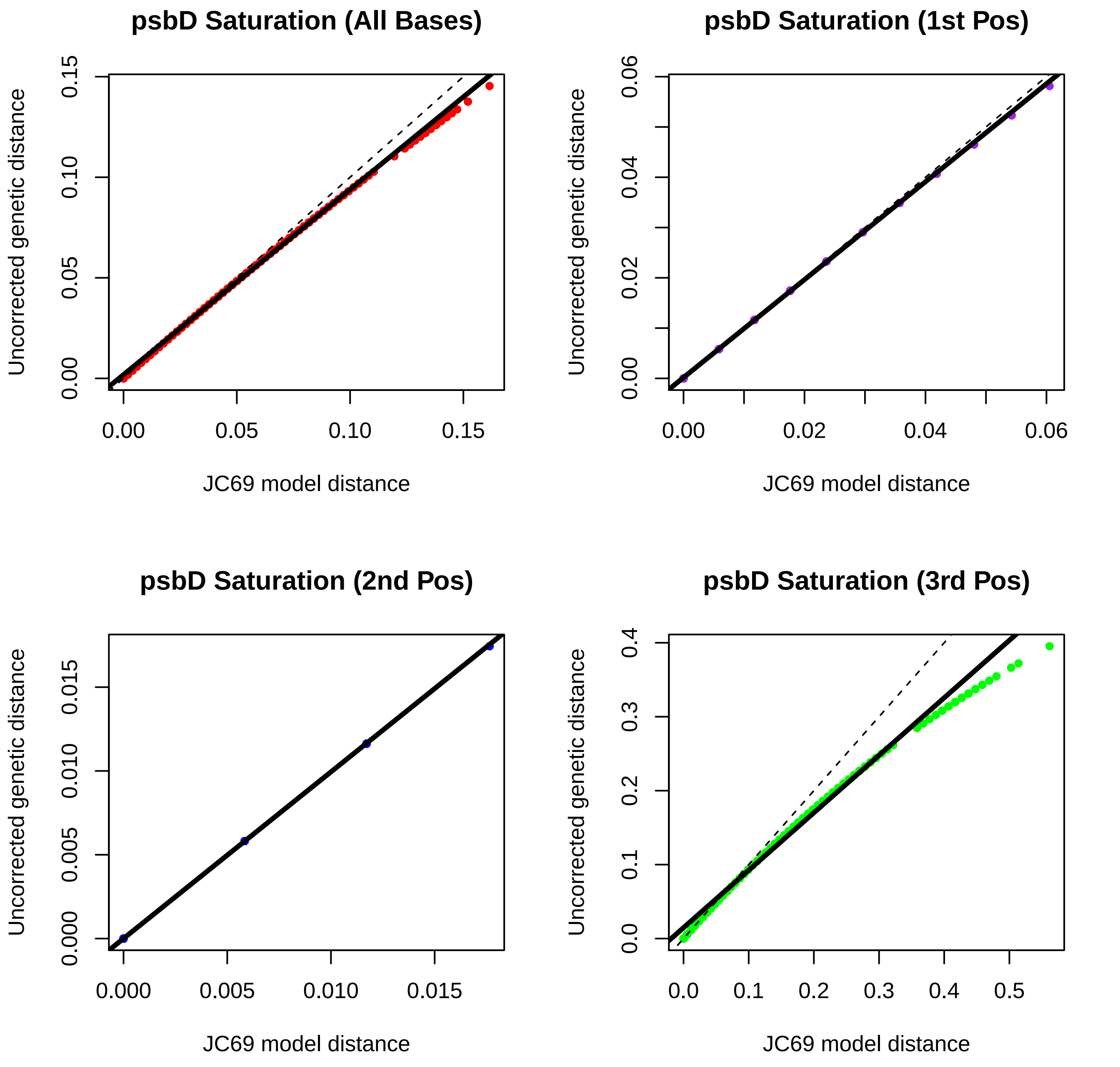
psbD Saturation (All Bases)
psbD Saturation (1st Pos)
psbD Saturation (2nd Pos)
psbD Saturation (3rd Pos)
Uncorrected genetic distance
JC69 model distance
0.00
0.05
0.10
0.15
0.02
0.04
0.06
0.000
0.005
0.010
0.015
0.0
0.1
0.2
0.3
0.4
0.5

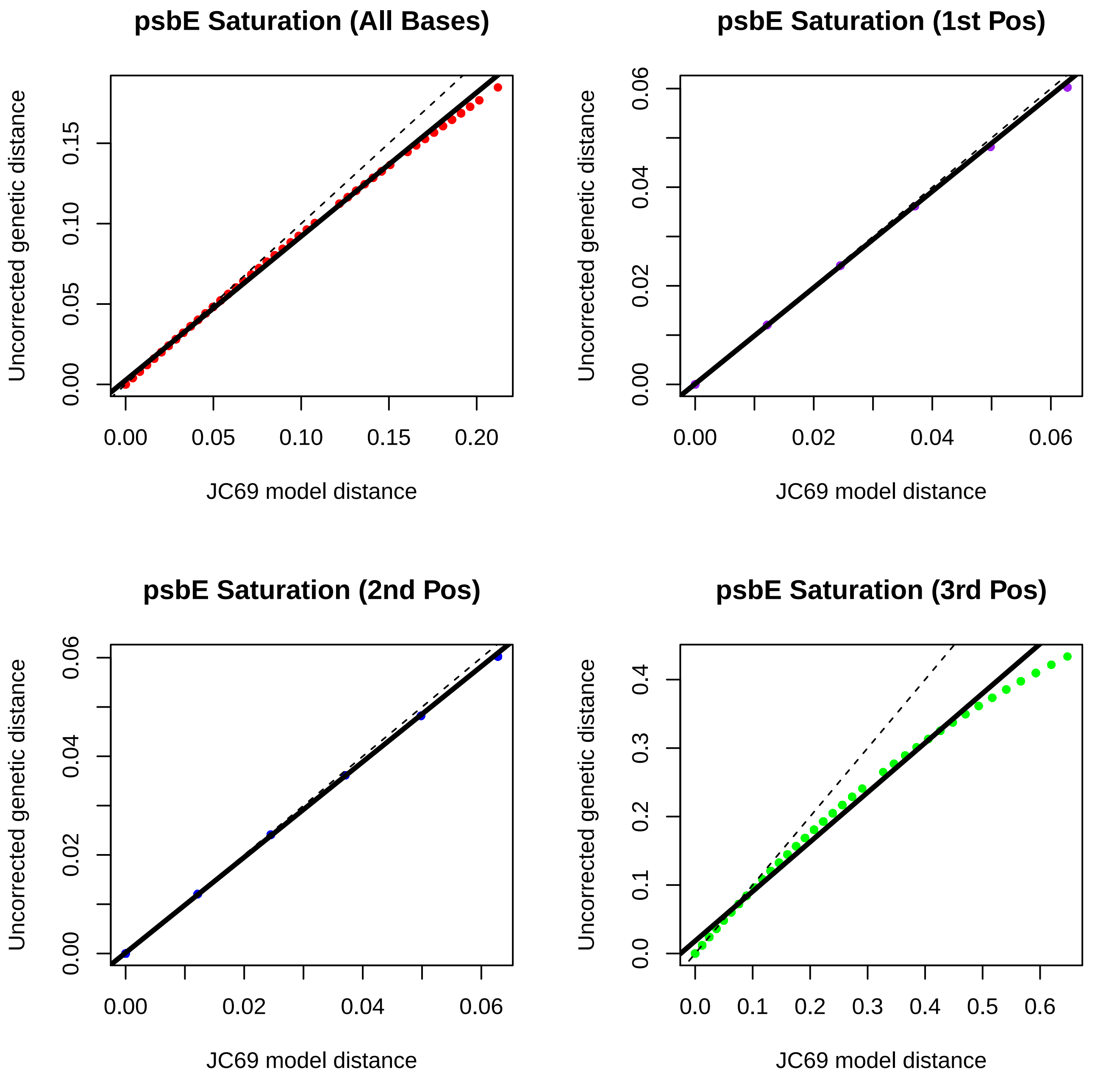

psbE Saturation (All Bases)
psbE Saturation (1st Pos)
psbE Saturation (2nd Pos)
psbE Saturation (3rd Pos)
Uncorrected genetic distance
JC69 model distance
0.00
0.05
0.10
0.15
0.20
0.02
0.04
0.06
0.0
0.1
0.2
0.3
0.4
0.5
0.6

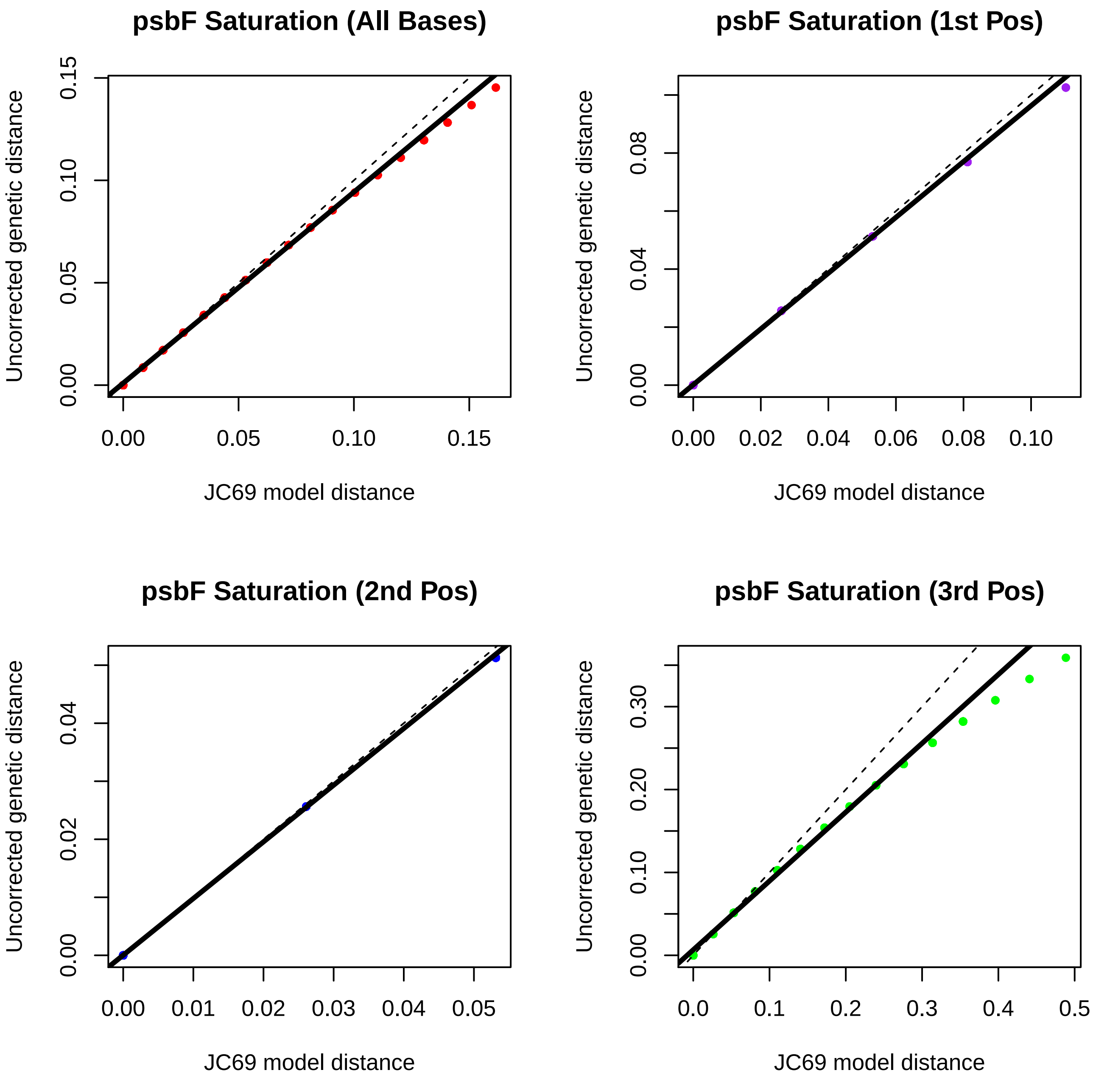
psbF Saturation (All Bases)
psbF Saturation (1st Pos)
psbF Saturation (2nd Pos)
psbF Saturation (3rd Pos)
Uncorrected genetic distance
JC69 model distance
0.00
0.05
0.10
0.15
0.02
0.04
0.06
0.08
0.01
0.03
0.0
0.1
0.2
0.3
0.4
0.5
0.20
0.30

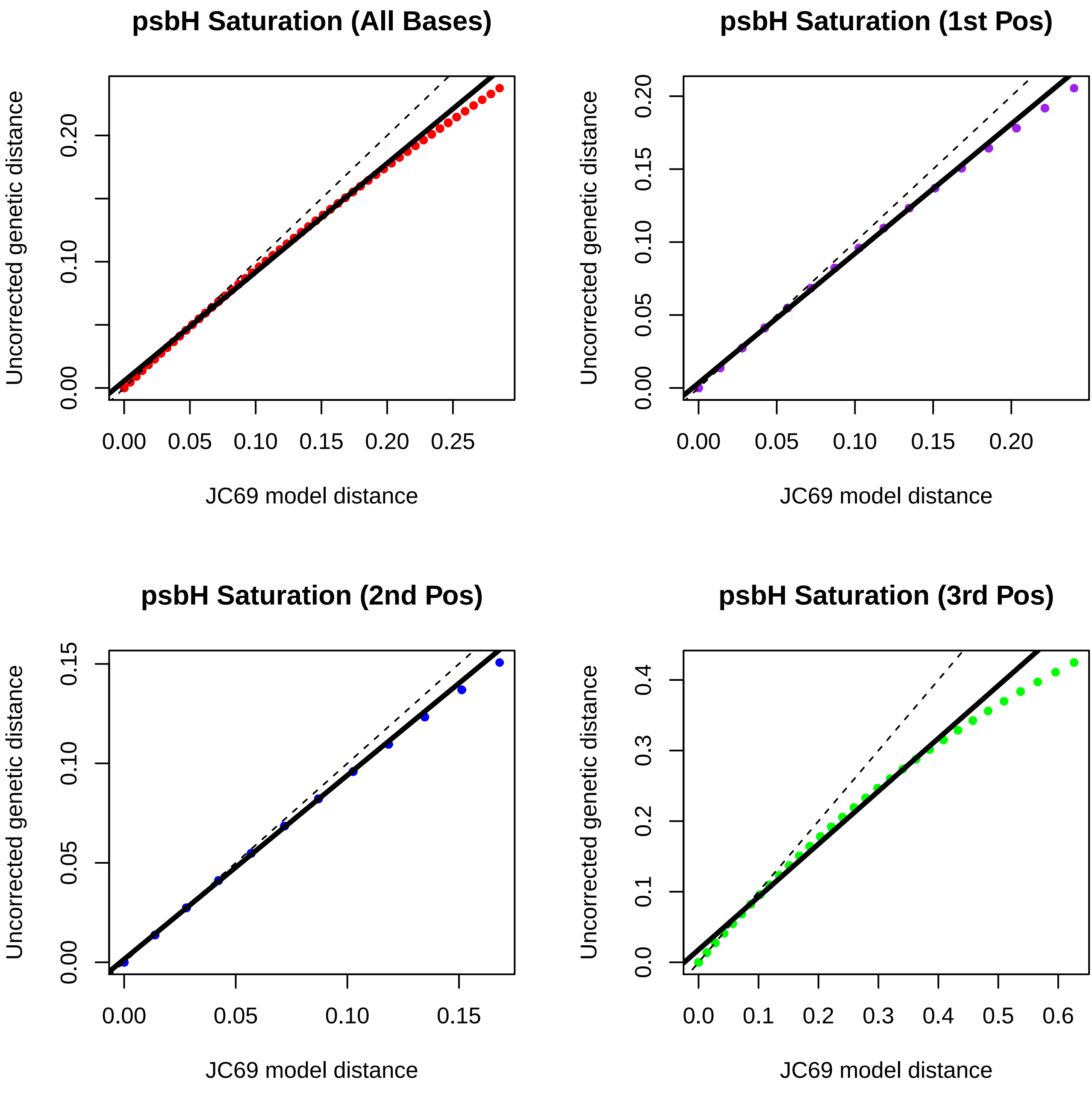

psbH Saturation (All Bases)
Uncorrected genetic distance
0.00
0.10
0.20
0.00
0.05
0.10
0.15
0.20
0.25
JC69 model distance
psbH Saturation (1st Pos)
Uncorrected genetic distance
0.00
0.05
0.10
0.15
0.20
0.00
0.05
0.10
0.15
0.20
JC69 model distance
psbH Saturation (2nd Pos)
Uncorrected genetic distance
0.00
0.05
0.10
0.15
0.00
0.05
0.10
0.15
JC69 model distance
psbH Saturation (3rd Pos)
Uncorrected genetic distance
0.0
0.1
0.2
0.3
0.4
0.0
0.1
0.2
0.3
0.4
0.5
0.6
JC69 model distance

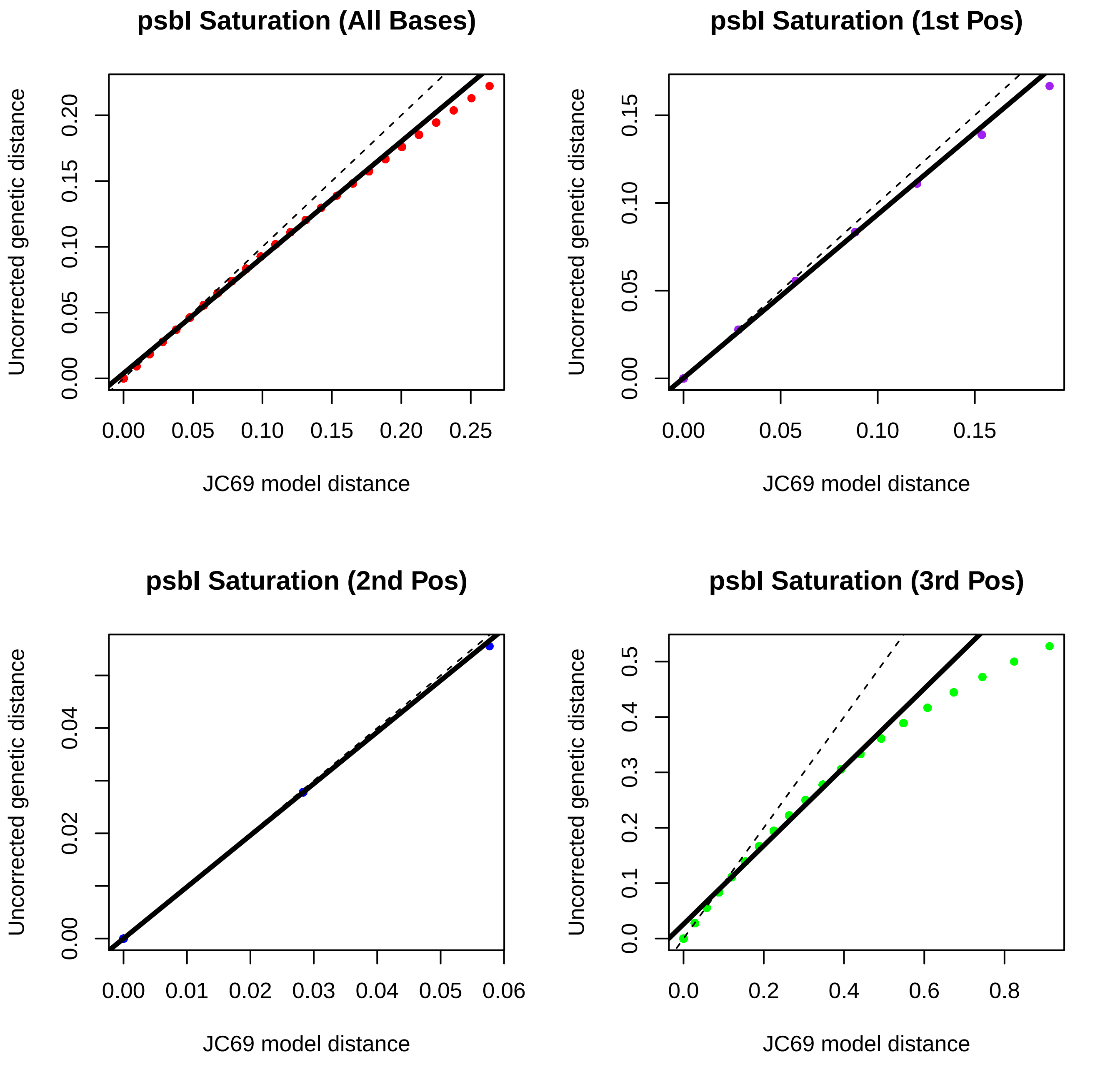

psbI Saturation (All Bases)
psbI Saturation (1st Pos)
psbI Saturation (2nd Pos)
psbI Saturation (3rd Pos)
Uncorrected genetic distance
JC69 model distance
0.00
0.05
0.10
0.15
0.20
0.25
0.01
0.02
0.03
0.04
0.06
0.0
0.1
0.2
0.3
0.4
0.5
0.6
0.8

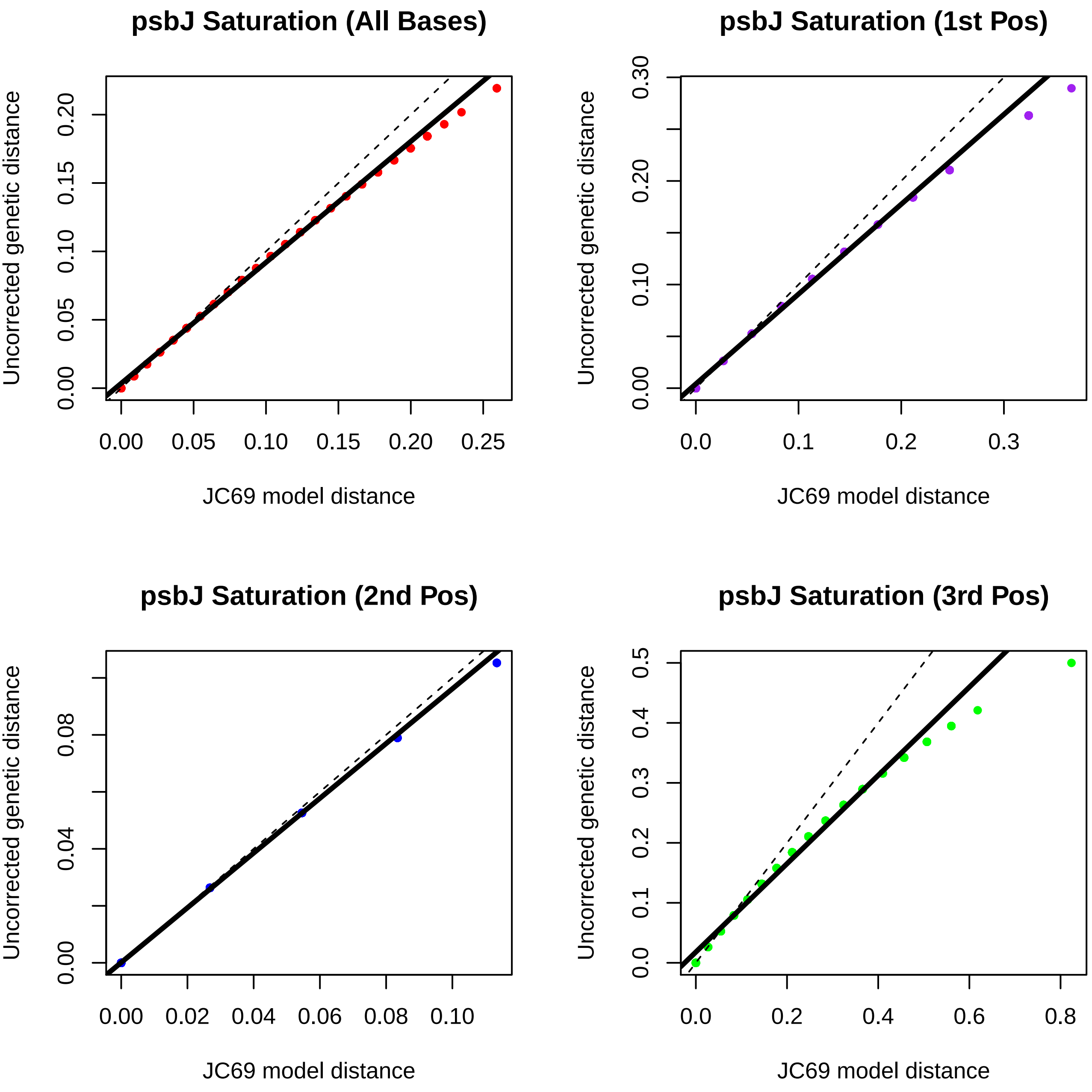
psbJ Saturation (All Bases)
psbJ Saturation (1st Pos)
psbJ Saturation (2nd Pos)
psbJ Saturation (3rd Pos)
Uncorrected genetic distance
JC69 model distance
0.00
0.05
0.10
0.15
0.20
0.25
0.0
0.1
0.2
0.3
0.30
0.02
0.04
0.06
0.08
0.4
0.5
0.6
0.8

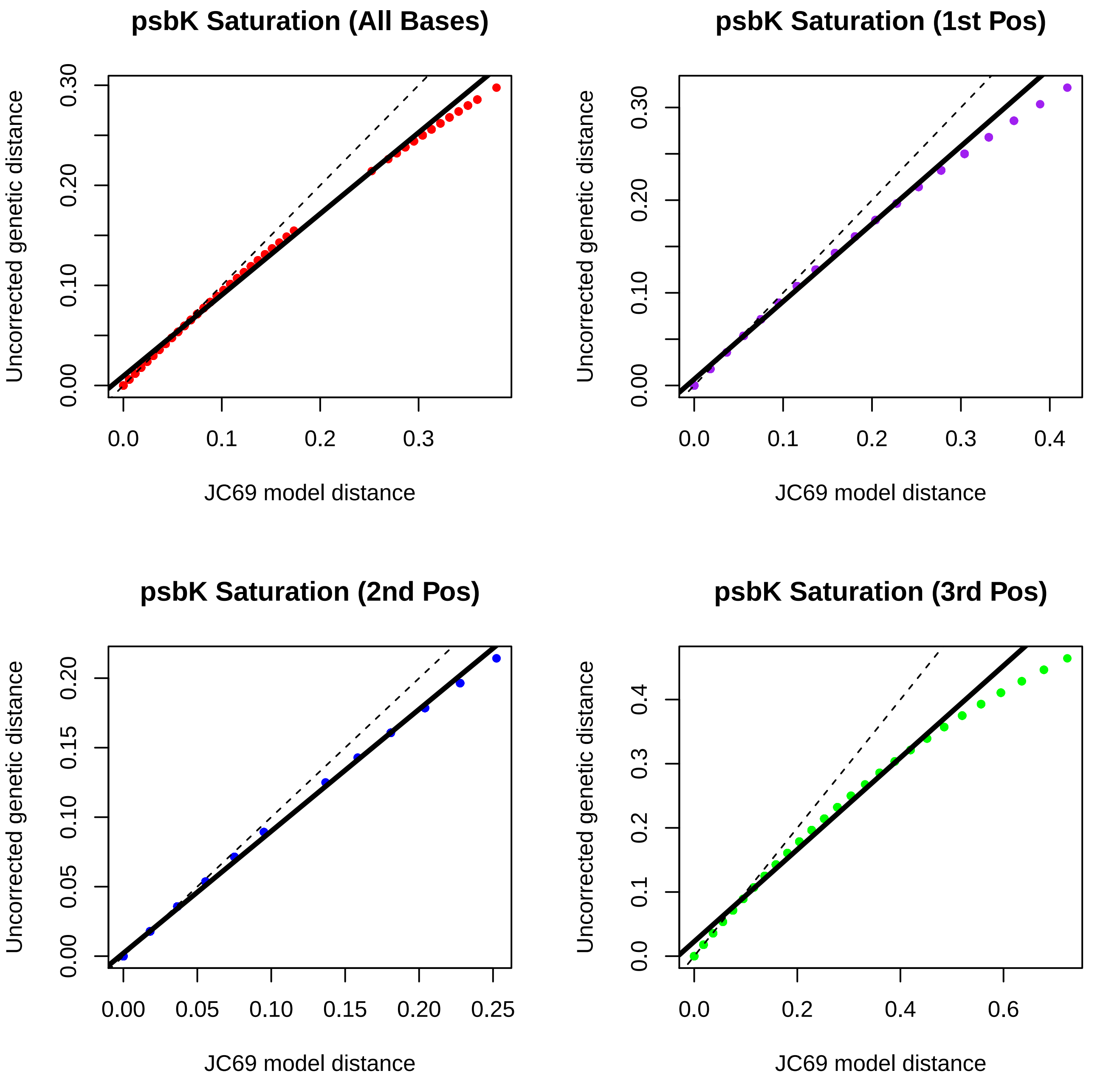
psbK Saturation (All Bases)
psbK Saturation (1st Pos)
psbK Saturation (2nd Pos)
psbK Saturation (3rd Pos)
Uncorrected genetic distance
JC69 model distance
0.00
0.10
0.20
0.30
0.0
0.1
0.2
0.3
0.4
0.05
0.15
0.25
0.6

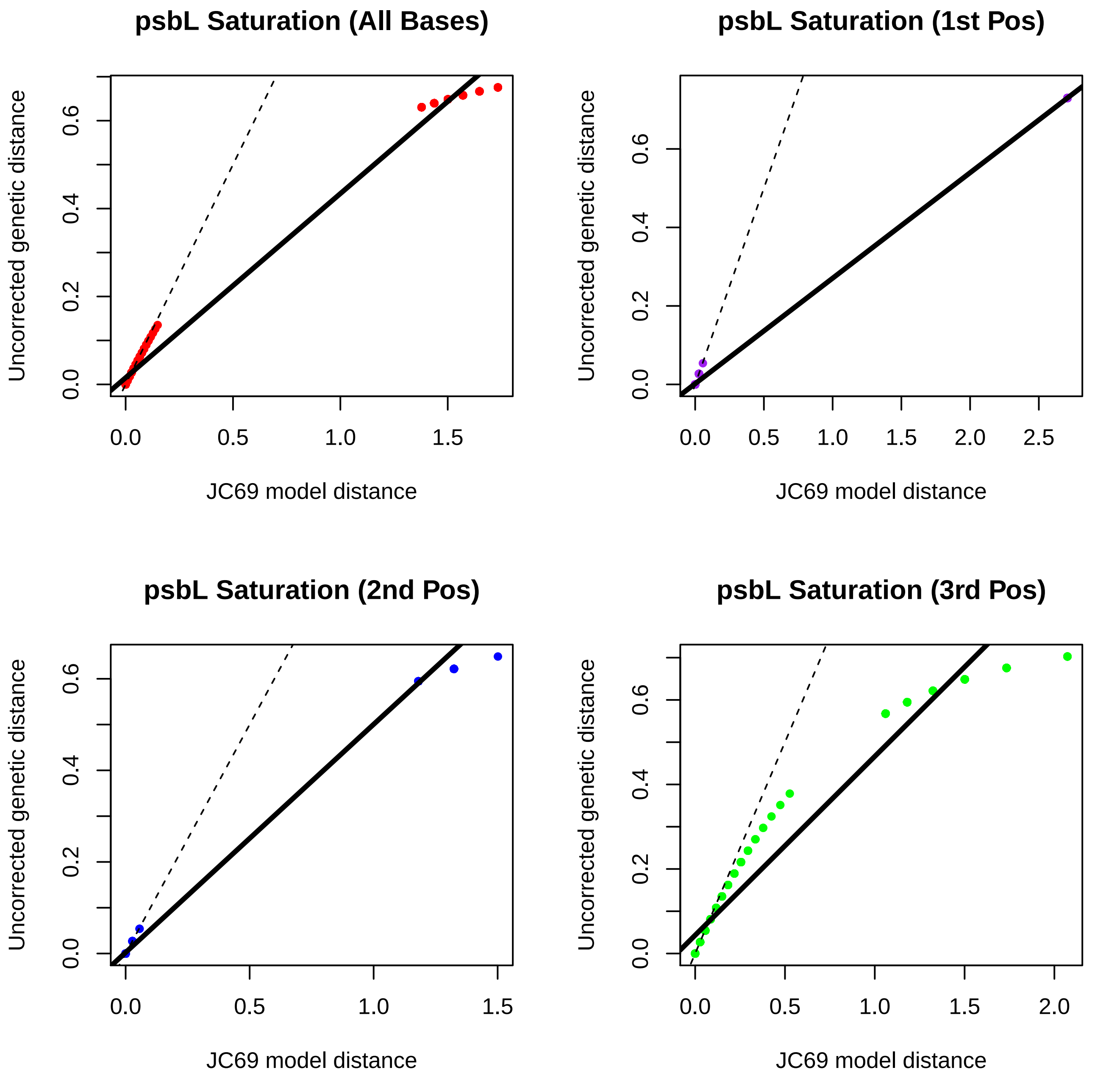
psbL Saturation (All Bases)
psbL Saturation (1st Pos)
psbL Saturation (2nd Pos)
psbL Saturation (3rd Pos)
Uncorrected genetic distance
JC69 model distance
0.0
0.2
0.4
0.6
0.5
1.0
1.5
2.0
2.5

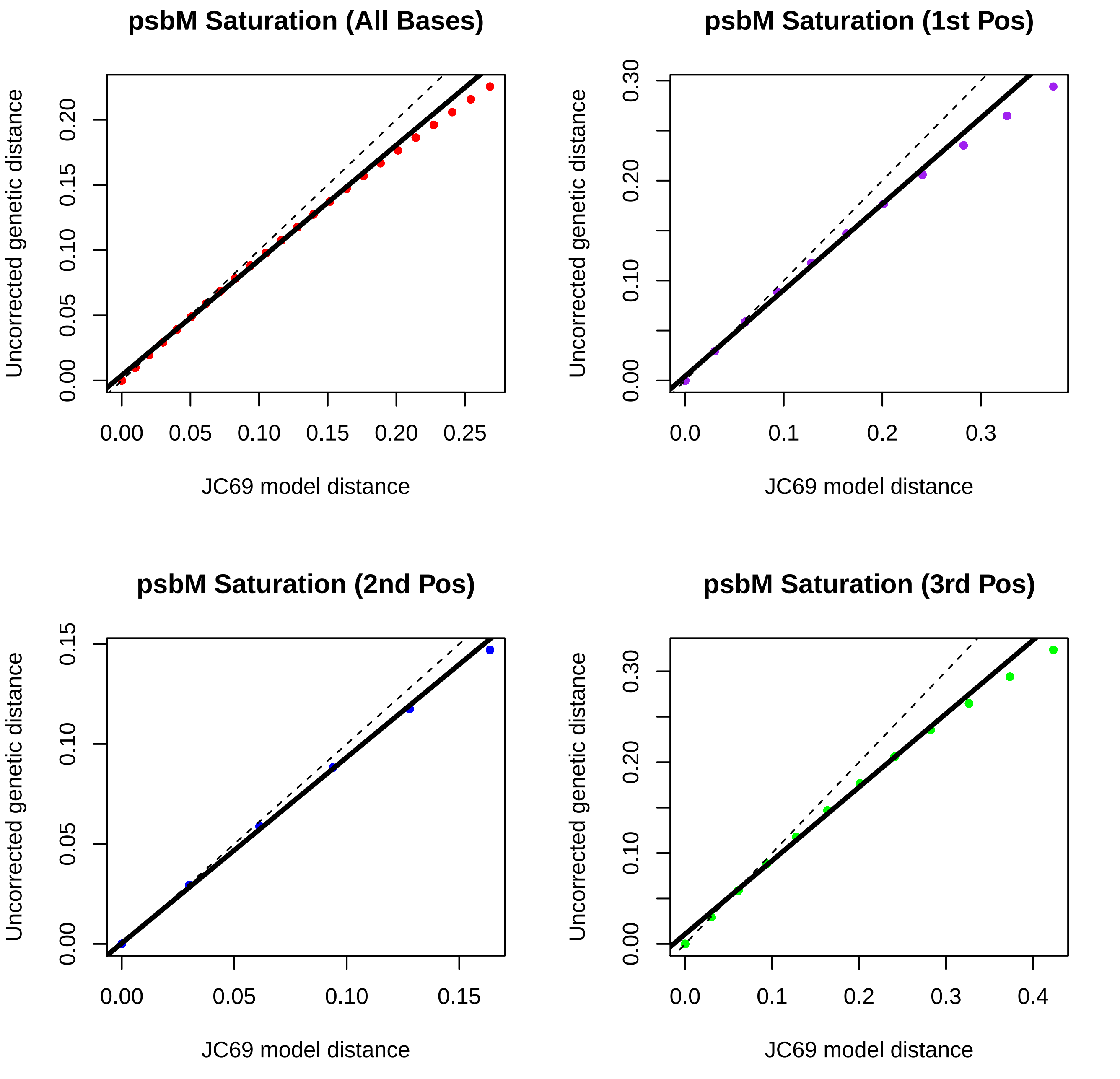
psbM Saturation (All Bases)
psbM Saturation (1st Pos)
psbM Saturation (2nd Pos)
psbM Saturation (3rd Pos)
Uncorrected genetic distance
JC69 model distance
0.00
0.05
0.10
0.15
0.20
0.25
0.30
0.0
0.1
0.2
0.3
0.4

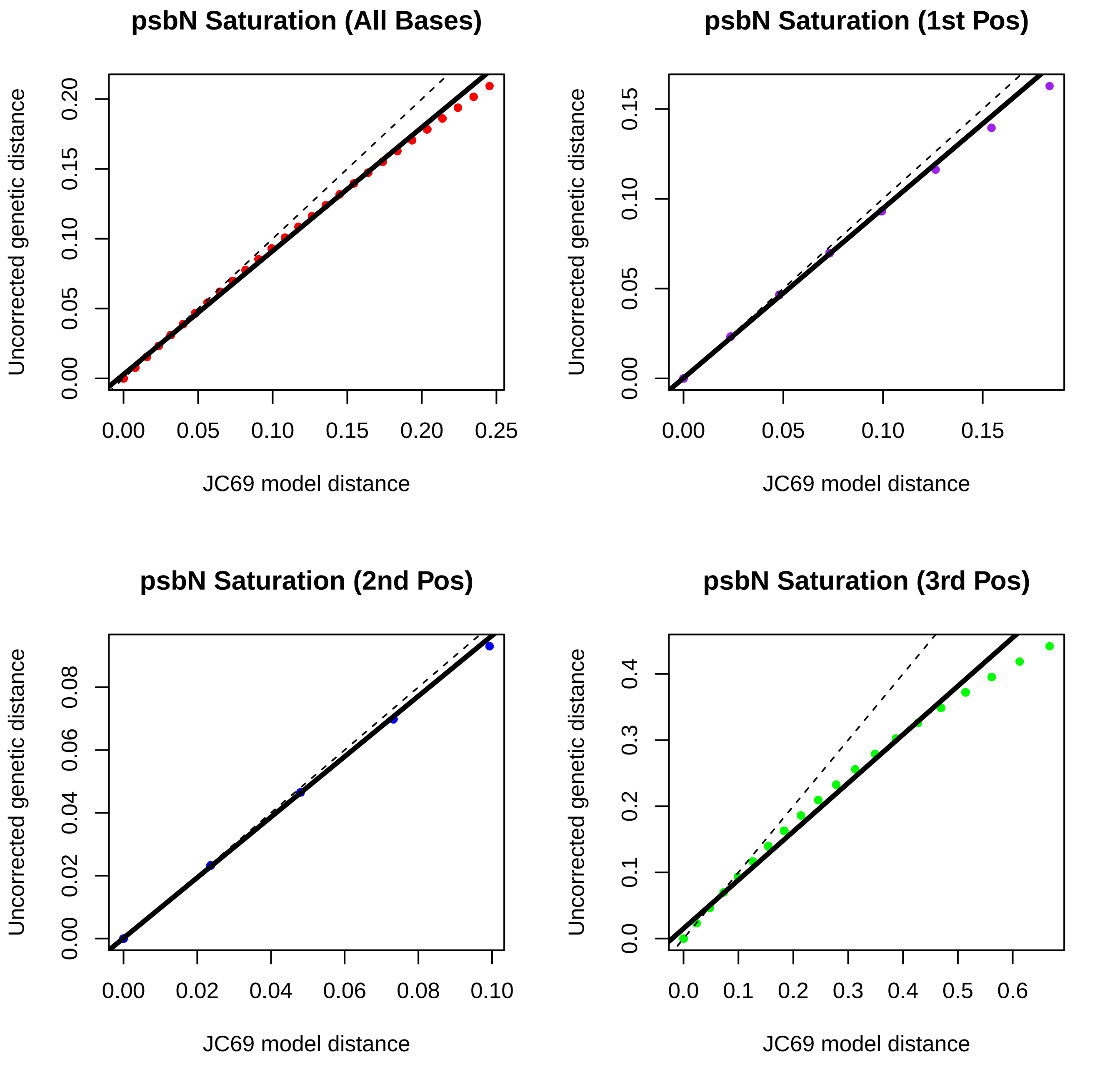
psbN Saturation (All Bases)
psbN Saturation (1st Pos)
psbN Saturation (2nd Pos)
psbN Saturation (3rd Pos)
Uncorrected genetic distance
JC69 model distance
0.00
0.05
0.10
0.15
0.20
0.25
0.02
0.04
0.06
0.08
0.0
0.1
0.2
0.3
0.4
0.5
0.6

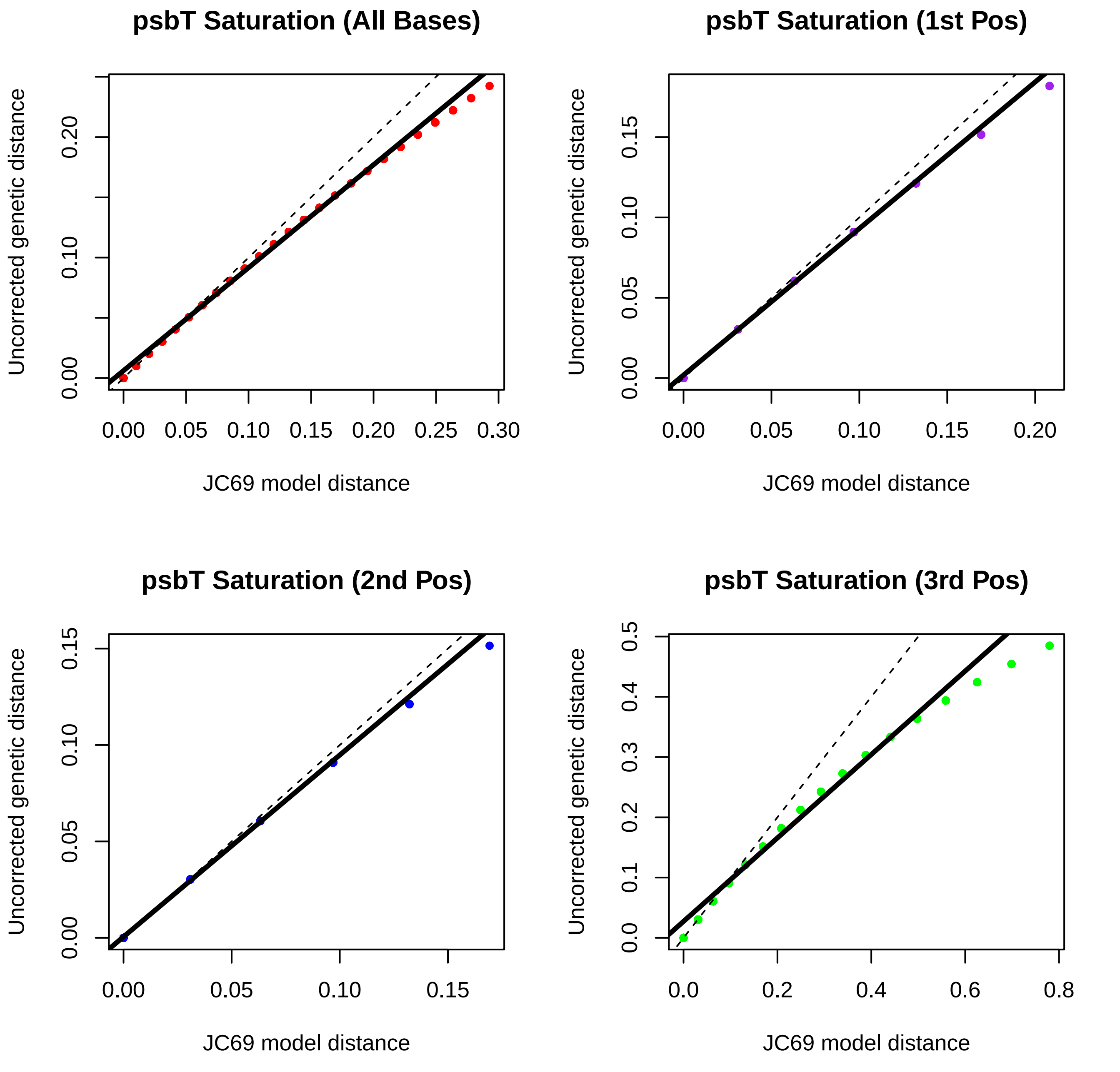
psbT Saturation (All Bases)
psbT Saturation (1st Pos)
psbT Saturation (2nd Pos)
psbT Saturation (3rd Pos)
Uncorrected genetic distance
JC69 model distance
0.00
0.05
0.10
0.15
0.20
0.25
0.30
0.0
0.1
0.2
0.3
0.4
0.5
0.6
0.8

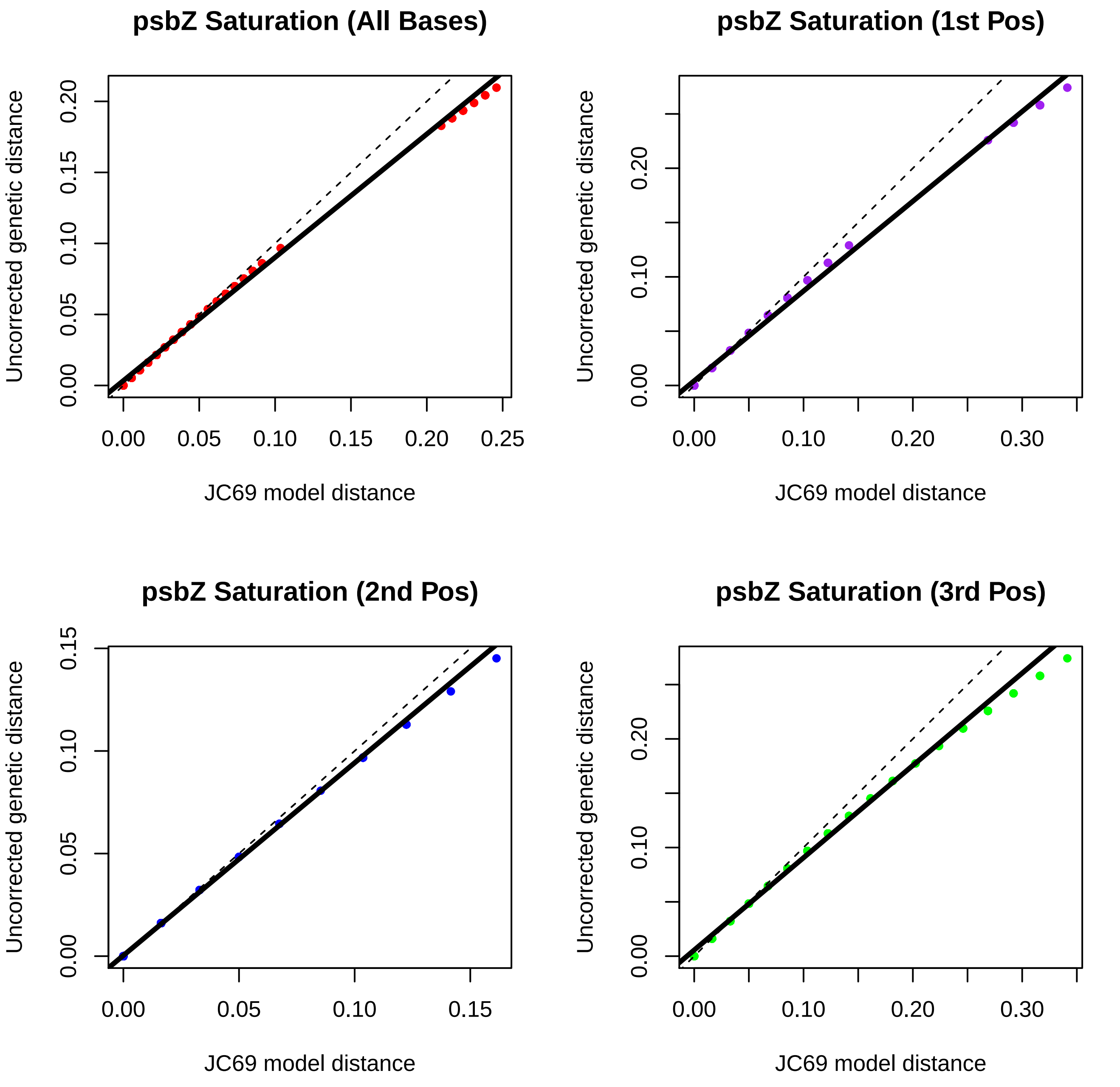
psbZ Saturation (All Bases)
psbZ Saturation (1st Pos)
psbZ Saturation (2nd Pos)
psbZ Saturation (3rd Pos)
Uncorrected genetic distance
JC69 model distance
0.00
0.05
0.10
0.15
0.20
0.25
0.30

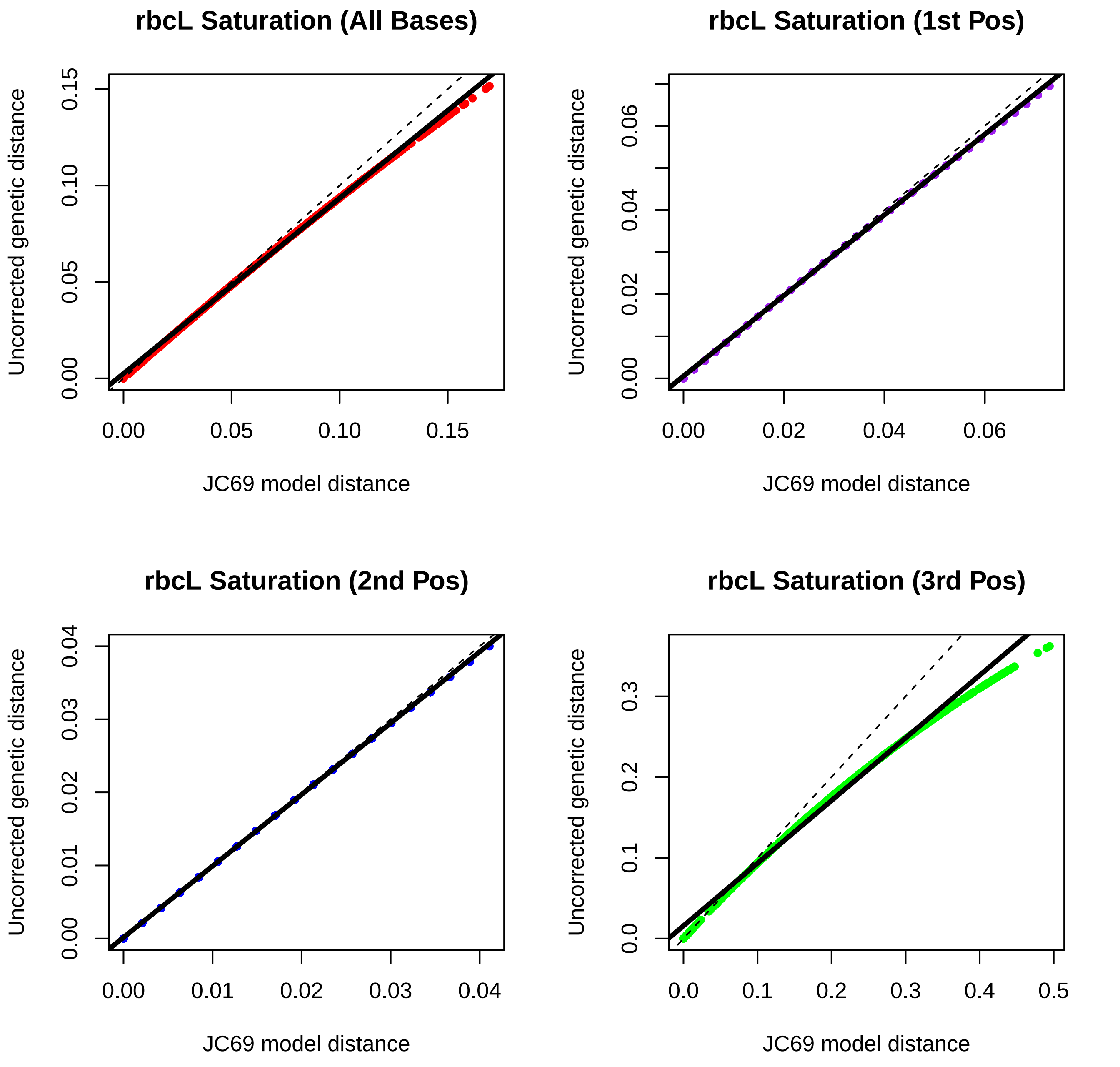
rbcL Saturation (All Bases)
Uncorrected genetic distance
0.00
0.05
0.10
0.15
JC69 model distance
rbcL Saturation (1st Pos)
0.00
0.02
0.04
0.06
rbcL Saturation (2nd Pos)
0.00
0.01
0.02
0.03
0.04
rbcL Saturation (3rd Pos)
0.0
0.1
0.2
0.3
0.4
0.5

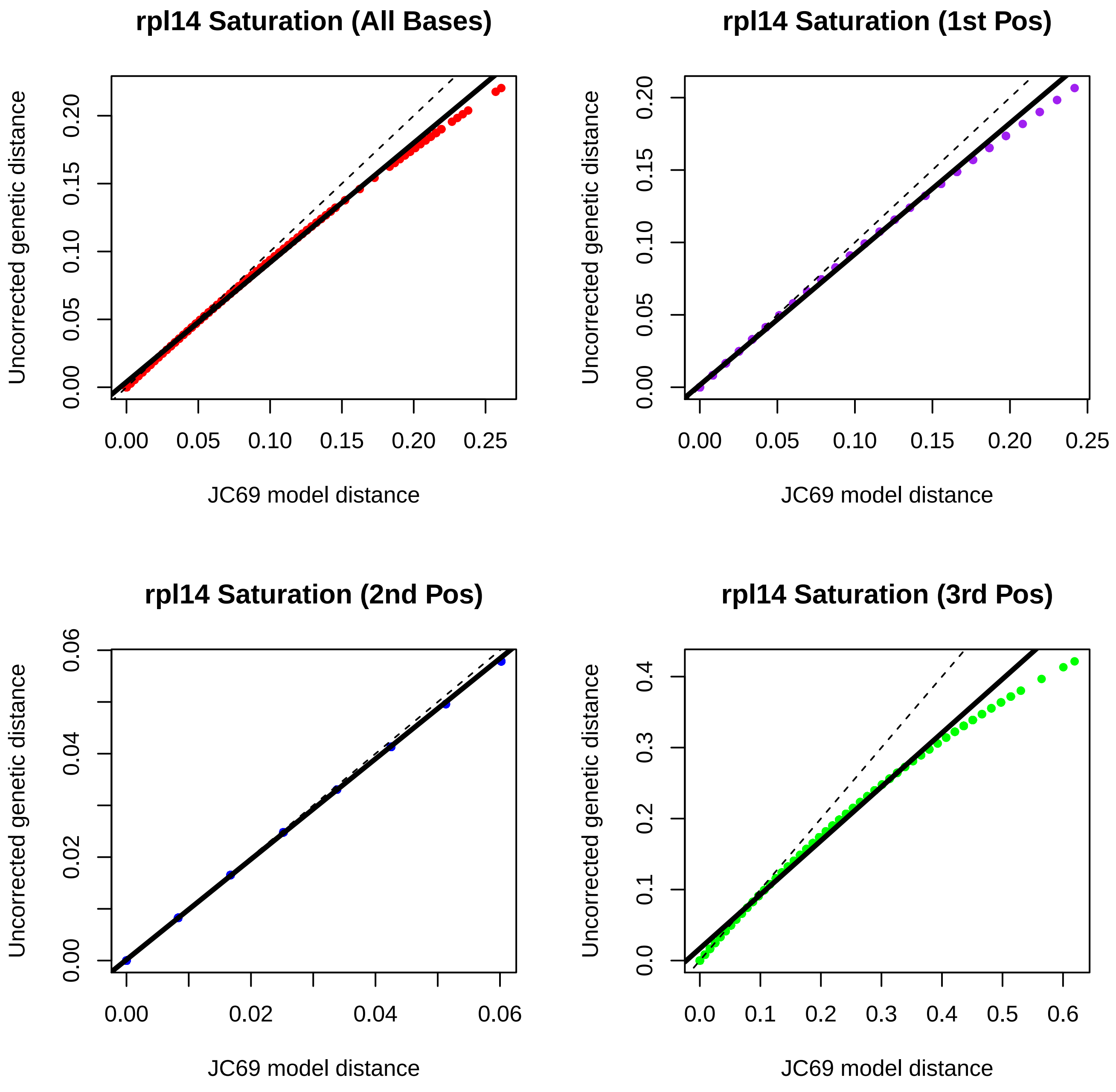

rpl14 Saturation (All Bases)
rpl14 Saturation (1st Pos)
rpl14 Saturation (2nd Pos)
rpl14 Saturation (3rd Pos)
Uncorrected genetic distance
JC69 model distance
0.00
0.05
0.10
0.15
0.20
0.25
0.02
0.04
0.06
0.0
0.1
0.2
0.3
0.4
0.5
0.6

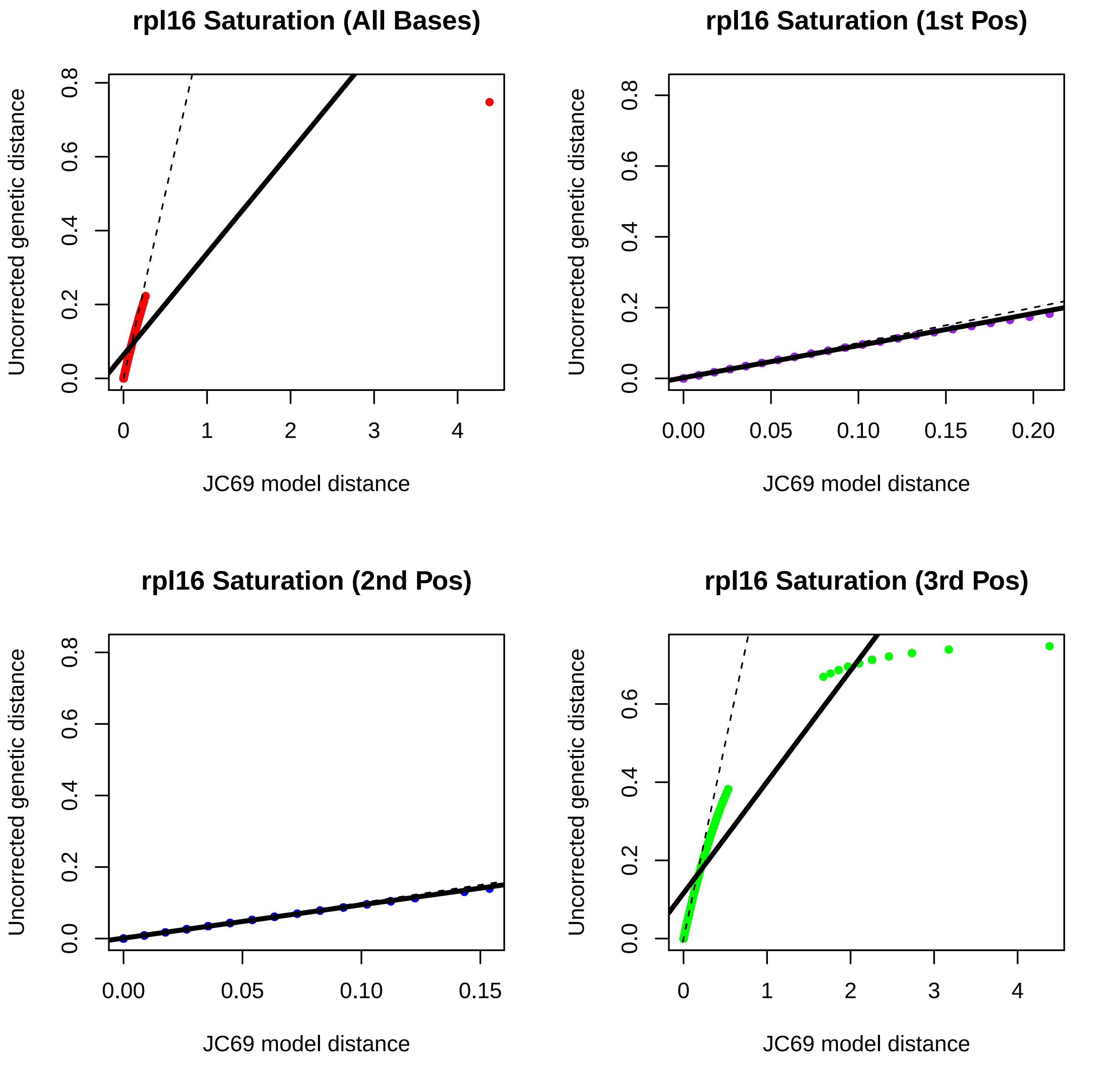
rpl16 Saturation (All Bases)
rpl16 Saturation (1st Pos)
rpl16 Saturation (2nd Pos)
rpl16 Saturation (3rd Pos)
Uncorrected genetic distance
JC69 model distance
0.0
0.2
0.4
0.6
0.8
0
1
2
3
4
0.00
0.05
0.10
0.15
0.20

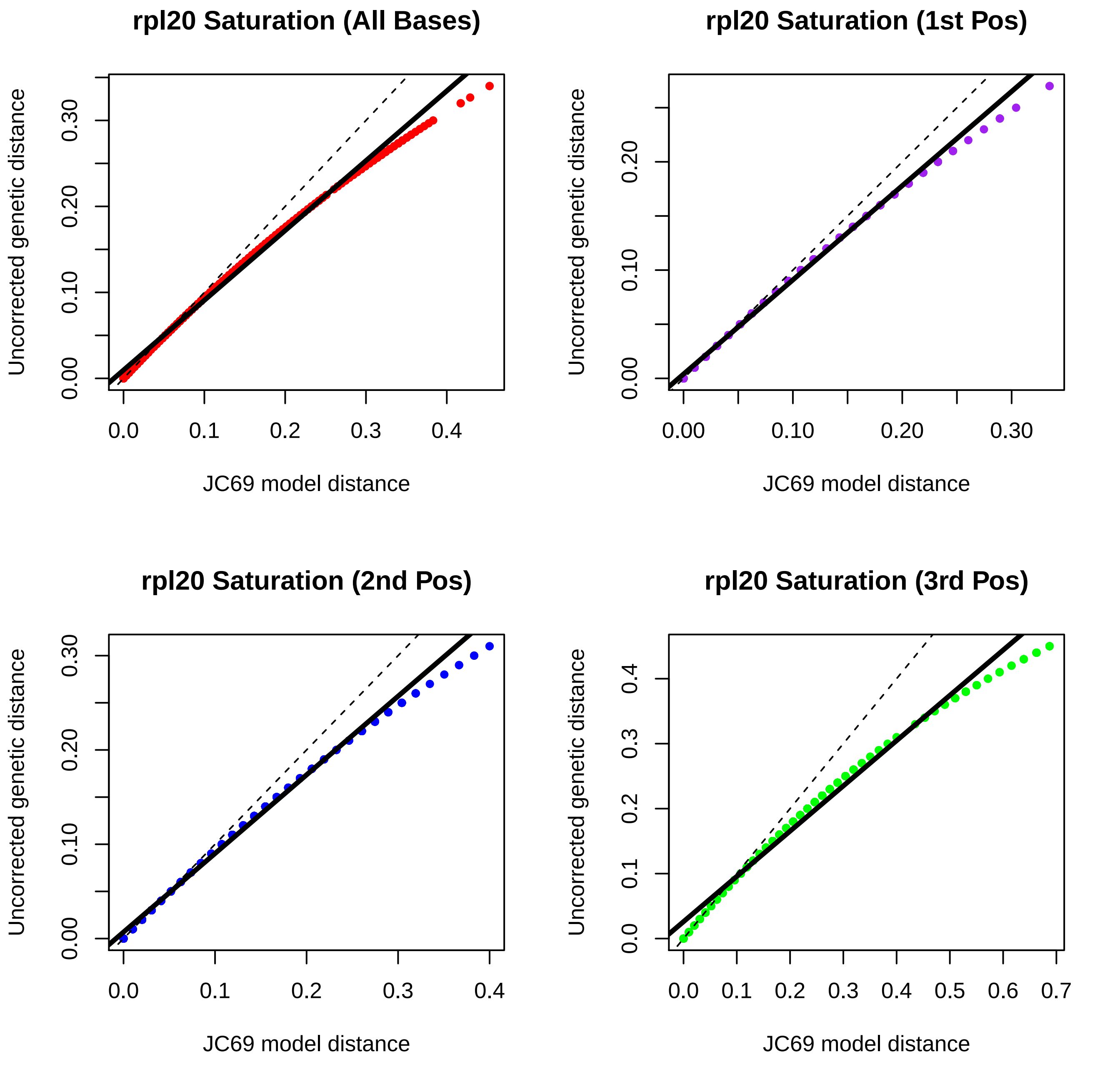

rpl20 Saturation (All Bases)
Uncorrected genetic distance
JC69 model distance
0.00
0.10
0.20
0.30
0.0
0.1
0.2
0.3
0.4
rpl20 Saturation (1st Pos)
Uncorrected genetic distance
JC69 model distance
0.00
0.10
0.20
0.00
0.10
0.20
0.30
rpl20 Saturation (2nd Pos)
Uncorrected genetic distance
JC69 model distance
0.00
0.10
0.20
0.30
0.0
0.1
0.2
0.3
0.4
rpl20 Saturation (3rd Pos)
Uncorrected genetic distance
JC69 model distance
0.0
0.1
0.2
0.3
0.4
0.0
0.1
0.2
0.3
0.4
0.5
0.6
0.7

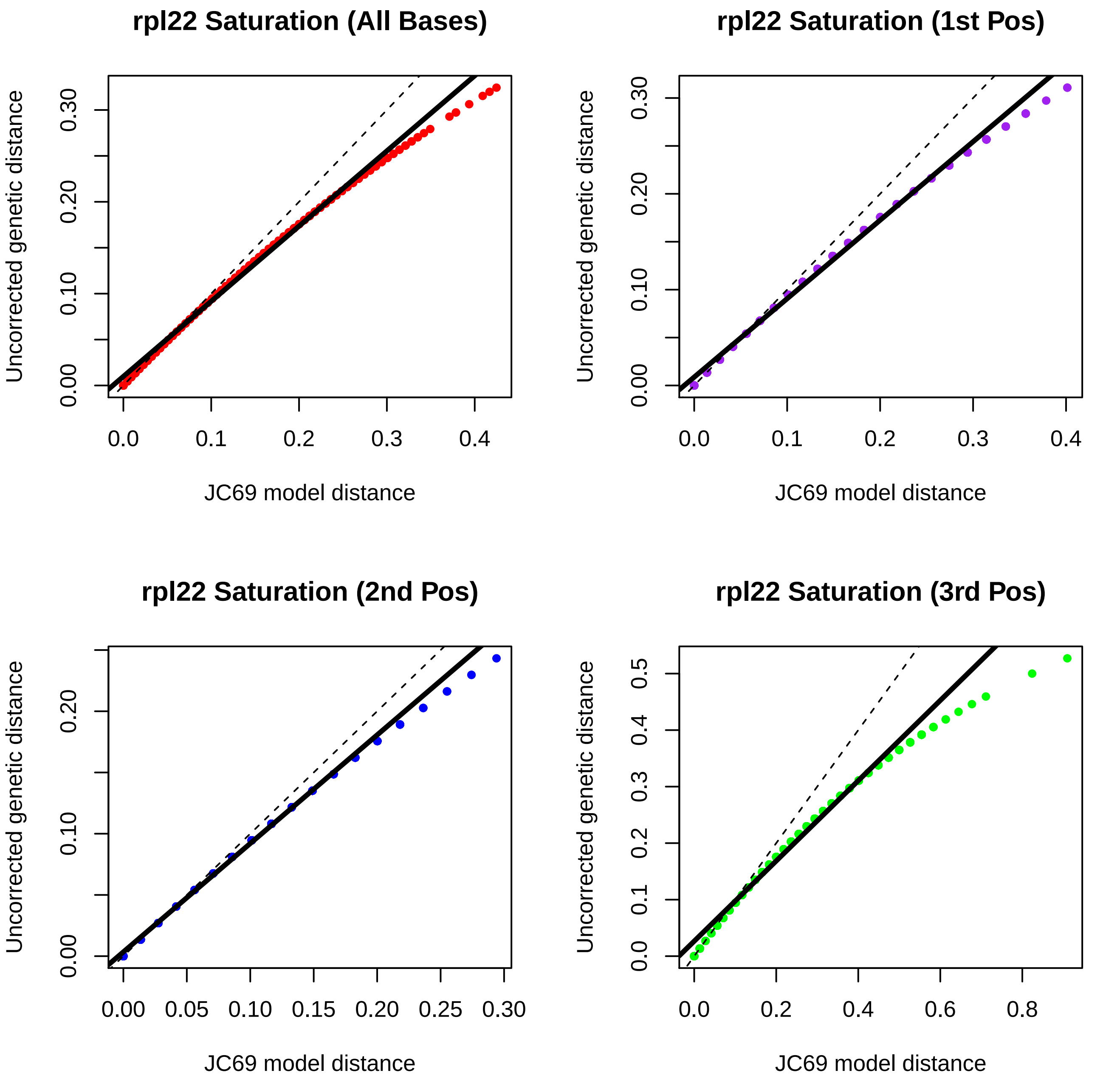
rpl22 Saturation (All Bases)
rpl22 Saturation (1st Pos)
rpl22 Saturation (2nd Pos)
rpl22 Saturation (3rd Pos)
Uncorrected genetic distance
JC69 model distance
0.00
0.10
0.20
0.30
0.0
0.1
0.2
0.3
0.4
0.05
0.15
0.25
0.5
0.6
0.8

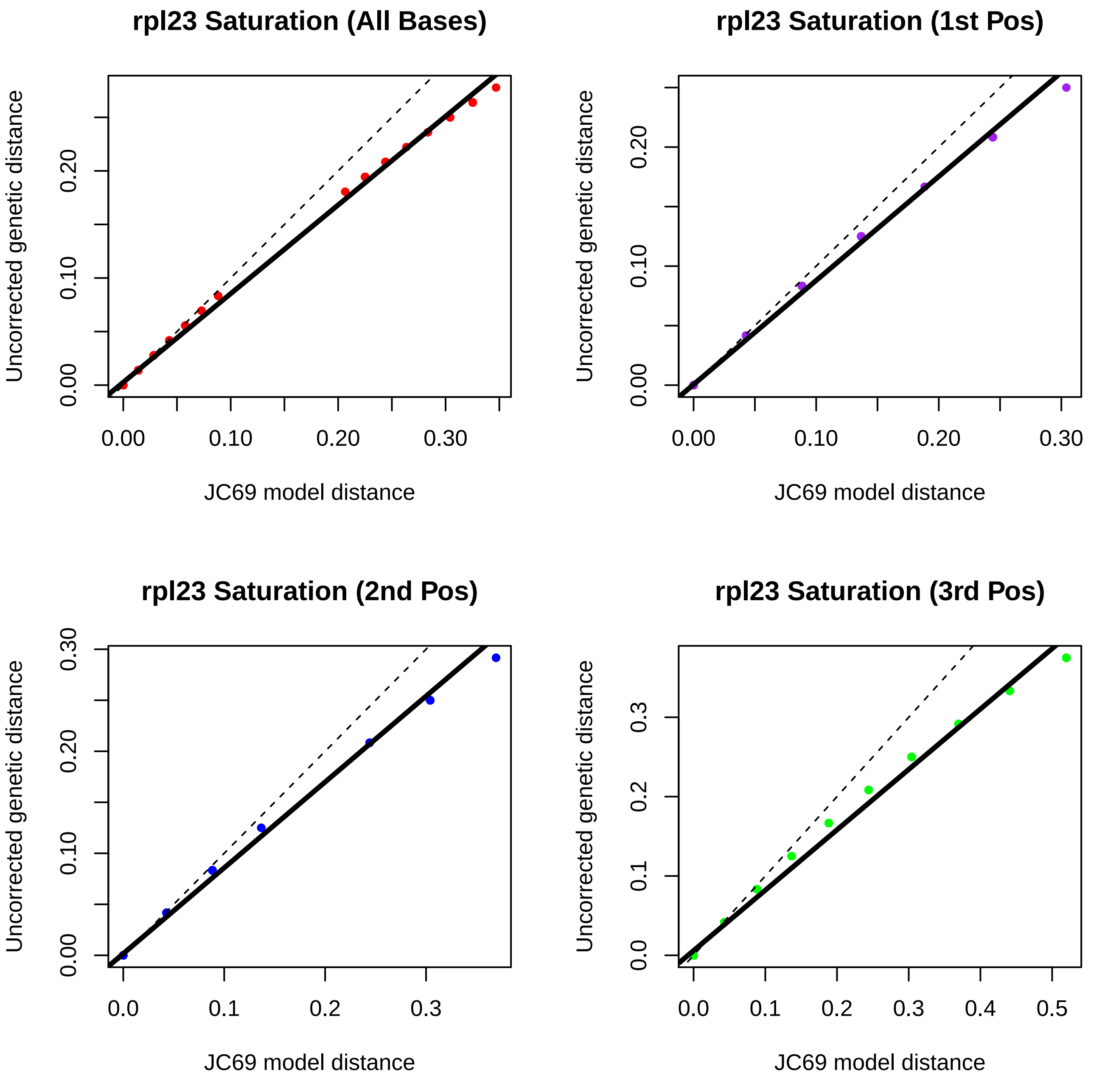
rpl23 Saturation (All Bases)
Uncorrected genetic distance
0.00
0.10
0.20
JC69 model distance
0.00
0.10
0.20
0.30
rpl23 Saturation (1st Pos)
Uncorrected genetic distance
0.00
0.10
0.20
JC69 model distance
0.00
0.10
0.20
0.30
rpl23 Saturation (2nd Pos)
Uncorrected genetic distance
0.00
0.10
0.20
0.30
JC69 model distance
0.0
0.1
0.2
0.3
rpl23 Saturation (3rd Pos)
Uncorrected genetic distance
0.0
0.1
0.2
0.3
JC69 model distance
0.0
0.1
0.2
0.3
0.4
0.5

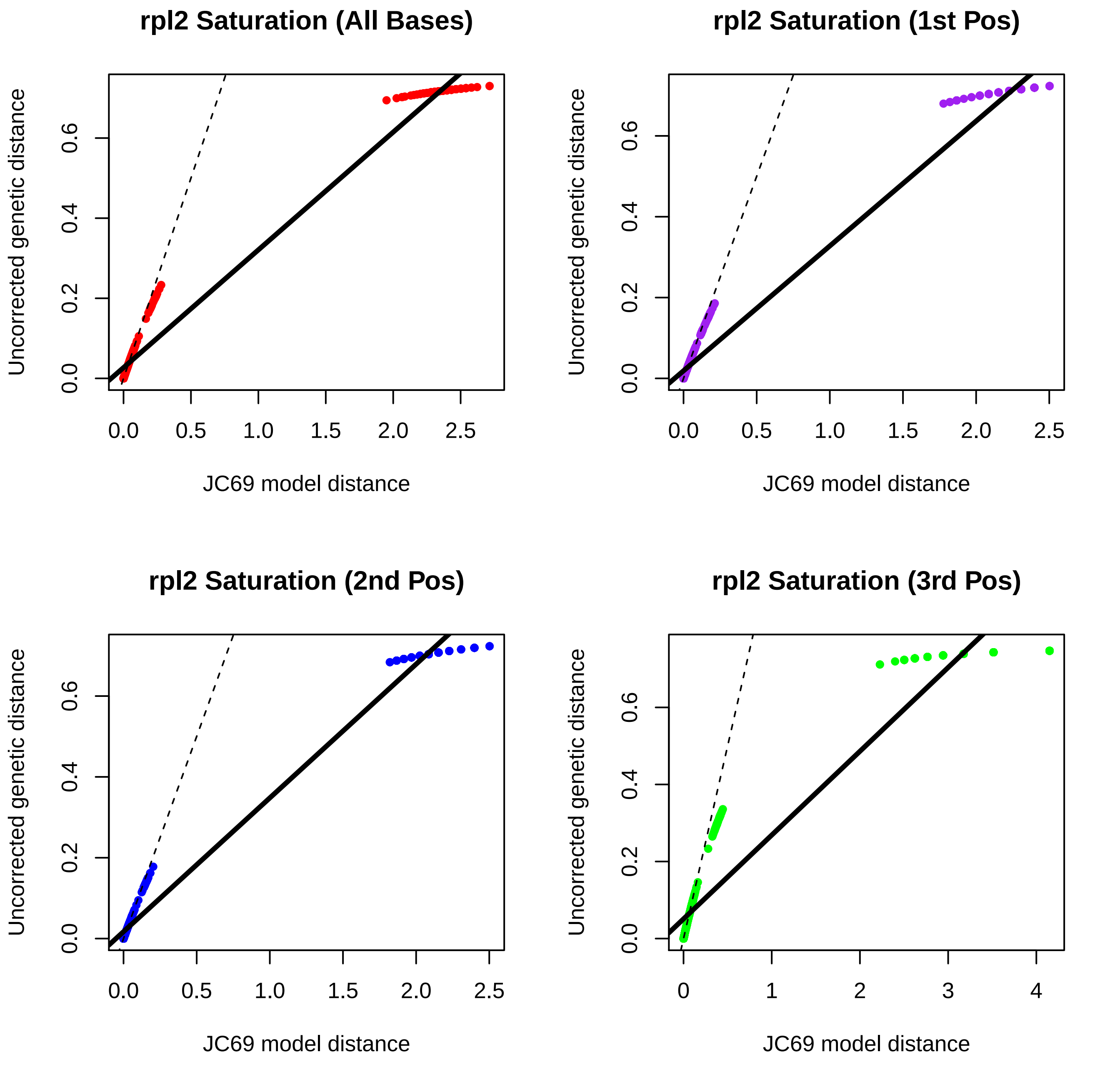
rpl2 Saturation (All Bases)
rpl2 Saturation (1st Pos)
rpl2 Saturation (2nd Pos)
rpl2 Saturation (3rd Pos)
Uncorrected genetic distance
JC69 model distance
0.0
0.2
0.4
0.6
0.5
1.0
1.5
2.0
2.5
0
1
2
3
4

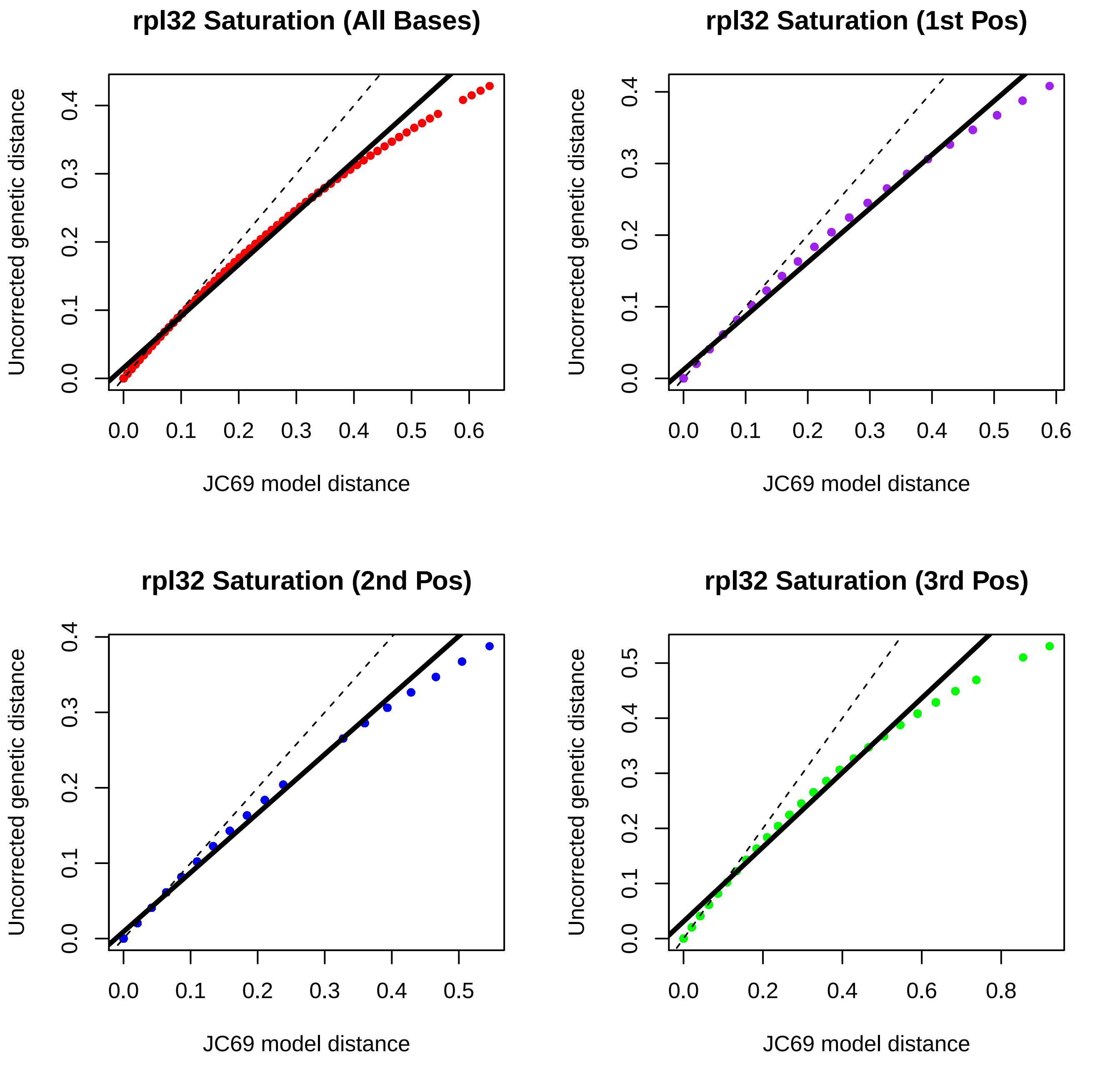

rpl32 Saturation (All Bases)
rpl32 Saturation (1st Pos)
rpl32 Saturation (2nd Pos)
rpl32 Saturation (3rd Pos)
Uncorrected genetic distance
JC69 model distance
0.0
0.1
0.2
0.3
0.4
0.5
0.6
0.8

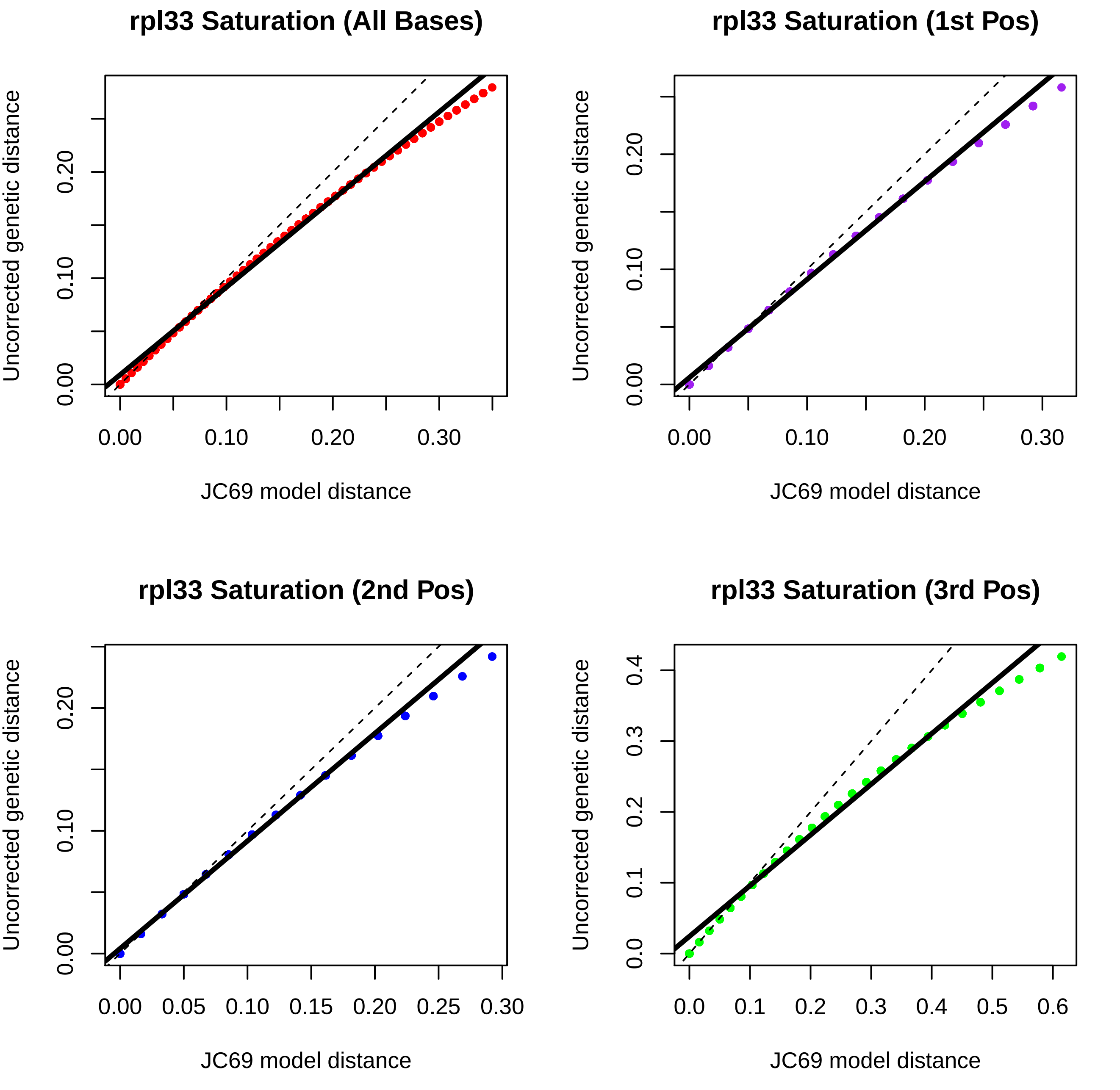
rpl33 Saturation (All Bases)
rpl33 Saturation (1st Pos)
rpl33 Saturation (2nd Pos)
rpl33 Saturation (3rd Pos)
Uncorrected genetic distance
JC69 model distance
0.00
0.10
0.20
0.30
0.05
0.15
0.25
0.0
0.1
0.2
0.3
0.4
0.5
0.6

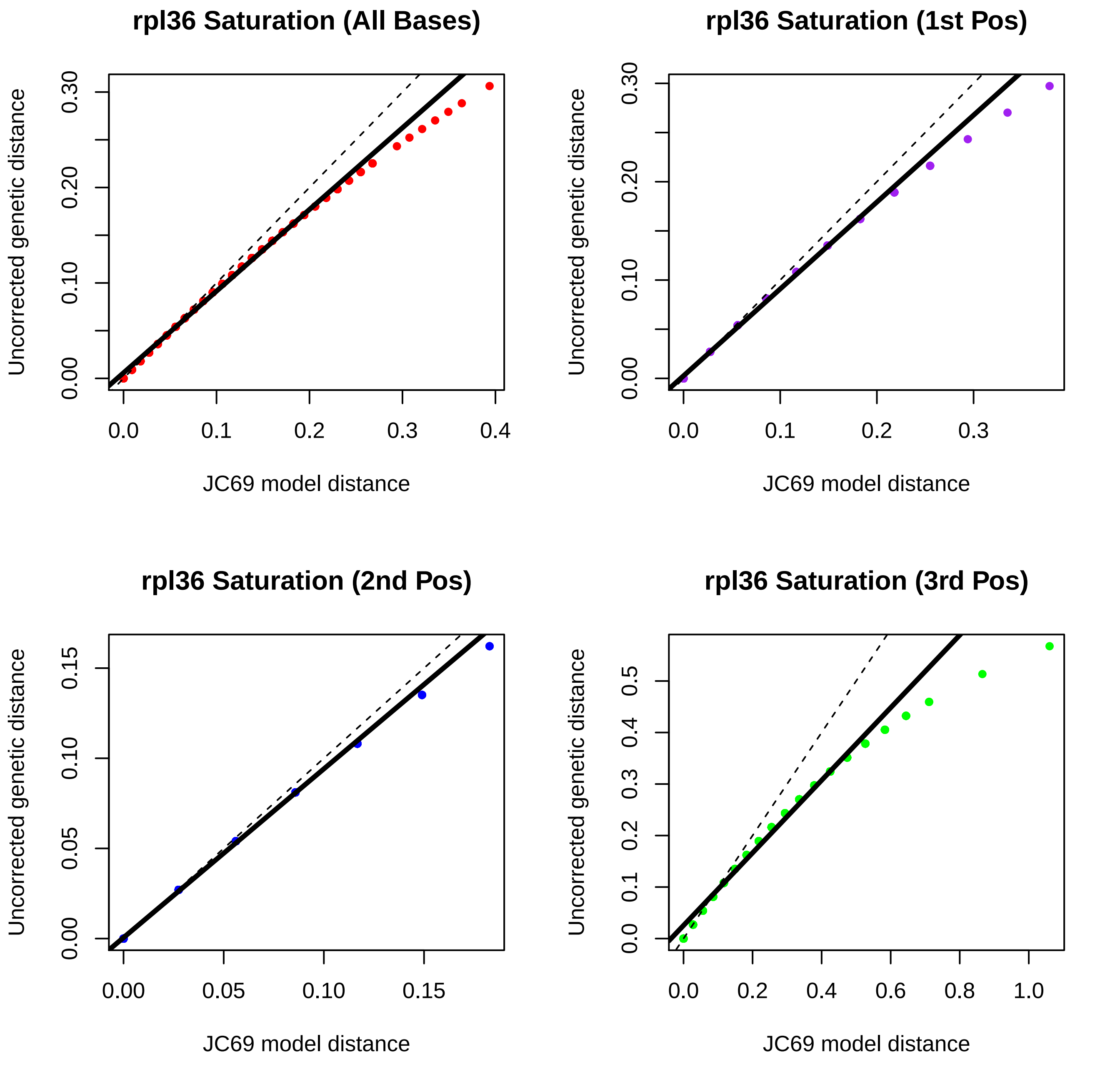

rpl36 Saturation (All Bases)
Uncorrected genetic distance
0.00
0.10
0.20
0.30
0.0
0.1
0.2
0.3
0.4
JC69 model distance
rpl36 Saturation (1st Pos)
Uncorrected genetic distance
0.00
0.10
0.20
0.30
0.0
0.1
0.2
0.3
JC69 model distance
rpl36 Saturation (2nd Pos)
Uncorrected genetic distance
0.00
0.05
0.10
0.15
0.00
0.05
0.10
0.15
JC69 model distance
rpl36 Saturation (3rd Pos)
Uncorrected genetic distance
0.0
0.1
0.2
0.3
0.4
0.5
0.0
0.2
0.4
0.6
0.8
1.0
JC69 model distance

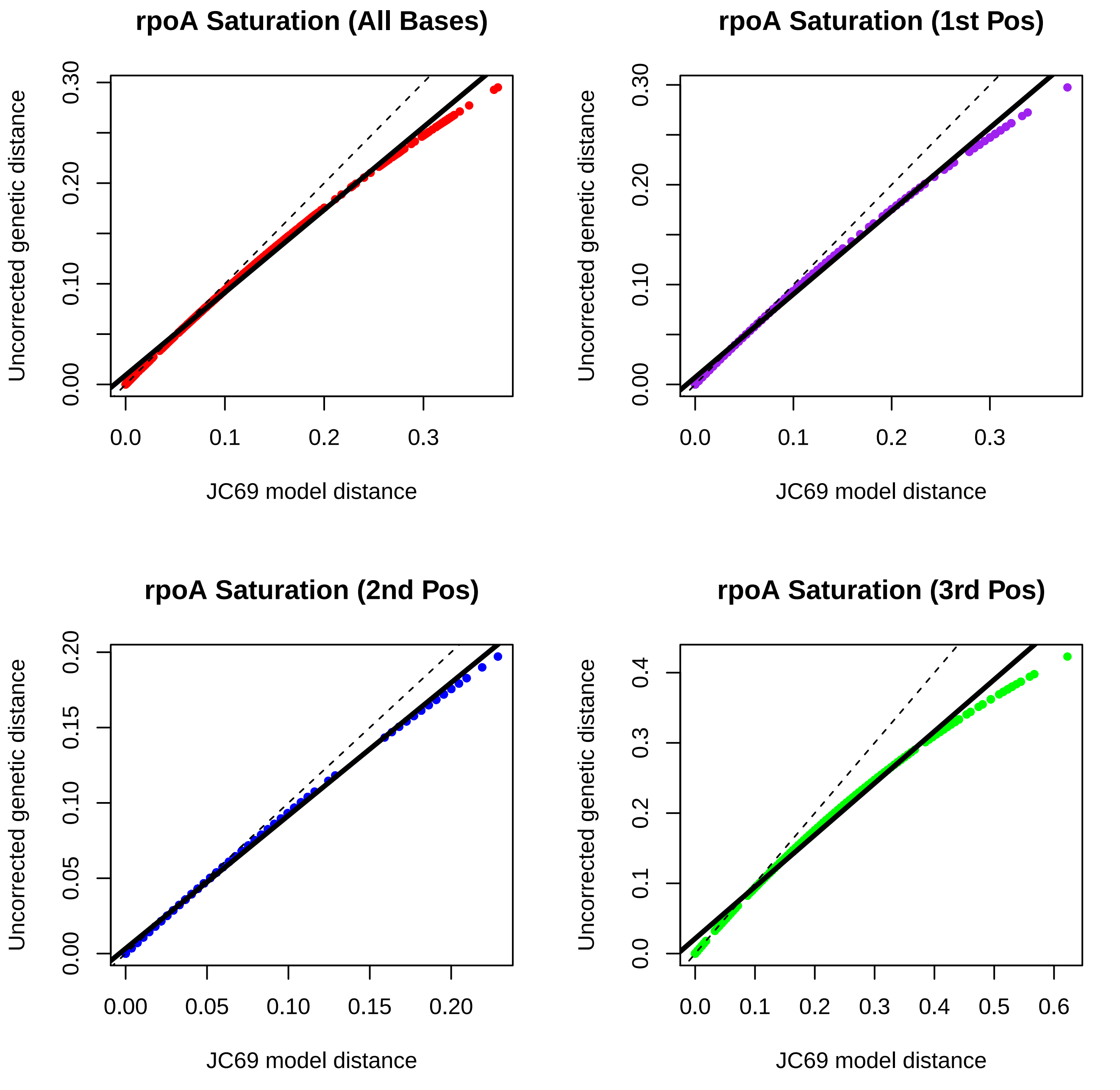
rpoA Saturation (All Bases)
Uncorrected genetic distance
0.00
0.10
0.20
0.30
0.0
0.1
0.2
0.3
JC69 model distance
rpoA Saturation (1st Pos)
Uncorrected genetic distance
0.00
0.10
0.20
0.30
0.0
0.1
0.2
0.3
JC69 model distance
rpoA Saturation (2nd Pos)
Uncorrected genetic distance
0.00
0.05
0.10
0.15
0.20
0.00
0.05
0.10
0.15
0.20
JC69 model distance
rpoA Saturation (3rd Pos)
Uncorrected genetic distance
0.0
0.1
0.2
0.3
0.4
0.0
0.1
0.2
0.3
0.4
0.5
0.6
JC69 model distance

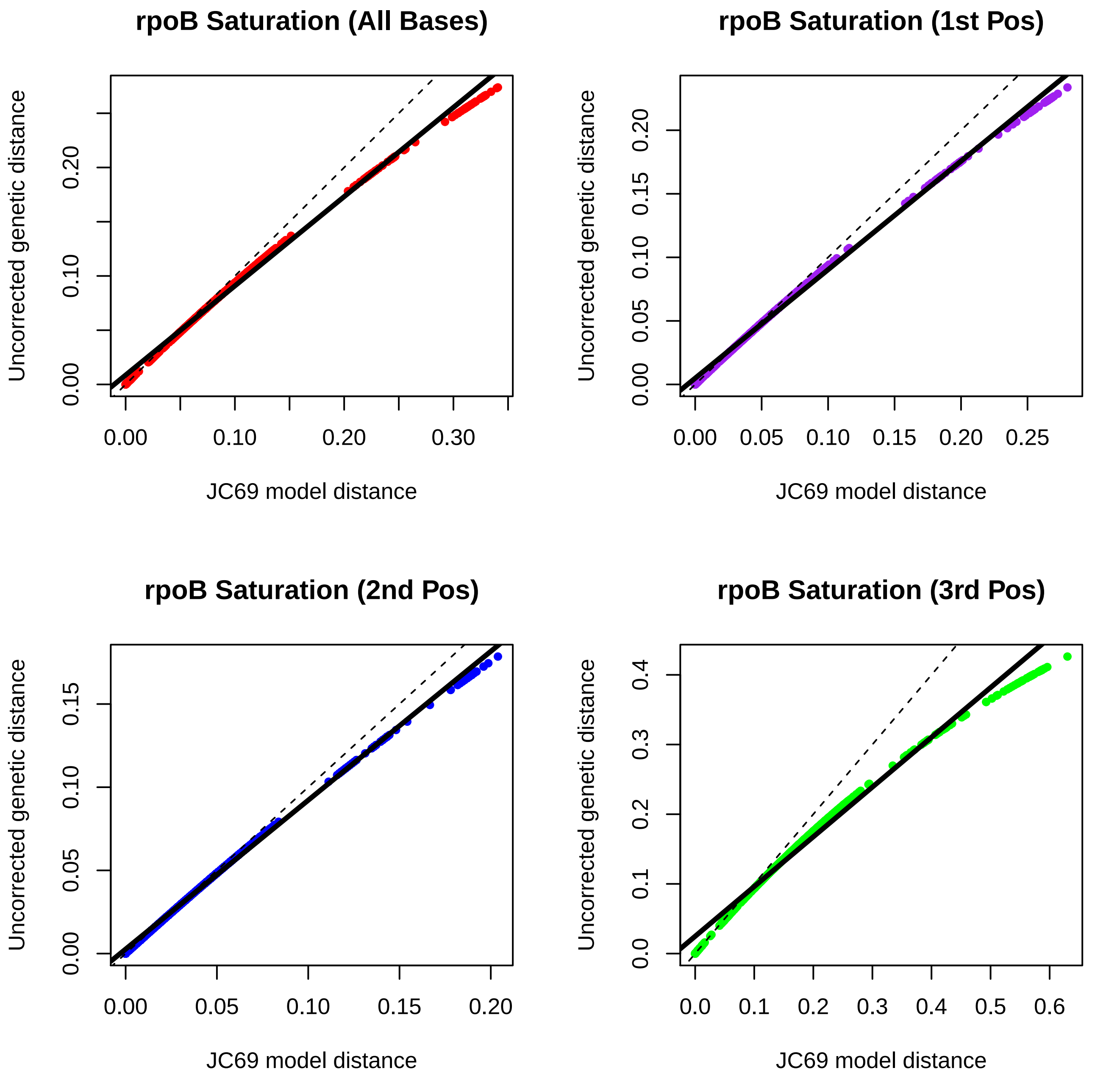

rpoB Saturation (All Bases)
Uncorrected genetic distance
0.00
0.10
0.20
JC69 model distance
0.00
0.10
0.20
0.30
rpoB Saturation (1st Pos)
Uncorrected genetic distance
0.00
0.05
0.10
0.15
0.20
JC69 model distance
0.00
0.05
0.10
0.15
0.20
0.25
rpoB Saturation (2nd Pos)
Uncorrected genetic distance
0.00
0.05
0.10
0.15
JC69 model distance
0.00
0.05
0.10
0.15
0.20
rpoB Saturation (3rd Pos)
Uncorrected genetic distance
0.0
0.1
0.2
0.3
0.4
JC69 model distance
0.0
0.1
0.2
0.3
0.4
0.5
0.6

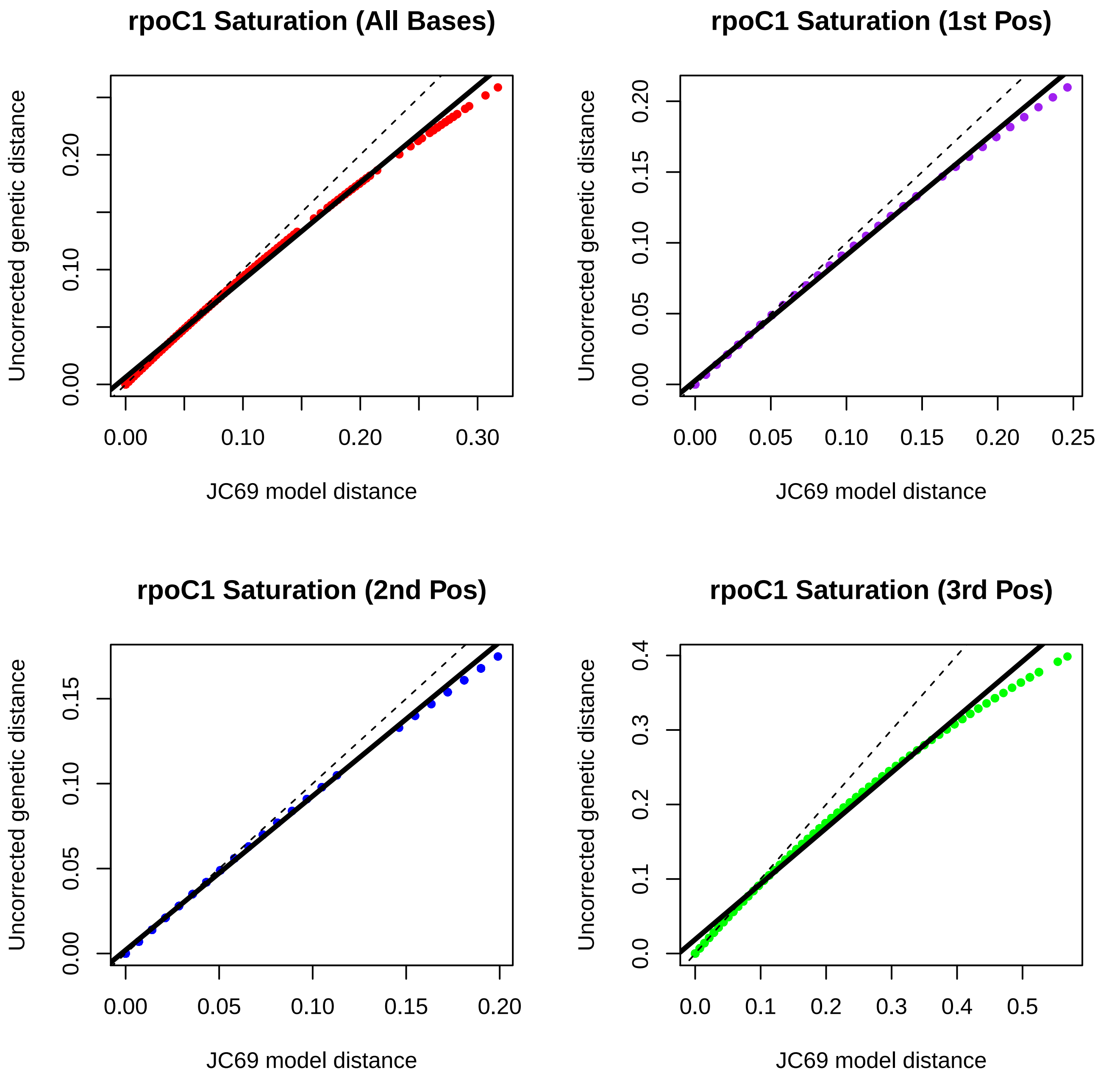

rpoC1 Saturation (All Bases)
Uncorrected genetic distance
0.00
0.10
0.20
0.00
0.10
0.20
0.30
JC69 model distance
rpoC1 Saturation (1st Pos)
Uncorrected genetic distance
0.00
0.05
0.10
0.15
0.20
0.00
0.05
0.10
0.15
0.20
0.25
JC69 model distance
rpoC1 Saturation (2nd Pos)
Uncorrected genetic distance
0.00
0.05
0.10
0.15
0.00
0.05
0.10
0.15
0.20
JC69 model distance
rpoC1 Saturation (3rd Pos)
Uncorrected genetic distance
0.0
0.1
0.2
0.3
0.4
0.0
0.1
0.2
0.3
0.4
0.5
JC69 model distance

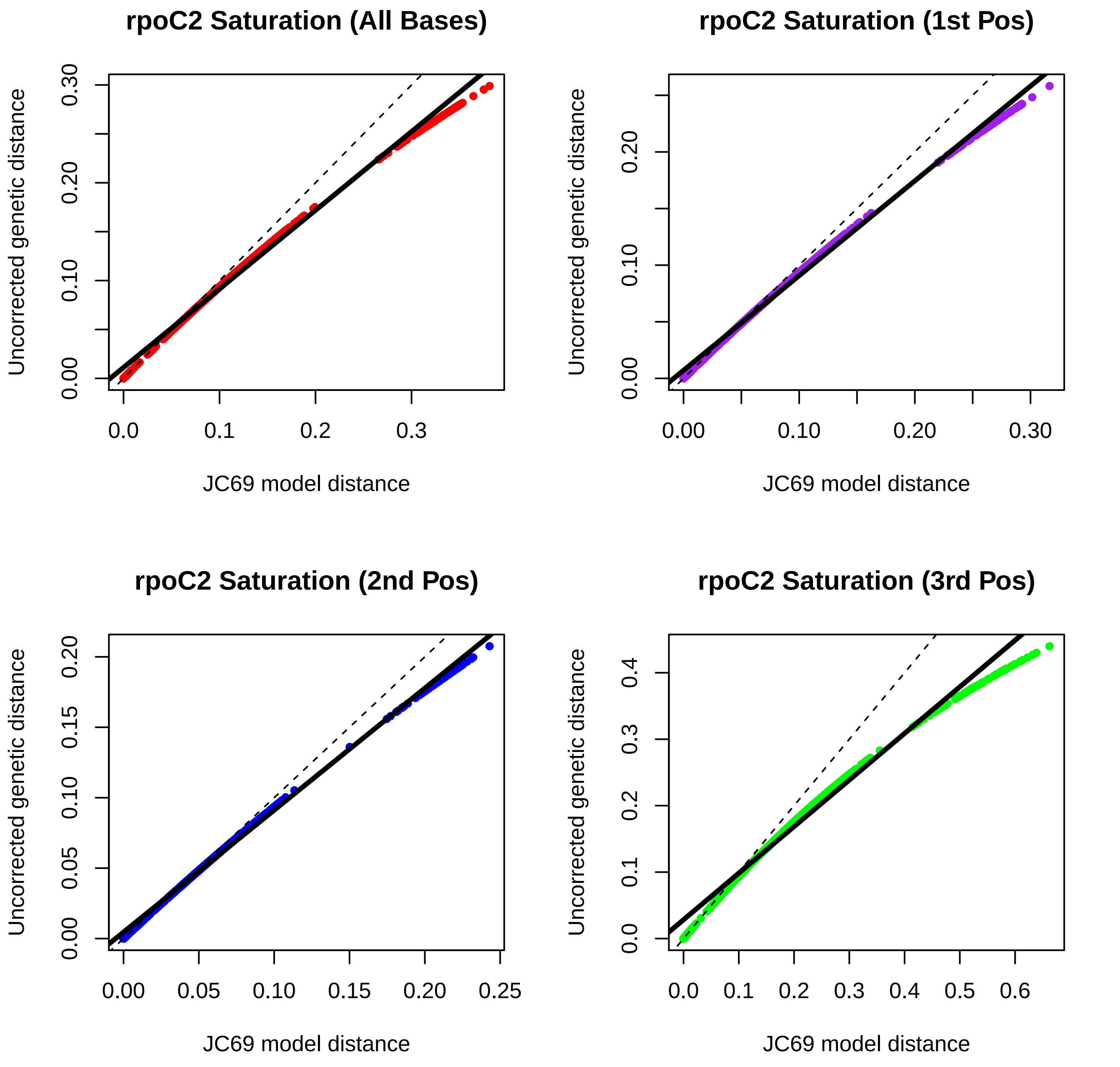

rpoC2 Saturation (All Bases)
Uncorrected genetic distance
0.00
0.10
0.20
0.30
0.0
0.1
0.2
0.3
JC69 model distance
rpoC2 Saturation (1st Pos)
Uncorrected genetic distance
0.00
0.10
0.20
0.00
0.10
0.20
0.30
JC69 model distance
rpoC2 Saturation (2nd Pos)
Uncorrected genetic distance
0.00
0.05
0.10
0.15
0.20
0.00
0.05
0.10
0.15
0.20
0.25
JC69 model distance
rpoC2 Saturation (3rd Pos)
Uncorrected genetic distance
0.0
0.1
0.2
0.3
0.4
0.0
0.1
0.2
0.3
0.4
0.5
0.6
JC69 model distance

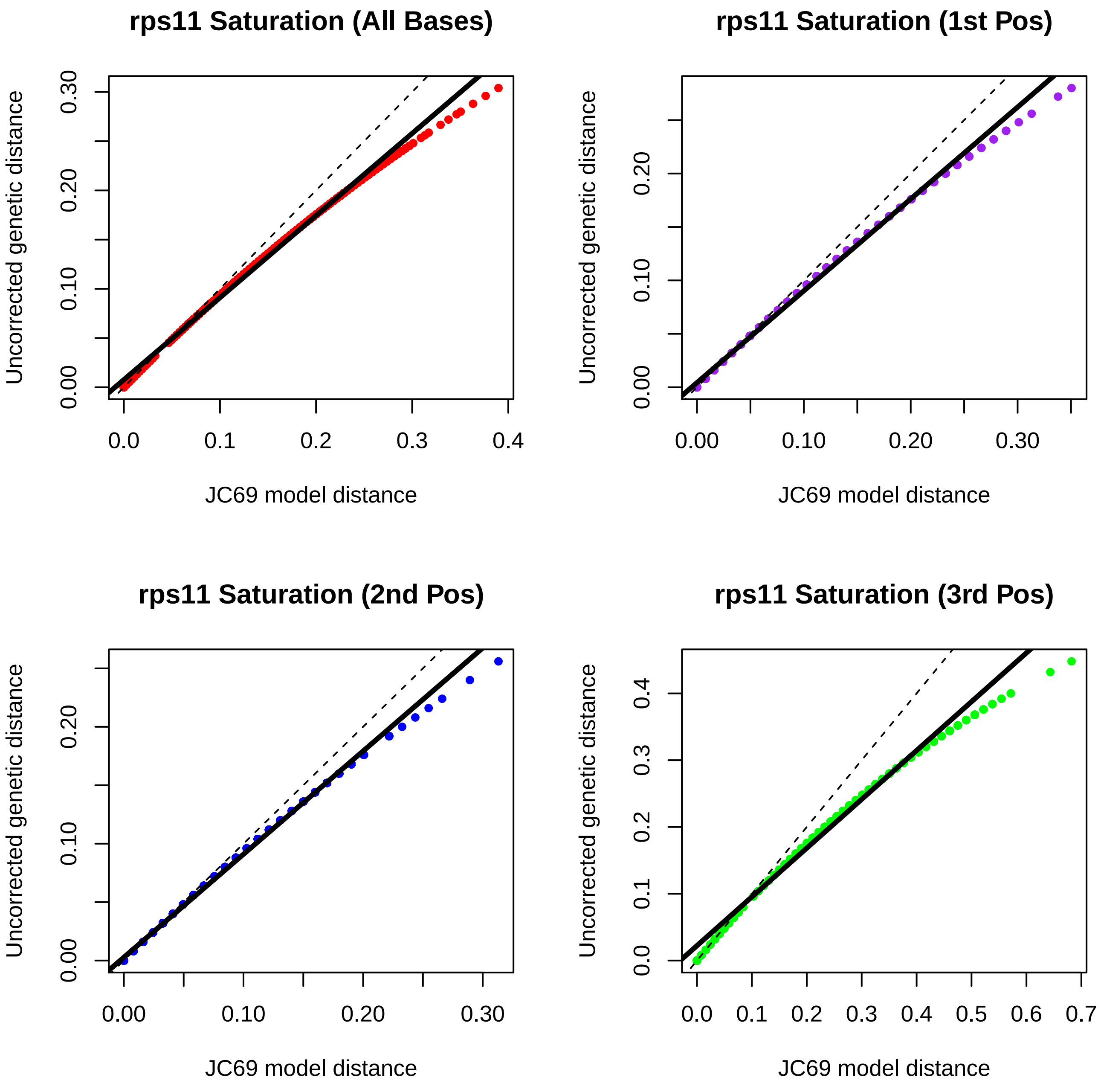
rps11 Saturation (All Bases)
Uncorrected genetic distance
0.00
0.10
0.20
0.30
0.0
0.1
0.2
0.3
0.4
JC69 model distance
rps11 Saturation (1st Pos)
Uncorrected genetic distance
0.00
0.10
0.20
0.00
0.10
0.20
0.30
JC69 model distance
rps11 Saturation (2nd Pos)
Uncorrected genetic distance
0.00
0.10
0.20
0.00
0.10
0.20
0.30
JC69 model distance
rps11 Saturation (3rd Pos)
Uncorrected genetic distance
0.0
0.1
0.2
0.3
0.4
0.0
0.1
0.2
0.3
0.4
0.5
0.6
0.7
JC69 model distance

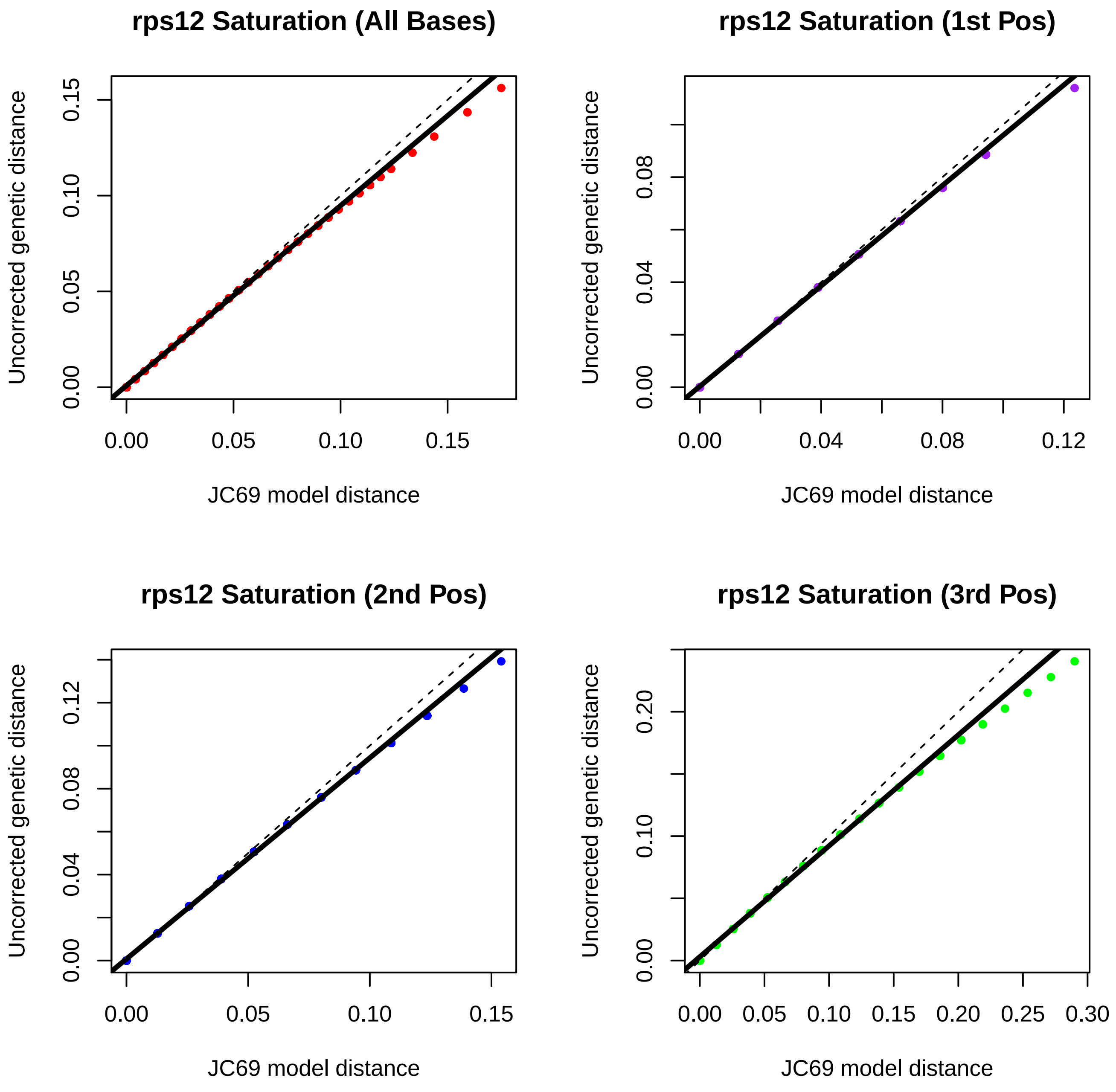

rps12 Saturation (All Bases)
rps12 Saturation (1st Pos)
rps12 Saturation (2nd Pos)
rps12 Saturation (3rd Pos)
Uncorrected genetic distance
JC69 model distance
0.00
0.05
0.10
0.15
0.04
0.08
0.12
0.20
0.25
0.30

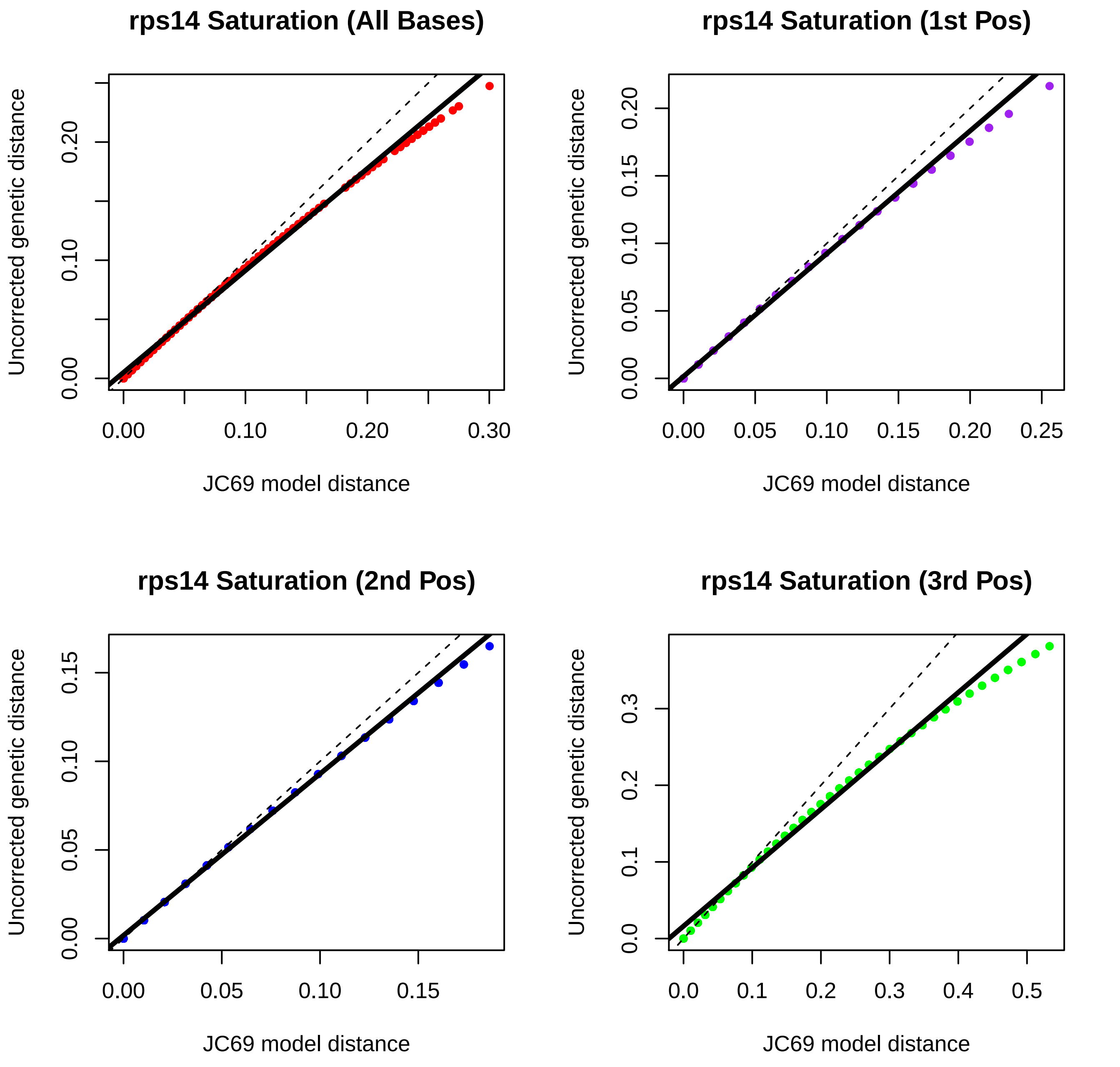
rps14 Saturation (All Bases)
rps14 Saturation (1st Pos)
rps14 Saturation (2nd Pos)
rps14 Saturation (3rd Pos)
Uncorrected genetic distance
JC69 model distance

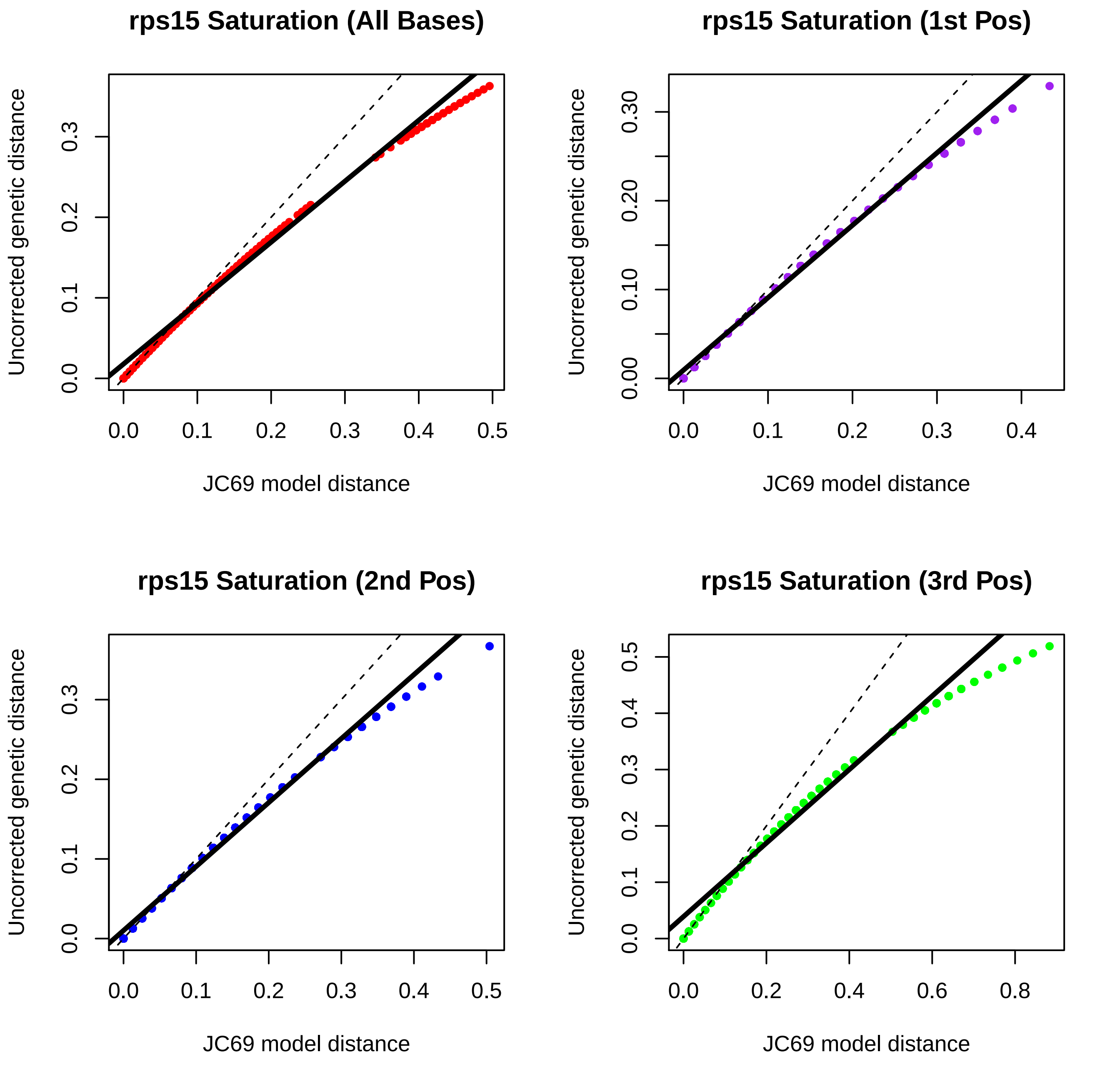
rps15 Saturation (All Bases)
rps15 Saturation (1st Pos)
rps15 Saturation (2nd Pos)
rps15 Saturation (3rd Pos)
Uncorrected genetic distance
JC69 model distance
0.0
0.1
0.2
0.3
0.4
0.5
0.00
0.10
0.20
0.30
0.6
0.8

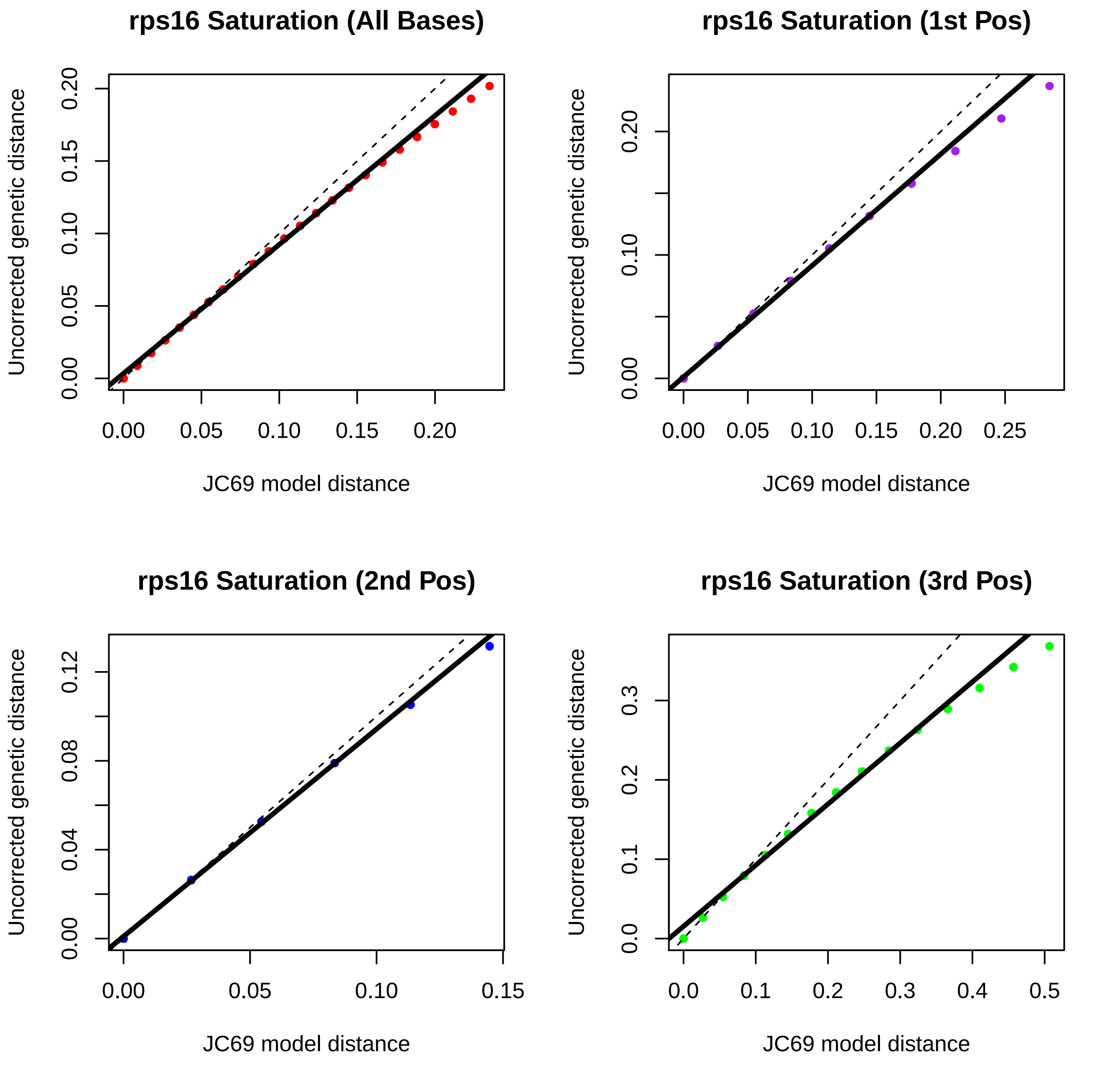
rps16 Saturation (All Bases)
Uncorrected genetic distance
0.00
0.05
0.10
0.15
0.20
JC69 model distance
rps16 Saturation (1st Pos)
Uncorrected genetic distance
0.00
0.10
0.20
0.25
JC69 model distance
0.15
0.05
rps16 Saturation (2nd Pos)
Uncorrected genetic distance
0.00
0.04
0.08
0.12
0.05
0.10
0.15
JC69 model distance
rps16 Saturation (3rd Pos)
Uncorrected genetic distance
0.0
0.1
0.2
0.3
0.4
0.5
JC69 model distance

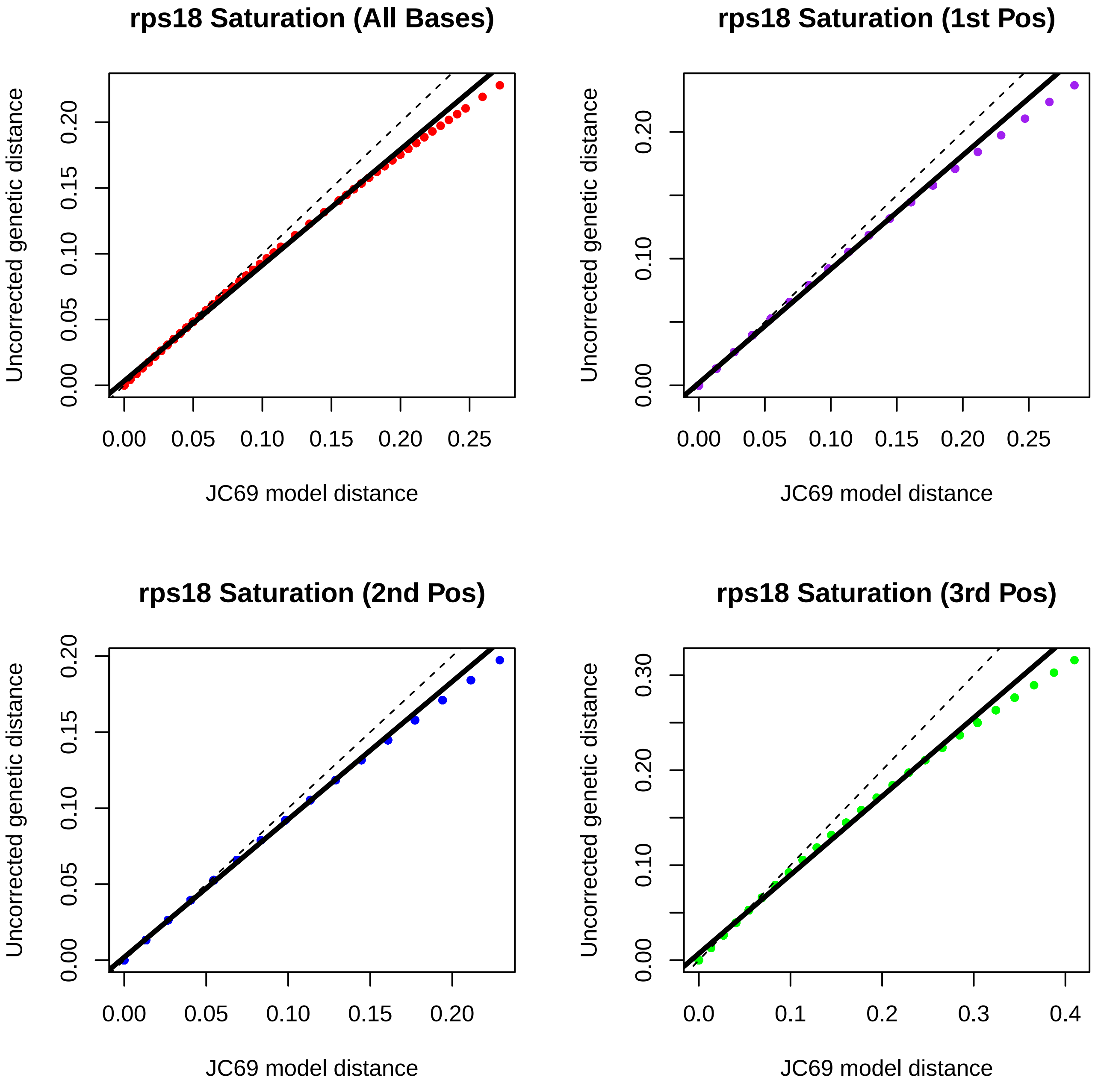
rps18 Saturation (All Bases)
rps18 Saturation (1st Pos)
rps18 Saturation (2nd Pos)
rps18 Saturation (3rd Pos)
Uncorrected genetic distance
JC69 model distance
0.00
0.05
0.10
0.15
0.20
0.25
0.30
0.0
0.1
0.2
0.3
0.4

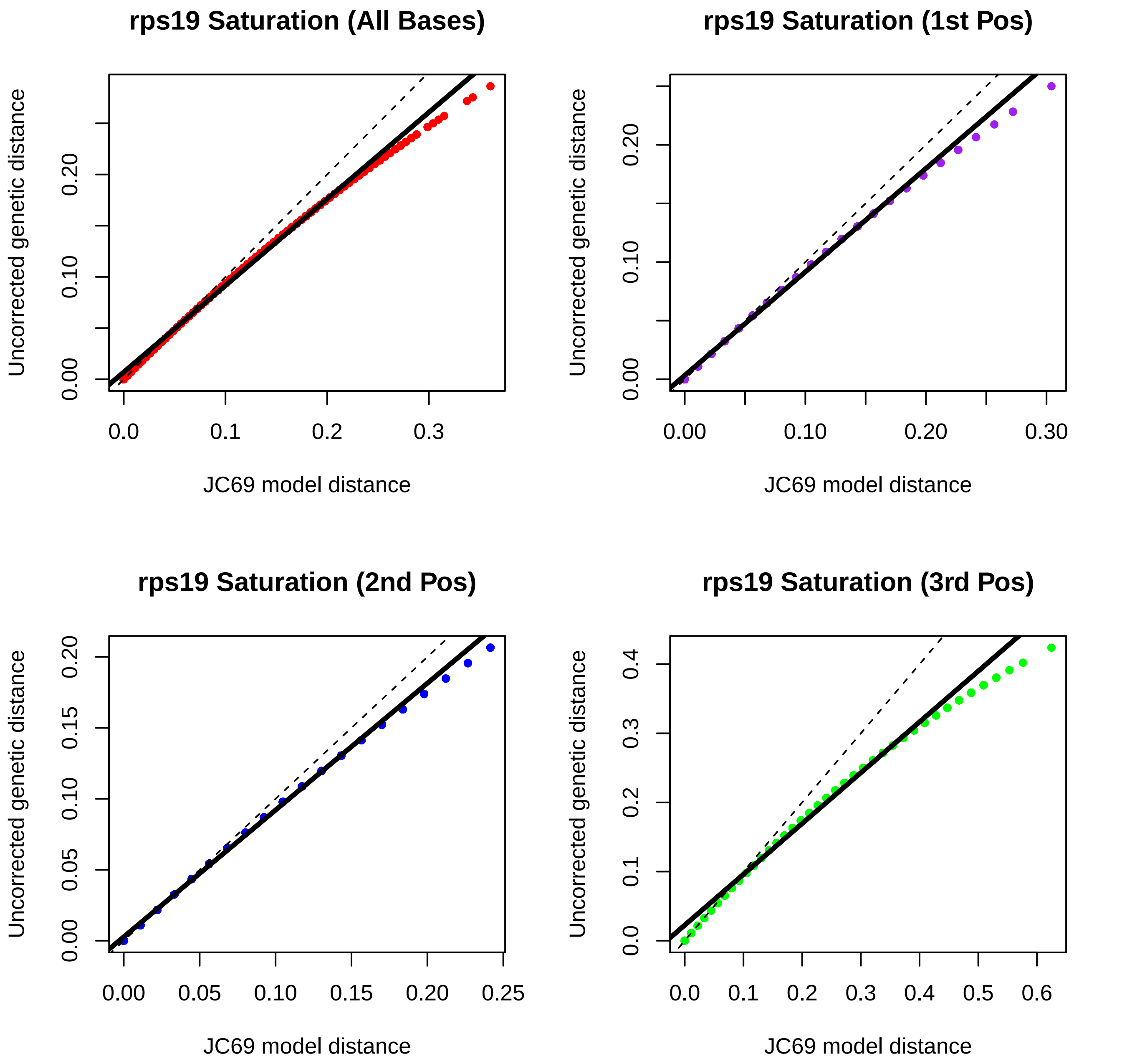

rps19 Saturation (All Bases)
Uncorrected genetic distance
0.00
0.10
0.20
0.0
0.1
0.2
0.3
JC69 model distance
rps19 Saturation (1st Pos)
Uncorrected genetic distance
0.00
0.10
0.20
0.00
0.10
0.20
0.30
JC69 model distance
rps19 Saturation (2nd Pos)
Uncorrected genetic distance
0.00
0.05
0.10
0.15
0.20
0.00
0.05
0.10
0.15
0.20
0.25
JC69 model distance
rps19 Saturation (3rd Pos)
Uncorrected genetic distance
0.0
0.1
0.2
0.3
0.4
0.0
0.1
0.2
0.3
0.4
0.5
0.6
JC69 model distance

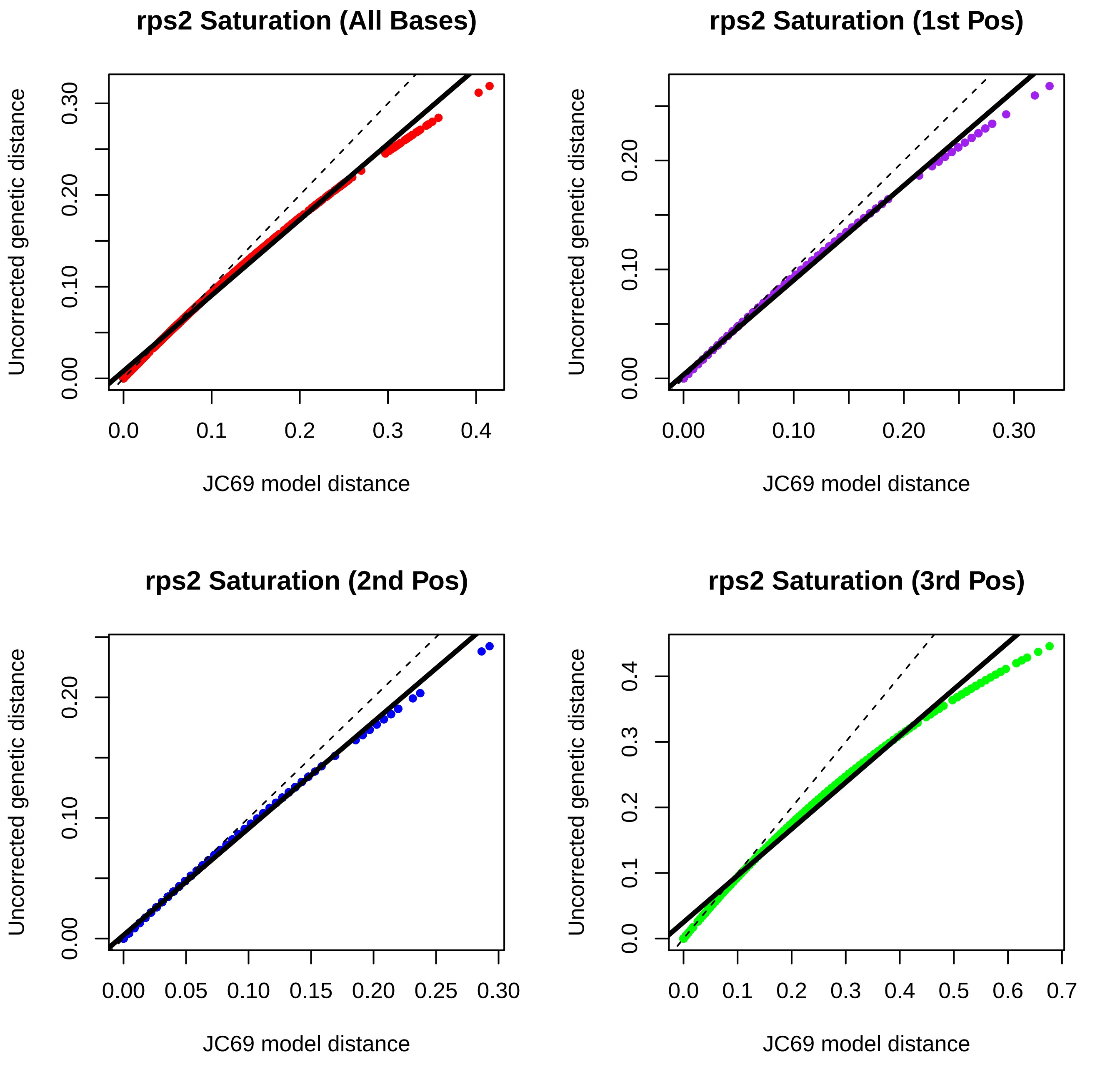
rps2 Saturation (All Bases)
Uncorrected genetic distance
0.00
0.10
0.20
0.30
0.0
0.1
0.2
0.3
0.4
JC69 model distance
rps2 Saturation (1st Pos)
Uncorrected genetic distance
0.00
0.10
0.20
0.00
0.10
0.20
0.30
JC69 model distance
rps2 Saturation (2nd Pos)
Uncorrected genetic distance
0.00
0.10
0.20
0.00
0.05
0.10
0.15
0.20
0.25
0.30
JC69 model distance
rps2 Saturation (3rd Pos)
Uncorrected genetic distance
0.0
0.1
0.2
0.3
0.4
0.0
0.1
0.2
0.3
0.4
0.5
0.6
0.7
JC69 model distance

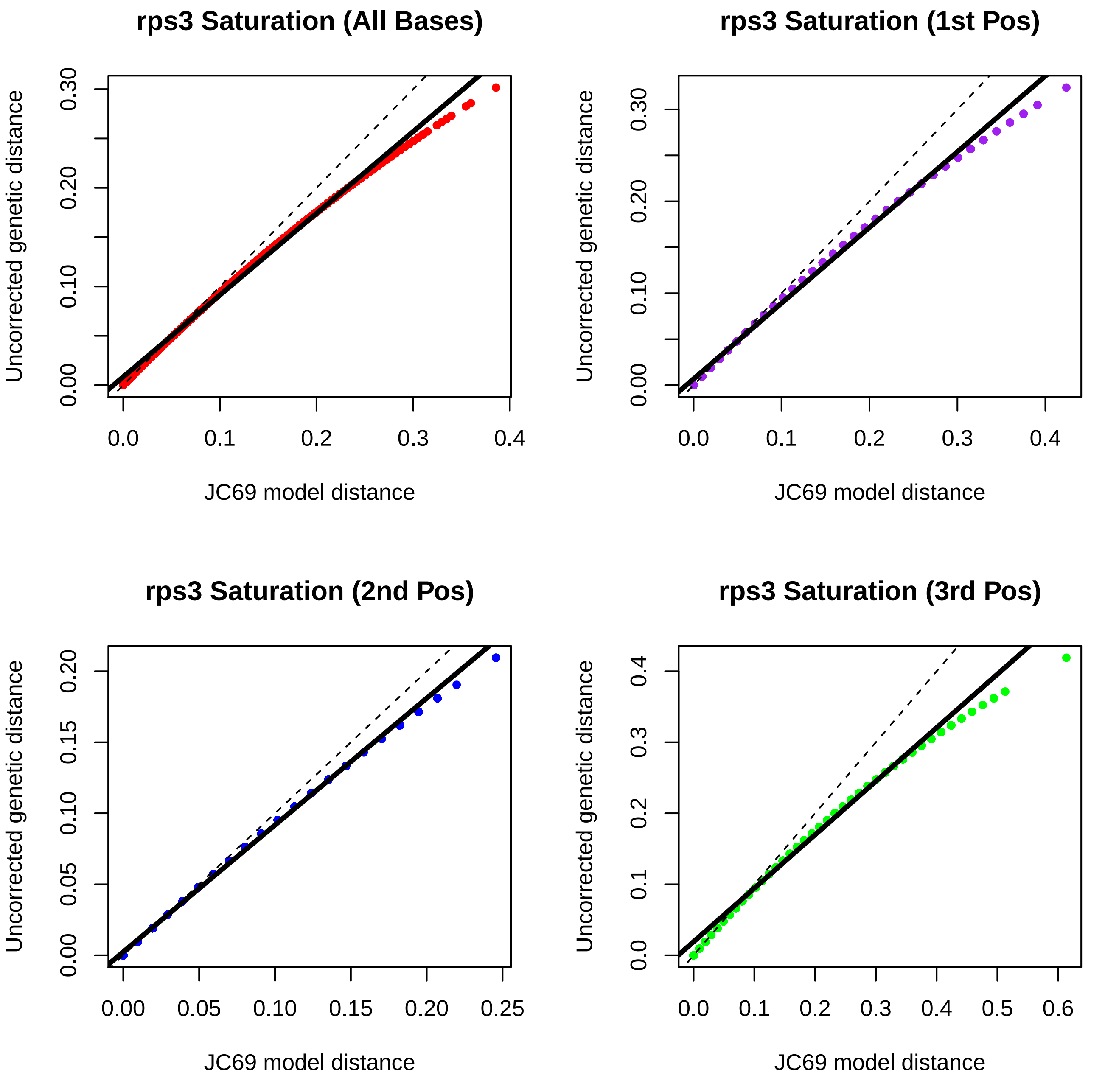
rps3 Saturation (All Bases)
rps3 Saturation (1st Pos)
rps3 Saturation (2nd Pos)
rps3 Saturation (3rd Pos)
Uncorrected genetic distance
JC69 model distance
0.00
0.10
0.20
0.30
0.0
0.1
0.2
0.3
0.4
0.05
0.15
0.25
0.5
0.6

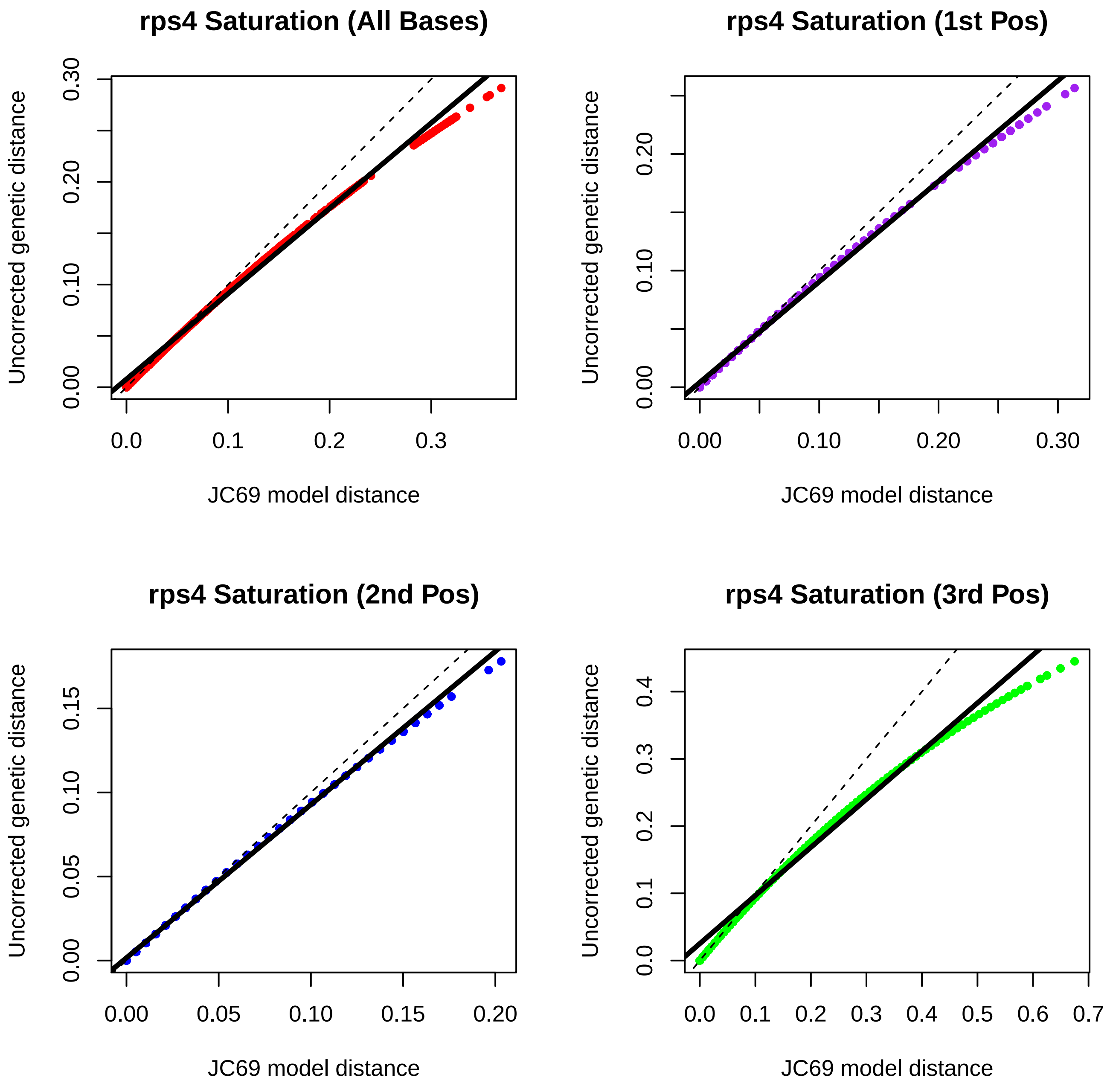
rps4 Saturation (All Bases)
rps4 Saturation (1st Pos)
rps4 Saturation (2nd Pos)
rps4 Saturation (3rd Pos)
Uncorrected genetic distance
JC69 model distance
0.00
0.10
0.20
0.30
0.0
0.1
0.2
0.3
0.05
0.15
0.4
0.5
0.6
0.7

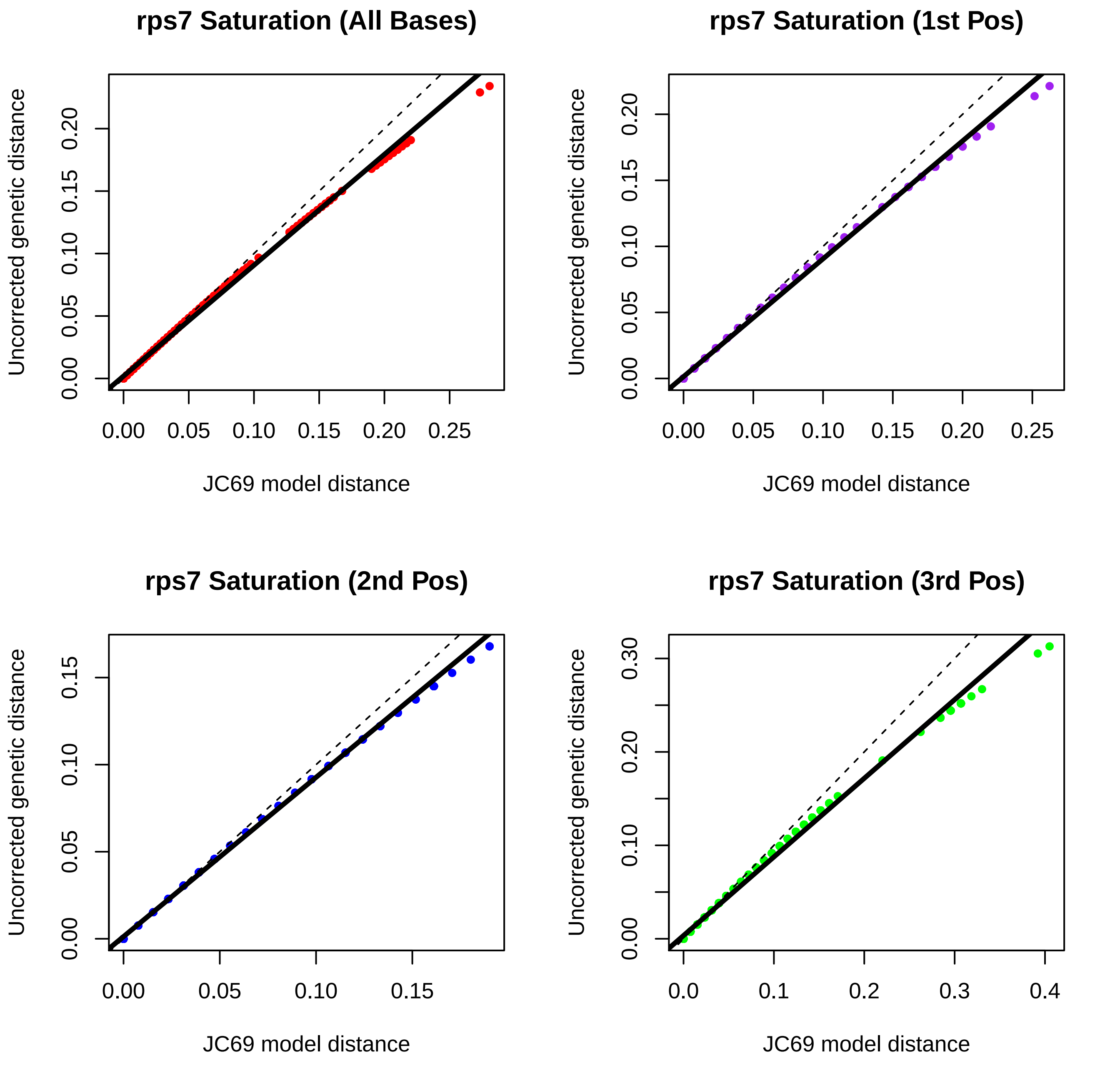
rps7 Saturation (All Bases)
rps7 Saturation (1st Pos)
rps7 Saturation (2nd Pos)
rps7 Saturation (3rd Pos)
Uncorrected genetic distance
JC69 model distance
0.00
0.05
0.10
0.15
0.20
0.25
0.0
0.1
0.2
0.3
0.4

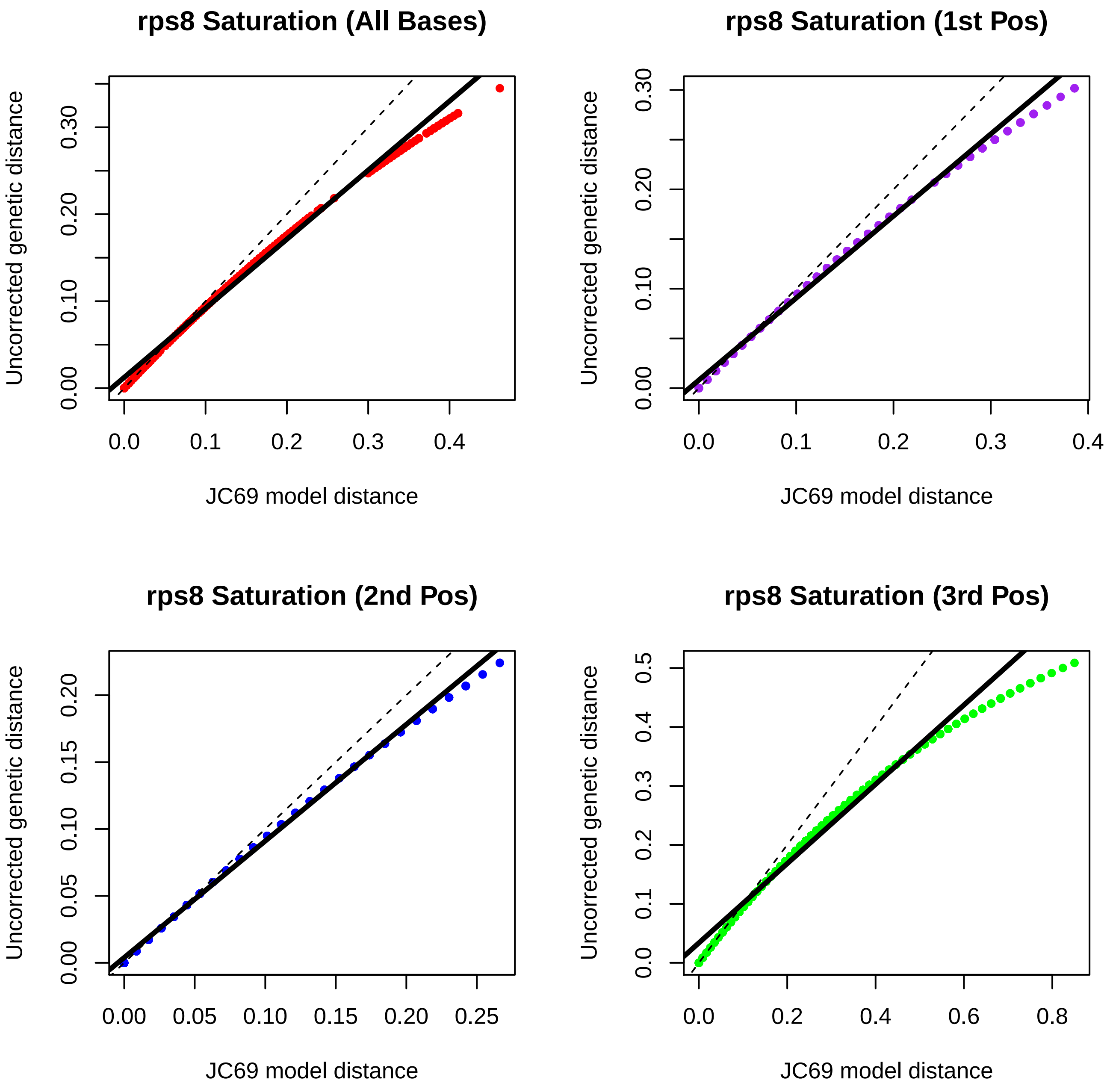
rps8 Saturation (All Bases)
rps8 Saturation (1st Pos)
rps8 Saturation (2nd Pos)
rps8 Saturation (3rd Pos)
Uncorrected genetic distance
JC69 model distance

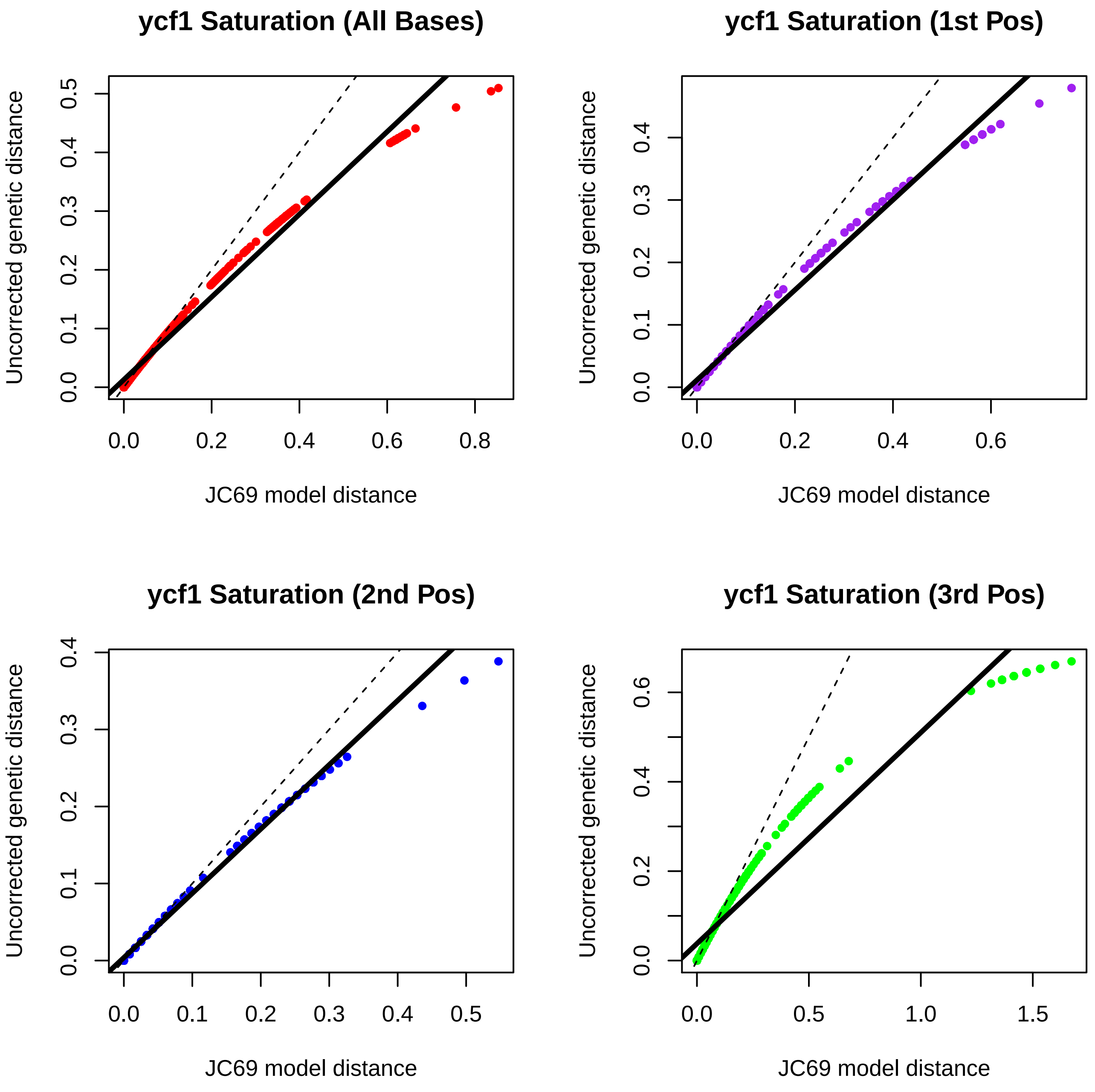
ycf1 Saturation (All Bases)
Uncorrected genetic distance
0.0
0.1
0.2
0.3
0.4
0.5
0.0
0.2
0.4
0.6
0.8
JC69 model distance
ycf1 Saturation (1st Pos)
Uncorrected genetic distance
0.0
0.1
0.2
0.3
0.4
0.0
0.2
0.4
0.6
JC69 model distance
ycf1 Saturation (2nd Pos)
Uncorrected genetic distance
0.0
0.1
0.2
0.3
0.4
0.0
0.1
0.2
0.3
0.4
0.5
JC69 model distance
ycf1 Saturation (3rd Pos)
Uncorrected genetic distance
0.0
0.2
0.4
0.6
0.0
0.5
1.0
1.5
JC69 model distance

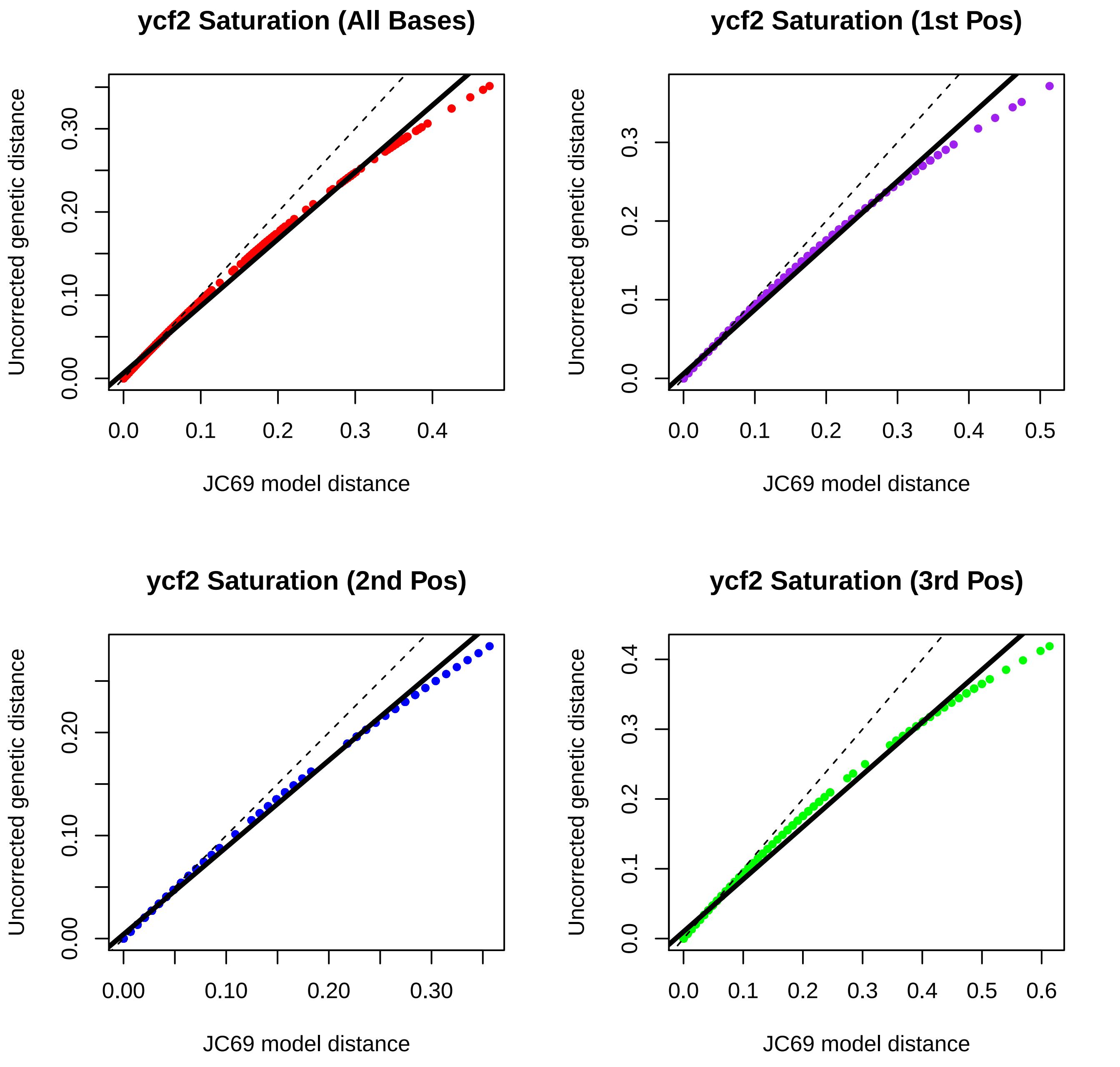
ycf2 Saturation (All Bases)
Uncorrected genetic distance
JC69 model distance
0.00
0.10
0.20
0.30
0.0
0.1
0.2
0.3
0.4
ycf2 Saturation (1st Pos)
Uncorrected genetic distance
JC69 model distance
0.0
0.1
0.2
0.3
0.0
0.1
0.2
0.3
0.4
0.5
ycf2 Saturation (2nd Pos)
Uncorrected genetic distance
JC69 model distance
0.00
0.10
0.20
0.00
0.10
0.20
0.30
ycf2 Saturation (3rd Pos)
Uncorrected genetic distance
JC69 model distance
0.0
0.1
0.2
0.3
0.4
0.0
0.1
0.2
0.3
0.4
0.5
0.6

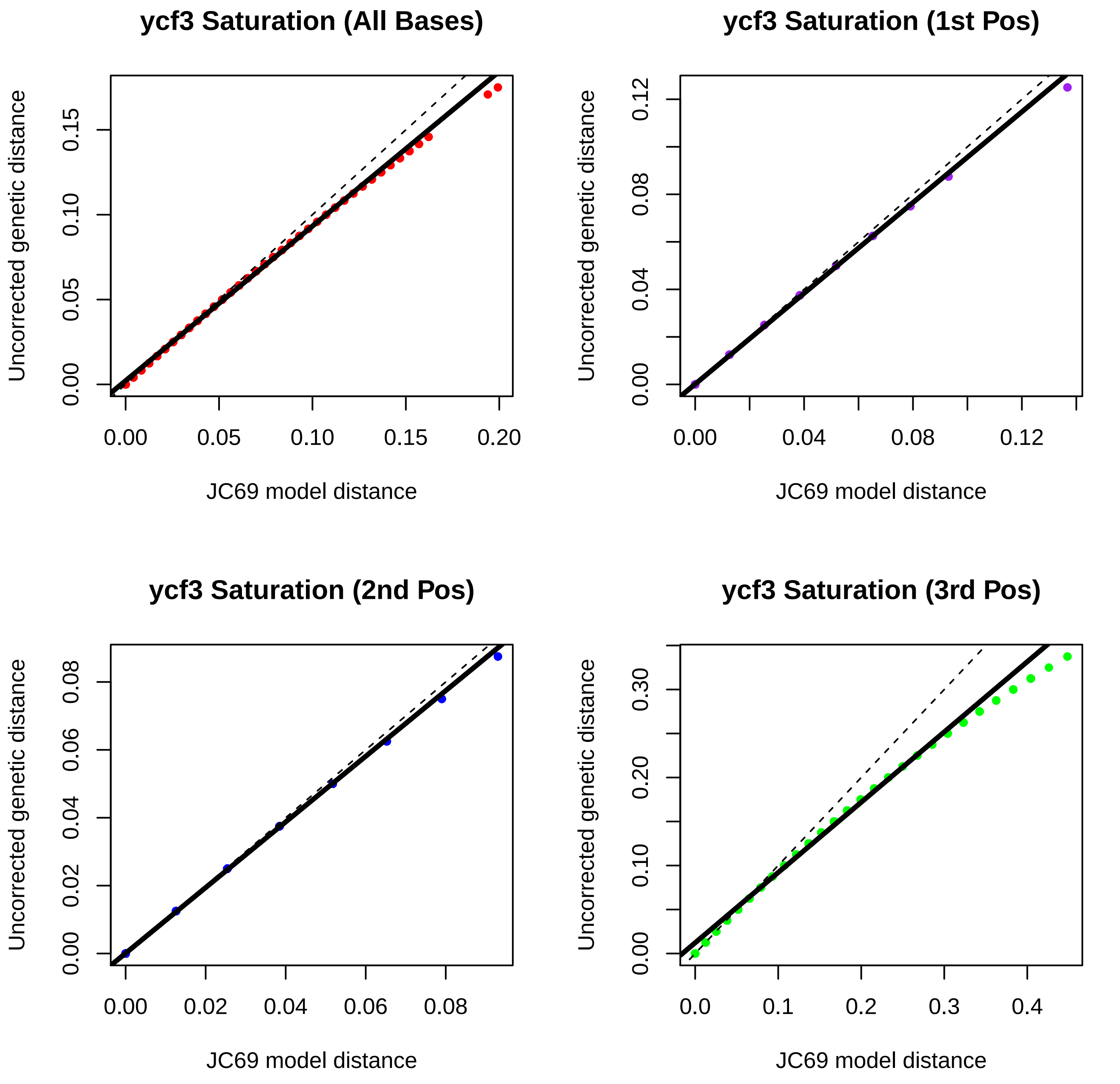
ycf3 Saturation (All Bases)
ycf3 Saturation (1st Pos)
ycf3 Saturation (2nd Pos)
ycf3 Saturation (3rd Pos)
Uncorrected genetic distance
JC69 model distance
0.00
0.05
0.10
0.15
0.20
0.04
0.08
0.12
0.02
0.06
0.0
0.1
0.2
0.3
0.4
0.30

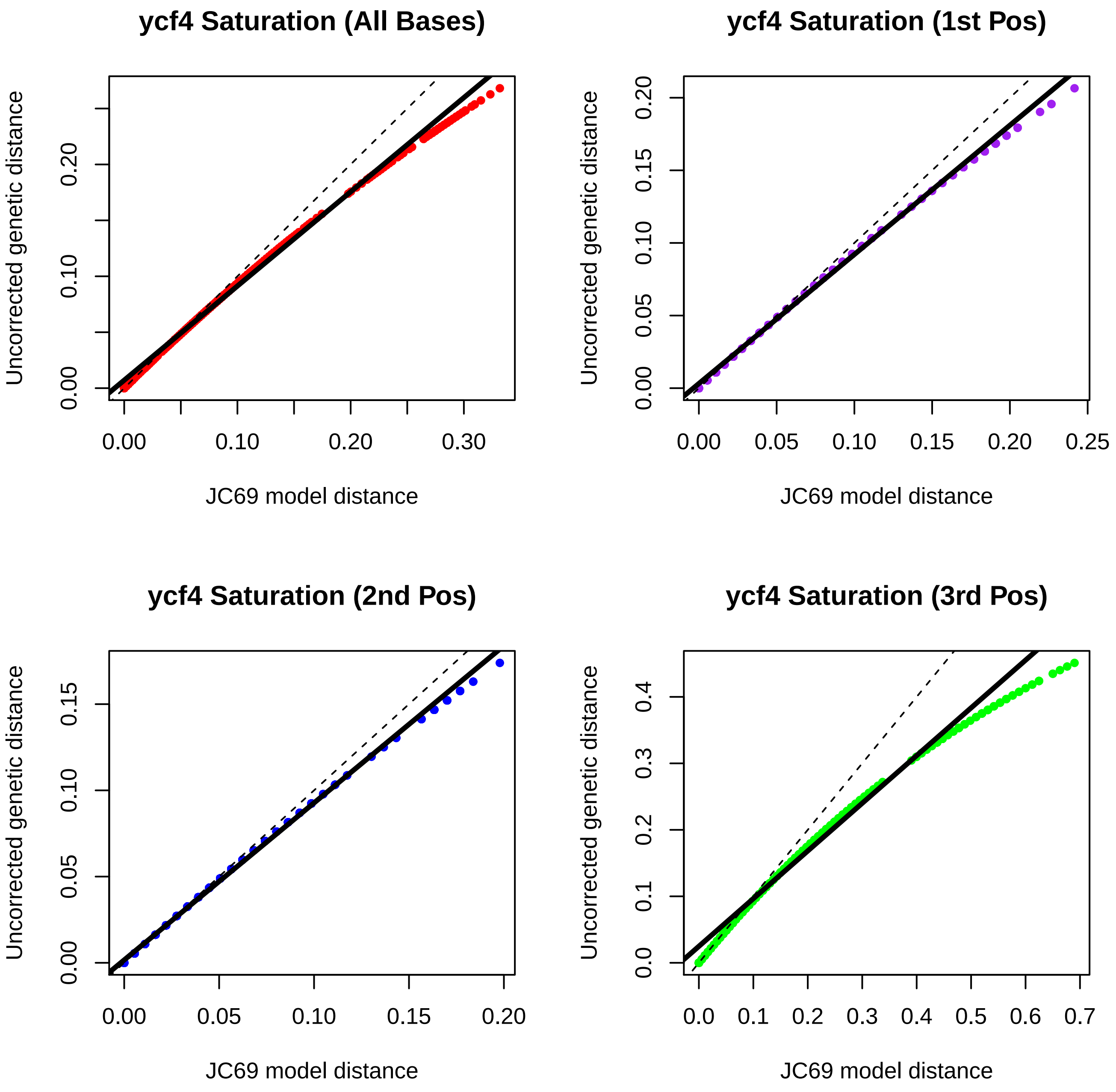

ycf4 Saturation (All Bases)
Uncorrected genetic distance
0.00
0.10
0.20
0.00
0.10
0.20
0.30
JC69 model distance
ycf4 Saturation (1st Pos)
Uncorrected genetic distance
0.00
0.05
0.10
0.15
0.20
0.00
0.05
0.10
0.15
0.20
0.25
JC69 model distance
ycf4 Saturation (2nd Pos)
Uncorrected genetic distance
0.00
0.05
0.10
0.15
0.00
0.05
0.10
0.15
0.20
JC69 model distance
ycf4 Saturation (3rd Pos)
Uncorrected genetic distance
0.0
0.1
0.2
0.3
0.4
0.0
0.1
0.2
0.3
0.4
0.5
0.6
0.7
JC69 model distance
