# Supplementary material for: Characterizing gene tree conflict in plastome-inferred phylogenies
Source: PeerJ. 2019 Sep 24;7:e7747. doi: 10.7717/peerj.7747 (PMC6764362; doi:10.7717/peerj.7747)
Supplement: Table S2 — Binomial logistic regression results for all genes from a range of datasets, including all predictors, both ignoring and considering BS ≥ 70% support. Parameters are not transformed i.e., they represent the estimated ceteris paribus effect of the predictor on log odds. Quantities in brackets are standard errors. [file peerj-07-7747-s007.pdf]

Table S2: Binomial logistic regression results for all genes from a range of datasets, including all predictors, both ignoring and considering BS ≥ 70% support. Parameters are not transformed i.e. they represent the estimated ceteris paribus effect of the predictor on log odds. Quantities in brackets are standard errors.

	Dependent variable: Total Concordant/Total Discordant									
	FSA cln AA	FSA cln AA BS ≥ 70	FSA cln nuc	FSA cln nuc BS ≥ 70	MAFFT AA	MAFFT AA BS ≥ 70	MAFFT nuc	MAFFT nuc BS ≥ 70	WAG	WAG BS ≥ 70
alignment_length	0.001^{***}	0.001^{***}	0.001^{***}	0.001^{***}	0.001^{***}	0.001^{***}	0.0003^{***}	0.0004^{***}	0.0002^{***}	0.0002^{***}
	(0.0001)	(0.0001)	(0.00004)	(0.00004)	(0.0001)	(0.0001)	(0.00003)	(0.00003)	(0.00004)	(0.00004)
tree_length	0.539^{***}	0.746^{***}	1.124^{***}	1.350^{***}	0.680^{***}	0.702^{***}	0.925^{***}	1.390^{***}	0.616^{***}	0.736^{***}
	(0.049)	(0.070)	(0.103)	(0.122)	(0.042)	(0.049)	(0.085)	(0.115)	(0.051)	(0.075)
variance	-39.588^{***}	-106.125^{***}	-245.838^{***}	-374.209^{***}	-62.247^{***}	-63.180^{***}	-145.851^{***}	-371.749^{***}	-53.085^{***}	-99.388^{***}
	(7.572)	(13.084)	(32.927)	(38.174)	(3.966)	(4.486)	(17.464)	(33.871)	(8.321)	(15.877)
Constant	-2.238^{***}	-3.261^{***}	-1.738^{***}	-2.852^{***}	-2.427^{***}	-3.468^{***}	-1.489^{***}	-2.721^{***}	-2.062^{***}	-2.982^{***}
	(0.073)	(0.102)	(0.102)	(0.129)	(0.077)	(0.107)	(0.095)	(0.124)	(0.070)	(0.096)
Observations	79	79	79	79	79	79	79	79	79	79
Log Likelihood	-319.263	-201.664	-283.221	-246.157	-281.726	-184.137	-348.941	-280.545	-345.767	-233.138
Akaike Inf. Crit.	646.526	411.327	574.443	500.313	571.451	376.274	705.883	569.090	699.535	474.275
Note:	*p<0.1; **p<0.05; ***p<0.01									
