# Supplementary material for: Characterizing gene tree conflict in plastome-inferred phylogenies
Source: PeerJ. 2019 Sep 24;7:e7747. doi: 10.7717/peerj.7747 (PMC6764362; doi:10.7717/peerj.7747)
Supplement: Table S3 — Quasibinomial logistic regression results for all genes from a range of datasets, including all predictors, both ignoring and considering BS ≥ 70% support. Parameters are not transformed i.e. they represent the estimated ceteris paribus effect of the predictor on log odds. Quantities in brackets are standard errors. Dispersion gives the estimated quasibinomial dispersion parameter. [file peerj-07-7747-s008.pdf]

Table S3: Quasibinomial logistic regression results for all genes from a range of datasets, including all predictors, both ignoring and considering BS ≥ 70% support. Parameters are not transformed i.e. they represent the estimated ceteris paribus effect of the predictor on log odds. Quantities in brackets are standard errors. Dispersion gives the estimated quasibinomial dispersion parameter.

	Dependent variable: Total Concordant/Total Discordant									
	FSA cln AA	FSA cln AA BS ≥ 70	FSA cln nuc	FSA cln nuc BS ≥ 70	MAFFT AA	MAFFT AA BS ≥ 70	MAFFT nuc	MAFFT nuc BS ≥ 70	WAG	WAG BS ≥ 70
alignment_length	0.001^{***}	0.001	0.001^{***}	0.001^{***}	0.001^{***}	0.001^{***}	0.0003^{***}	0.0004^{***}	0.0002^{**}	0.0002^{**}
	(0.0003)	(0.001)	(0.0001)	(0.0001)	(0.0002)	(0.0001)	(0.0001)	(0.0001)	(0.0001)	(0.0001)
tree_length	0.539^{***}	0.746	1.124^{***}	1.350^{***}	0.680^{***}	0.702^{***}	0.925^{***}	1.390^{***}	0.616^{***}	0.736^{***}
	(0.122)	(0.709)	(0.185)	(0.199)	(0.085)	(0.066)	(0.193)	(0.256)	(0.122)	(0.169)
variance	-39.588^{**}	-106.125	-245.838^{***}	-374.209^{***}	-62.247^{***}	-63.180^{***}	-145.851^{***}	-371.749^{***}	-53.085^{***}	-99.388^{***}
	(18.726)	(133.489)	(59.407)	(62.058)	(7.986)	(6.008)	(39.803)	(75.183)	(19.897)	(35.646)
Constant	-2.238^{***}	-3.261^{***}	-1.738^{***}	-2.852^{***}	-2.427^{***}	-3.468^{***}	-1.489^{***}	-2.721^{***}	-2.062^{***}	-2.982^{***}
	(0.182)	(1.036)	(0.185)	(0.210)	(0.155)	(0.144)	(0.216)	(0.275)	(0.167)	(0.216)
Dispersion	6.116	104.083	3.255	2.643	4.054	1.794	5.194	4.927	5.718	5.041
Observations	79	79	79	79	79	79	79	79	79	79
Note:	$^{*}p<0.1$; $^{**}p<0.05$; $^{***}p<0.01$									
