# Supplementary material for: Characterizing gene tree conflict in plastome-inferred phylogenies
Source: PeerJ. 2019 Sep 24;7:e7747. doi: 10.7717/peerj.7747 (PMC6764362; doi:10.7717/peerj.7747)
Supplement: Table S5 — Quasibinomial logistic regression results for a range of datasets, excluding highly influential genes based on studentized residuals, leverage and Cook’s distance and including all predictors, both ignoring and considering BS ≥ 70% support. Parameters are not transformed i.e. they represent the estimated ceteris paribus effect of the predictor on log odds. Quantities in brackets are standard errors. Dispersion gives the estimated quasibinomial dispersion parameter. The genes ycf1 and ycf2 were uniformly removed due to their long tree and alignment length. Other genes removed are as follows: FSA AA cln, FSA AA cln BS ≥ 70: rpl32, high tree length. FSA nuc cln: clpP, poor concordance relative to length ; rps15, relatively high tree length. FSA nuc cln BS ≥ 70: as not considering support, but clpP is included. MAFFT AA: ndhJ, high concordance relative to length; rpl22, high tree length; rpl32, high tree length. MAFFT AA BS ≥ 70: rpoB, highly concordant relative to length and tree length. MAFFT nuc: clpP, poor concordance relative to alignment and tree length. MAFFT nuc BS ≥ 70: rps15, poor performance relative to tree length. WAG: rpl32, high tree length; rpoC2, very high concordance. WAG BS ≥ 70: rpoC2, very high concordance. [file peerj-07-7747-s010.pdf]

Table S5: Quasibinomial logistic regression results for a range of datasets, excluding highly influential genes based on studentized residuals, leverage and Cook's distance and including all predictors, both ignoring and considering BS ≥ 70% support. Parameters are not transformed i.e. they represent the estimated ceteris paribus effect of the predictor on log odds. Quantities in brackets are standard errors. Dispersion gives the estimated quasibinomial dispersion parameter. The genes *ycf1* and *ycf2* were uniformly removed due to their long tree and alignment length. Other genes removed are as follows: FSA AA cln, FSA AA cln BS ≥ 70: *rpl32*, high tree length. FSA nuc cln: clpP, poor concordance relative to length; *rps15*, relatively high tree length. FSA nuc cln BS ≥ 70: as not considering support, but *clpP* is included. MAFFT AA: *ndhJ*, high concordance relative to length; *rpl22*, high tree length; *rpl32*, high tree length. MAFFT AA BS ≥ 70: *rpoB*, highly concordant relative to length and tree length. MAFFT nuc: *clpP*, poor concordance relative to alignment and tree length. MAFFT nuc BS ≥ 70: *rps15*, poor performance relative to tree length. WAG: *rpl32*, high tree length; *rpoC2*, very high concordance. WAG BS ≥ 70: *rpoC2*, very high concordance.

	Dependent variable: Total Concordant/Total Discordant									
	FSA cln AA	FSA cln AA BS ≥ 70	FSA cln nuc	FSA cln nuc BS ≥ 70	MAFFT AA	MAFFT AA BS ≥ 70	MAFFT nuc	MAFFT nuc BS ≥ 70	WAG	WAG BS ≥ 70
alignment_length	0.002^{***}	0.002^{***}	0.001^{***}	0.001^{***}	0.002^{***}	0.002^{***}	0.001^{***}	0.001^{***}	0.002^{***}	0.002^{***}
	(0.0003)	(0.0003)	(0.0001)	(0.0001)	(0.0003)	(0.0003)	(0.0001)	(0.0001)	(0.0003)	(0.0003)
tree_length	0.709^{***}	0.709^{***}	1.133^{***}	1.063^{***}	0.703^{***}	0.545^{***}	0.760^{***}	0.819^{***}	0.636^{***}	0.536^{***}
	(0.106)	(0.106)	(0.219)	(0.174)	(0.095)	(0.089)	(0.165)	(0.154)	(0.111)	(0.087)
variance	-51.572^{**}	-51.572^{**}	-214.123^{**}	-174.957^{***}	-57.590^{***}	-36.888^{***}	-85.038^{**}	-92.043^{***}	-55.714^{***}	-32.252^{***}
	(19.572)	(19.572)	(92.790)	(56.550)	(15.331)	(12.453)	(37.736)	(27.252)	(19.996)	(11.672)
Constant	-2.715^{***}	-2.715^{***}	-1.949^{***}	-1.927^{***}	-2.736^{***}	-2.580^{***}	-1.800^{***}	-1.840^{***}	-2.562^{***}	-2.517^{***}
	(0.152)	(0.152)	(0.173)	(0.167)	(0.145)	(0.151)	(0.175)	(0.173)	(0.146)	(0.141)
Dispersion	3	3	2.371	2.352	2.666	3.319	2.838	2.761	2.875	2.898
Observations	76	76	75	76	74	76	76	76	75	76

Note: $^{*}p<0.1$; $^{**}p<0.05$; $^{***}p<0.01$
