# Supplementary material for: Characterizing gene tree conflict in plastome-inferred phylogenies
Source: PeerJ. 2019 Sep 24;7:e7747. doi: 10.7717/peerj.7747 (PMC6764362; doi:10.7717/peerj.7747)
Supplement: Table S14 — Binomial logistic regression results for all genes from both amino acid and nucleotide datasets, including all predictors, both ignoring and considering BS ≥ 70% support. Parameters are not transformed, i.e. they represent the estimated ceteris paribus effect of the predictor on log odds. Quantities in brackets are standard errors. [file peerj-07-7747-s019.pdf]

Table S14: Binomial logistic regression results for all genes from both amino acid and nucleotide datasets, including all predictors, both ignoring and considering BS ≥ 70% support. Parameters are not transformed, i.e. they represent the estimated ceteris paribus effect of the predictor on log odds. Quantities in brackets are standard errors.

	Dependent variable: Total Concordant/Total Discordant			
	logistic			
	AA	AA BS ≥ 70	Nuc	Nuc BS ≥ 70
Length	0.0002***	0.0002***	0.0002***	0.0002***
	(0.00004)	(0.00004)	(0.00002)	(0.00002)
Tree_Length	0.589***	0.701***	1.306***	1.649***
	(0.046)	(0.063)	(0.104)	(0.127)
Root-to-tip Variance	-41.119***	-74.377***	-310.777***	-504.933***
	(5.551)	(10.768)	(34.320)	(43.698)
Constant	-2.012***	-2.968***	-1.618***	-2.732***
	(0.069)	(0.093)	(0.101)	(0.127)
Observations	79	79	79	79
Log Likelihood	-383.756	-249.108	-341.953	-312.632
Akaike Inf. Crit.	775.513	506.217	691.906	633.263

Note: *p<0.1; **p<0.05; ***p<0.01
