# Supplementary material for: Characterizing gene tree conflict in plastome-inferred phylogenies
Source: PeerJ. 2019 Sep 24;7:e7747. doi: 10.7717/peerj.7747 (PMC6764362; doi:10.7717/peerj.7747)
Supplement: Table S15 — Binomial logistic regression results for a range of datasets, excluding highly influential genes based on studentized residuals, leverage and Cook’s distance and including all predictors, both ignoring and considering BS ≥ 70% support. Parameters are not transformed i.e. they represent the estimated ceteris paribus effect of the predictor on log odds. Quantities in brackets are standard errors. For details of genes removed, see main text. [file peerj-07-7747-s020.pdf]

Table S15: Binomial logistic regression results for a range of datasets, excluding highly influential genes based on studentized residuals, leverage and Cook's distance and including all predictors, both ignoring and considering BS ≥ 70% support. Parameters are not transformed i.e. they represent the estimated *ceteris paribus* effect of the predictor on log odds. Quantities in brackets are standard errors. For details of genes removed, see main text.

	Dependent variable: Total Concordant/Total Discordant			
	logistic			
	AA	AA BS ≥ 70	Nuc	Nuc BS ≥ 70
Length	0.002^{***}	0.002^{***}	0.001^{***}	0.001^{***}
	(0.0001)	(0.0002)	(0.0001)	(0.0001)
Tree_Length	0.772^{***}	0.677^{***}	1.138^{***}	1.138^{***}
	(0.068)	(0.083)	(0.145)	(0.145)
Root-to-tip Variance	-98.944^{***}	-76.416^{***}	-234.793^{***}	-234.793^{***}
	(15.412)	(20.333)	(55.806)	(55.806)
Constant	-2.546^{***}	-3.557^{***}	-1.894^{***}	-1.894^{***}
	(0.083)	(0.111)	(0.113)	(0.113)
Observations	75	75	75	75
Log Likelihood	-257.248	-156.195	-253.832	-253.832
Akaike Inf. Crit.	522.496	320.391	515.665	515.665
Note:	$^{*}p<0.1$; $^{**}p<0.05$; $^{***}p<0.01$			
