# Supplementary material for: Characterizing gene tree conflict in plastome-inferred phylogenies
Source: PeerJ. 2019 Sep 24;7:e7747. doi: 10.7717/peerj.7747 (PMC6764362; doi:10.7717/peerj.7747)
Supplement: Table S16 — Quasibinomial logistic regression results for all genes from both amino acid and nucleotide datasets, including all predictors, both ignoring and considering BS ≥ 70% support. Parameters are not transformed i.e. they represent the estimated ceteris paribus effect of the predictor on log odds. Quantities in brackets are standard errors. Dispersion gives the estimated quasibinomial dispersion parameter. [file peerj-07-7747-s021.pdf]

**Table S16**: Quasibinomial logistic regression results for all genes from both amino acid and nucleotide datasets, including all predictors, both ignoring and considering BS ≥ 70% support. Parameters are not transformed i.e. they represent the estimated ceteris paribus effect of the predictor on log odds. Quantities in brackets are standard errors. Dispersion gives the estimated quasibinomial dispersion parameter.

| | Dependent variable: Total Concordant/Total Discordant | | | |
|---|---|---|---|---|
| | *glm: quasibinomial* | | | |
| | *link = logit* | | | |
| | AA | AA BS ≥ 70 | Nuc | Nuc BS ≥ 70 |
| Length | 0.0002** | 0.0002* | 0.0002*** | 0.0002*** |
| | (0.0001) | (0.0001) | (0.00004) | (0.00004) |
| Tree_Length | 0.589*** | 0.701*** | 1.306*** | 1.649*** |
| | (0.123) | (0.186) | (0.222) | (0.266) |
| Root-to-tip Variance | -41.119*** | -74.377** | -310.777*** | -504.933*** |
| | (14.787) | (31.532) | (73.500) | (91.484) |
| Constant | -2.012*** | -2.968*** | -1.618*** | -2.732*** |
| | (0.183) | (0.273) | (0.215) | (0.266) |
| Dispersion | 7.096 | 8.575 | 4.587 | 4.383 |
| Observations | 79 | 79 | 79 | 79 |
| *Note:* | *p<0.1; **p<0.05; ***p<0.01 | | | |
