# Supplementary material for: Characterizing gene tree conflict in plastome-inferred phylogenies
Source: PeerJ. 2019 Sep 24;7:e7747. doi: 10.7717/peerj.7747 (PMC6764362; doi:10.7717/peerj.7747)
Supplement: Table S18 — Binomial logistic regression results for amino acid and nucleotide datasets, including only alignment length and tree length, both ignoring and considering BS ≥ 70% support. Parameters are not transformed i.e. they represent the estimated ceteris paribus effect of the predictor on log odds. [file peerj-07-7747-s023.pdf]

Table S18: Binomial logistic regression results for amino acid and nucleotide datasets, including only alignment length and tree length, both ignoring and considering BS ≥ 70% support. Parameters are not transformed i.e. they represent the estimated ceteris paribus effect of the predictor on log odds.

	Dependent Variable: Total Concordant/Total Discordant			
	logistic			
	AA	AA BS ≥ 70	Nuc	Nuc BS ≥ 70
Length	0.0002^{***}	0.0002^{***}	0.0001^{***}	0.0001^{***}
	(0.00004)	(0.00003)	(0.00002)	(0.00001)
Tree_Length	0.266^{***}	0.223^{***}	0.567^{***}	0.412^{***}
	(0.027)	(0.032)	(0.063)	(0.068)
Constant	-1.756^{***}	-2.575^{***}	-1.187^{***}	-1.867^{***}
	(0.060)	(0.076)	(0.087)	(0.097)
Observations	79	79	79	79
Log Likelihood	-430.988	-295.026	-390.554	-395.112
Akaike Inf. Crit.	867.975	596.053	787.109	796.225
Note:	*p<0.1; **p<0.05; ***p<0.01			
