# Supplementary material for: Characterizing gene tree conflict in plastome-inferred phylogenies
Source: PeerJ. 2019 Sep 24;7:e7747. doi: 10.7717/peerj.7747 (PMC6764362; doi:10.7717/peerj.7747)
Supplement: Table S19 — Binomial logistic regression results for amino acid and nucleotide datasets, excluding ycf1 and ycf2 as highly influential observations, including only alignment length and tree length and both ignoring and considering BS ≥ 70% support. Parameters are not transformed, i.e. they represent the estimate ceteris paribus effect of the predictor on log odds. Quantities in brackets are standard errors. [file peerj-07-7747-s024.pdf]

Table S19: Binomial logistic regression results for amino acid and nucleotide datasets, excluding *ycf1* and *ycf2* as highly influential observations, including only alignment length and tree length and both ignoring and considering BS ≥ 70% support. Parameters are not transformed, i.e. they represent the estimate ceteris paribus effect of the predictor on log odds. Quantities in brackets are standard errors.

	Dependent Variable: Total Concordant/Total Discordant			
	logistic			
	AA	AA BS ≥ 70	Nuc	Nuc BS ≥ 70
Length	0.002^{***}	0.002^{***}	0.001^{***}	0.001^{***}
	(0.0001)	(0.0002)	(0.00005)	(0.00005)
Tree_Length	0.291^{***}	0.277^{***}	0.509^{***}	0.414^{***}
	(0.029)	(0.035)	(0.067)	(0.075)
Constant	-2.390^{***}	-3.414^{***}	-1.580^{***}	-2.516^{***}
	(0.077)	(0.105)	(0.095)	(0.113)
Observations	77	77	77	77
Log Likelihood	-296.628	-177.738	-279.551	-248.075
Akaike Inf. Crit.	599.256	361.476	565.103	502.151
Note:	$^{*}p<0.1$; $^{**}p<0.05$; $^{***}p<0.01$			
