# Supplementary material for: Characterizing gene tree conflict in plastome-inferred phylogenies
Source: PeerJ. 2019 Sep 24;7:e7747. doi: 10.7717/peerj.7747 (PMC6764362; doi:10.7717/peerj.7747)

Figure S4: Network graph analysis of RF distances between gene trees and species trees. Nodes are colored according to alignment length of the corresponding gene, or white if they represent species trees or concatenated alignments. Alignment lengths of *ycf1* and *ycf2* were trimmed to approximately the length of *rpoC2*. Edge widths and coloring represent RF distances; thicker edges correspond to stronger similarities (e.g. smaller distances). The graph was laid out using the Fruchterman-Reingold algorithm with inverse RF for edge weights.

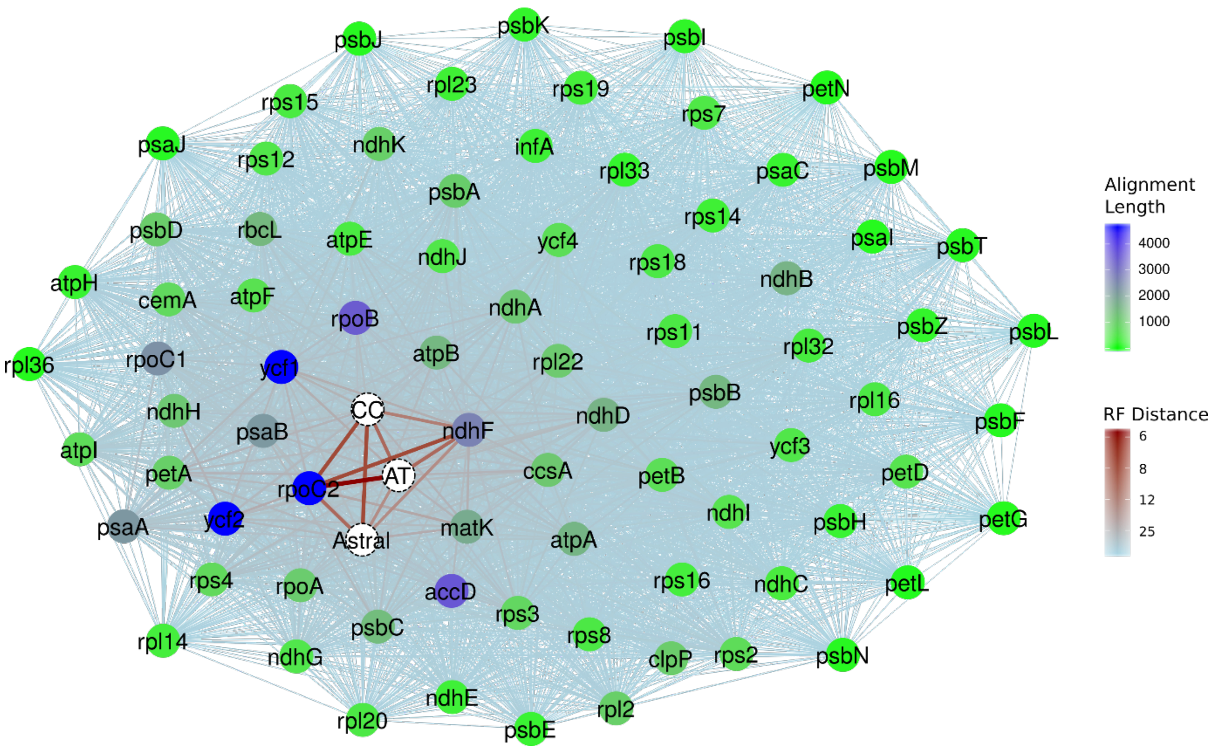

Supplement: Figure S4 — Network graph analysis of RF distances between gene trees and species trees. Nodes are colored according to alignment length of the corresponding gene, or white if they represent species trees or concatenated alignments. Alignment lengths of ycf1 and ycf2 were trimmed to approximately the length of rpoC2. Edge widths and coloring represent RF distances; thicker edges correspond to stronger similarities (e.g., smaller distances). The graph was laid out using the Fruchterman-Reingold algorithm with inverse RF for edge weights. [file peerj-07-7747-s004.pdf]
