# Supplementary material for: Characterizing gene tree conflict in plastome-inferred phylogenies
Source: PeerJ. 2019 Sep 24;7:e7747. doi: 10.7717/peerj.7747 (PMC6764362; doi:10.7717/peerj.7747)
Supplement: Table S21 — Quasibinomial logistic regression results for amino acid and nucleotide datasets excluding ycf1 and ycf2 as highly influential observations, including only length and tree length and both ignoring and considering BS ≥ 70% support. Parameters are not transformed i.e. they represent the estimated ceteris paribus effect of the predictor on log odds. Quantities in brackets are standard errors. Dispersion gives the estimated quasibinomial dispersion parameter. [file peerj-07-7747-s026.pdf]

Table S21: Quasibinomial logistic regression results for amino acid and nucleotide datasets excluding *ycf1* and *ycf2* as highly influential observations, including only length and tree length and both ignoring and considering BS ≥ 70% support. Parameters are not transformed i.e. they represent the estimated ceteris paribus effect of the predictor on log odds. Quantities in brackets are standard errors. Dispersion gives the estimated quasibinomial dispersion parameter.

	Dependent Variable: Total Concordant/Total Discordant			
	glm: quasibinomial *link = logit*			
	AA	AA BS ≥ 70	Nuc	Nuc BS ≥ 70
Length	0.002^{***}	0.002^{***}	0.001^{***}	0.001^{***}
	(0.0003)	(0.0002)	(0.0001)	(0.0001)
Tree_Length	0.291^{***}	0.277^{***}	0.509^{***}	0.414^{***}
	(0.059)	(0.046)	(0.121)	(0.127)
Constant	-2.390^{***}	-3.414^{***}	-1.580^{***}	-2.516^{***}
	(0.158)	(0.137)	(0.171)	(0.190)
Dispersion	4.25	1.684	3.277	2.841
Observations	77	77	77	77
Note:	$^{*}p<0.1$; $^{**}p<0.05$; $^{***}p<0.01$			
