# Supplementary material for: Characterizing gene tree conflict in plastome-inferred phylogenies
Source: PeerJ. 2019 Sep 24;7:e7747. doi: 10.7717/peerj.7747 (PMC6764362; doi:10.7717/peerj.7747)
Supplement: Table S20 — Quasibinomial logistic regression results for amino acid and nucleotide datasets, including only alignment length and tree length, both ignoring and considering BS ≥ 70% support. Parameters are not transformed i.e. they represent the estimated ceteris paribus effect of the predictor on log odds. Dispersion gives the estimated quasibinomial dispersion parameter. [file peerj-07-7747-s025.pdf]

Table S20: Quasibinomial logistic regression results for amino acid and nucleotide datasets, including only alignment length and tree length, both ignoring and considering BS ≥ 70% support. Parameters are not transformed i.e. they represent the estimated ceteris paribus effect of the predictor on log odds. Dispersion gives the estimated quasibinomial dispersion parameter.

	Dependent Variable: Total Concordant/Total Discordant			
	glm: quasibinomial *link = logit*			
	AA	AA BS ≥ 70	Nuc	Nuc BS ≥ 70
Length	0.0002**	0.0002**	0.0001***	0.0001***
	(0.0001)	(0.0001)	(0.0001)	(0.00004)
Tree_Length	0.266***	0.223***	0.567***	0.412**
	(0.076)	(0.072)	(0.166)	(0.176)
Constant	-1.756***	-2.575***	-1.187***	-1.867***
	(0.168)	(0.173)	(0.229)	(0.249)
Dispersion	7.827	5.175	6.862	6.621
Observations	79	79	79	79
Note:	*p<0.1; **p<0.05; ***p<0.01			
